# Enhancing cell type deconvolution in spatial transcriptomics through incorporating multiple reference panels

**DOI:** 10.1371/journal.pgen.1012345

**Published:** 2026-11-02

**Authors:** Peiyao Zhao, Justin A. Colacino, Ying Ma, Xiang Zhou

**Affiliations:** 1 Department of Biostatistics, School of Public Health, University of Michigan, Ann Arbor, Michigan, United States of America; 2 Center for Statistical Genetics, University of Michigan, Ann Arbor, Michigan, United States of America; 3 Department of Environmental Health Sciences, University of Michigan School of Public Health, Ann Arbor, Michigan, United States of America; 4 Department of Nutritional Sciences, University of Michigan School of Public Health, Ann Arbor, Michigan, United States of America; 5 Department of Biostatistics, School of Public Health, Brown University, Providence, Rhode Island, United States of America; 6 Center for Computational Molecular Biology, Brown University, Providence, Rhode Island, United States of America; 7 Department of Statistics and Data Science, Yale University, New Haven, Connecticut, United States of America; The University of Iowa, UNITED STATES OF AMERICA

## Abstract

Spatial transcriptomics (ST) captures gene expression with spatial context but often at resolutions where each spatial location contains multiple cells. Deconvolution of cell-type proportions is therefore essential, yet existing methods typically rely on a single scRNA-seq reference, limiting accuracy when references are incomplete or mismatched especially in complex tissues. These limitations highlight the need for a framework that can integrate multiple references while leveraging spatial information. We introduce SWIMR (Spatially Weighted Integration for Multi-Reference deconvolution), a statistical framework that integrates multiple scRNA-seq references while accounting for spatial correlations. SWIMR models each spatial location as a weighted combination of references, with both cell-type proportions and reference weights varying across tissue locations. Across comprehensive simulations and diverse ST datasets, SWIMR consistently outperformed single-reference approaches. In developing human heart tissue, it recovered ground-truth cell-type compositions with the highest accuracy. In human lung and breast cancer, SWIMR delineated tumor, stromal, and immune compartments, uncovered tumor-adjacent populations, and detected cell type–specific spatially variable genes missed by other methods. In mouse embryo and liver cancer, it leveraged context-specific references to resolve organ-specific cell types and increase sensitivity for biologically relevant pathways. Together, these results establish SWIMR as a robust framework for multi-reference deconvolution in spatial transcriptomics.

## Author summary

Spatial transcriptomics measures gene expression at many locations across tissues, revolutionizing genomics. However, many widely used spatial technologies have limited resolution: each measured location may contain multiple cells, often from different cell types. Researchers therefore commonly use computational deconvolution methods to estimate cell-type composition at each location, typically using single-cell RNA-seq reference data. However, it is often difficult to find a single-cell reference that closely matches a spatial experiment in tissue type, developmental stage, disease state, or other characteristics. The growing availability of public single-cell datasets provides an opportunity to combine information from multiple related references. This, however, creates a new challenge: how to decide which reference, or combination of references, to use, as this choice can substantially affect deconvolution results.We developed SWIMR, a computational method that integrates multiple single-cell reference datasets for spatial transcriptomics deconvolution. SWIMR automatically learns the contribution of each reference and allows these contributions to vary across tissue locations, while leveraging spatial organization to stabilize cell-type estimates. Across simulations and datasets, SWIMR effectively integrates complementary reference information and produces biologically meaningful maps of cell-type distributions. SWIMR provides a flexible approach for leveraging the growing diversity of single-cell reference data for spatial deconvolution.

## Introduction

The spatial organization of cells is fundamental to tissue development, homeostasis, and disease [1]. Changes to this organization, such as those occurring in cancer, immune response, or embryogenesis, can profoundly affect tissue function and clinical outcomes. Spatial transcriptomics (ST) technologies allow researchers to profile gene expression in tissue sections while preserving spatial context, providing new opportunities to investigate how cell types are distributed and interact within their native microenvironments [2]. Among the available ST platforms, sequencing-based technologies [3–6] such as 10x Visium have gained widespread applicability due to their scalability, high-throughput, and acceptable experimental cost. However, these platforms typically operate at resolutions where each spatial location (ranging from ~2 to 100 μm in diameter) contains transcripts from multiple cells, leading to mixed gene expression profiles. As a result, interpreting ST data at cellular resolution requires computational deconvolution methods to estimate the proportions of cell types contributing to each spatial location, which is important for various downstream analyses such as the detection of cell type–specific spatially variable genes (ct-SVGs) [7,8].

To estimate cell type proportions, a growing number of deconvolution methods have been developed. These can be broadly classified into reference-based approaches, which leverage single-cell RNA sequencing (scRNA-seq) datasets as external references, and reference-free approaches [9–11], which rely solely on the ST data. Reference-based methods—including RCTD [12], CARD [13], and cell2location [14]—generally outperform reference-free methods when a well-matched scRNA-seq reference is available, as they can more effectively distinguish transcriptionally similar cell types [15]. In contrast, reference-free methods do not assign cell type labels to inferred components, limiting their utility in studies requiring biologically interpretable annotations. However, existing reference-based deconvolution methods typically assume that a single scRNA-seq reference fully captures the cellular composition of the ST dataset [16]. In practice, this assumption is often violated due to biological variability, technical batch effects, or differences in sequencing platforms [17,18]. Moreover, in many practical settings, such as studies involving archival tissues [19] or clinical cohorts [20], matched scRNA-seq references are unavailable, and researchers must rely on partially relevant public datasets. These mismatches introduce bias and reduce the accuracy and interpretability of cell type estimates. This limitation is particularly pronounced in complex tissues such as tumors or developing organs, where no single reference can fully represent the underlying cellular heterogeneity. While multi-reference deconvolution has proven successful in bulk RNA-seq analysis [21], it remains underexplored in spatial transcriptomics. Adapting a bulk multi-reference deconvolution strategy to spatial transcriptomics to enable accurate cell type deconvolution, however, requires the development of new methods that can account for the spatial correlation structures inherent in the data [22].

To address these challenges, we present SWIMR (Spatially Weighted Integration for Multi-Reference deconvolution), a statistical framework for robust cell type deconvolution in spatial transcriptomics. SWIMR models gene expression at each spatial location as a weighted combination of multiple references, with both the cell type proportions and reference weights assumed to vary spatially. To achieve this, SWIMR introduces a two-level conditional autoregressive (CAR) model [23] that integrates spatial information by encouraging local similarity across neighboring tissue locations. This spatially aware structure enables SWIMR to adaptively assign reference weights based on tissue context, mitigate biases from mismatched or incomplete references, and borrow strength from neighboring locations to enhance robustness. We benchmarked SWIMR through comprehensive simulations and applied it to a range of real spatial transcriptomics datasets, including developing human heart tissue, multi-organ mouse embryo sections, and tumor samples from human lung, breast and liver cancers. Across all settings, SWIMR consistently outperformed existing methods, improving accuracy in cell type composition estimation and revealing deeper biological insights, such as transitional or tumor-adjacent cell populations, enhanced detection of cell type–specific spatially variable genes, and enrichment of disease-relevant pathways. These results demonstrate that SWIMR offers a powerful and generalizable solution for deconvolution in spatial transcriptomics, particularly in settings with complex tissue architecture or uncertain reference quality.

## Results

### Overview of SWIMR

SWIMR is a spatial deconvolution method that integrates multiple scRNA-seq references while accounting for the spatial relationships among locations within tissue organizations. The technical details are provided in Methods and Note A in S1 Appendix, with an overview illustrated in Fig 1. At its core, SWIMR builds upon a non-negative matrix factorization (NMF) framework [24] by incorporating two levels of Conditional Autoregressive (CAR) assumption [23] that capture the spatial correlation structure for both cell type compositions and the contributions from each scRNA-seq reference. Specifically, the first CAR assumption reflects a fundamental principle of tissue biology: neighboring locations within a tissue tend to exhibit more similar cellular compositions than distant ones. By borrowing information from nearby locations, SWIMR enhances signal resolution while reducing the impact of local noise or artifacts in spatial transcriptomic data. The second CAR assumption is novel in the context of multi-reference integration. SWIMR models gene expression at each spatial location as a weighted combination of multiple scRNA-seq references and further assumes that these weights vary smoothly across space, which is more similar for neighboring locations than for distant ones. This design allows the model to adaptively emphasize the most contextually relevant references in different tissue regions, while leveraging the full diversity across references. In doing so, SWIMR accounts for technical and biological differences across references, mitigating reference bias and improving deconvolution robustness in the presence of mismatched or references with incomplete cell types. Together, these assumptions allow SWIMR to address the complexity of real-world spatial transcriptomics data analysis, where a single reference may be insufficient or poorly matched, by harmonizing diverse single-cell resources in a spatially informed manner. We developed efficient optimization strategies to iteratively estimate both the spatially varying cell type compositions and the reference weights, enabling scalable and accurate inference across large datasets.

**Fig 1:**
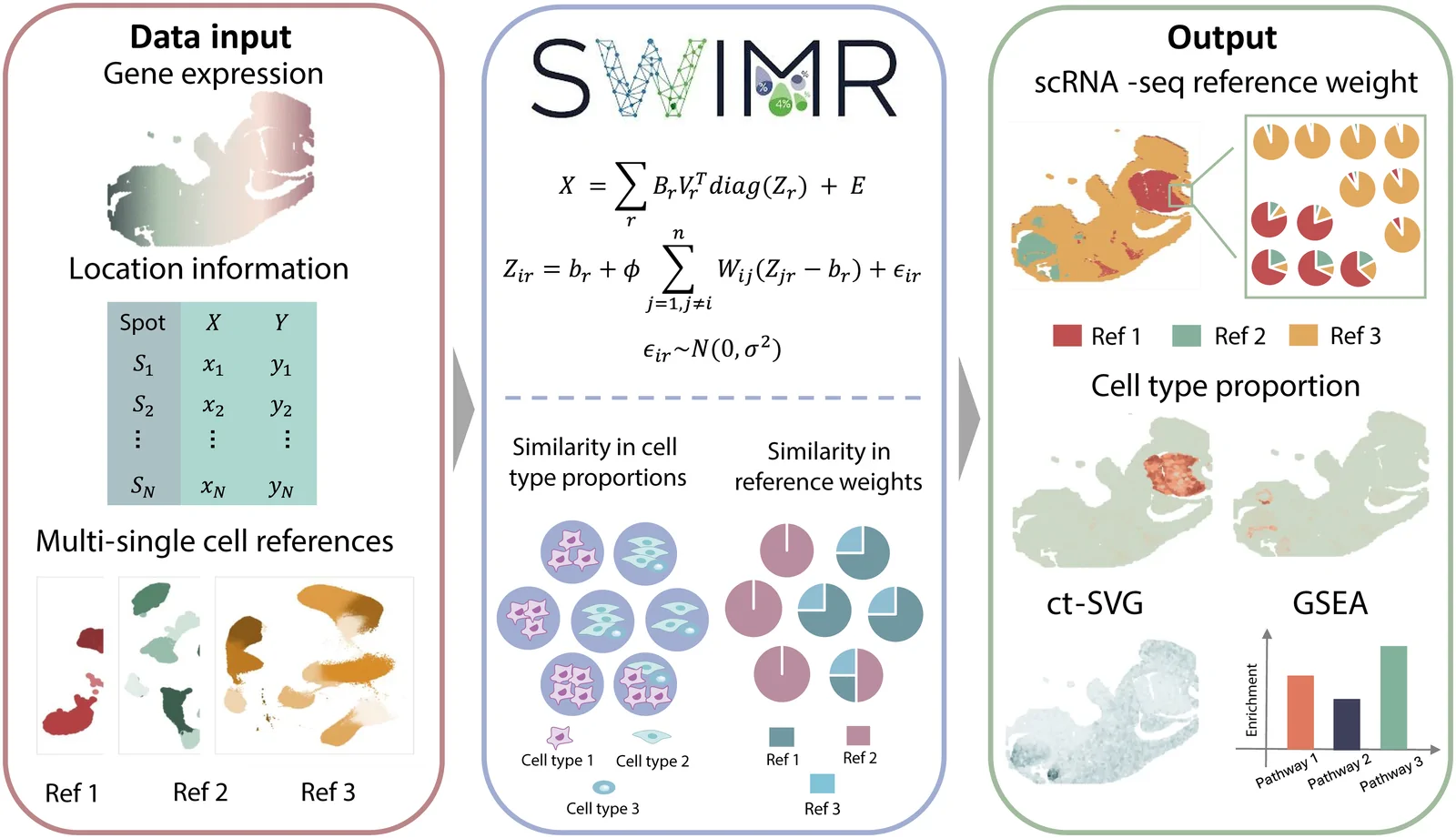
Overview of the SWIMR framework for multi-reference based deconvolution in spatial transcriptomics. SWIMR integrates multiple single-cell RNA-seq reference datasets to deconvolve spatial transcriptomics data. The framework takes as input both spatial transcriptomics gene expression and location information, alongside diverse single-cell RNA-seq references, each containing multiple cell types. Within the model, SWIMR employs a conditional autoregressive (CAR) approach that leverages spatial information to promote similarity both in the contribution (weights) of different references and in estimated cell type proportions across neighboring tissue locations. This spatial regularization produces spatially resolved estimates of reference weights and cell type proportions across the tissue, capturing local variation in cellular composition and reference contributions. These outputs serve as inputs for downstream analyses such as cell-type-specific spatially variable gene (ct-SVG) detection and gene set enrichment analysis (GSEA), enabling functional characterization of localized transcriptional patterns.

### Simulations

To systematically evaluate SWIMR’s performance, we conducted extensive and comprehensive simulations reflecting realistic challenges in spatial transcriptomics data analysis particularly those arising when multiple, partially overlapping scRNA-seq references are available. We benchmarked SWIMR against two categories of state-of-the-art deconvolution methods: (1) single-reference approaches such as CARD [13], RCTD [12], and cell2location [14], which can only process one scRNA-seq reference at a time. To enable comparison, we performed separate deconvolutions using each reference independently and denoted the results as CARD_ref1, CARD_ref2, RCTD_ref1, RCTD_ref2, cell2location_ref1, and cell2location_ref2. These methods were selected for benchmarking based on their consistent top performance in recent studies [15,16,25]. (2) multi-reference methods, represented solely by SCDC [21], the only available method capable of incorporating multiple scRNA-seq references, although it was originally developed for bulk RNA-seq deconvolution.

In brief, we simulated spatial transcriptomics data using two distinct scRNA-seq references [26,27] and evaluated performance across all methods (Methods, Note B and Fig A in S1 Appendix). Specifically, spatial coordinates were derived from the mouse olfactory bulb [28], a tissue with clearly defined structure consisting of the granule cell layer (GCL), the mitral cell layer (MCL), and the olfactory nerve layer (ONL). We then assigned one scRNA-seq reference to simulate gene expression in the GCL and ONL regions, and a second reference for the MCL region (Fig 2A). Each scRNA-seq reference contains six cell types, and cell type labels were consistent across references to allow direct comparison and controlled overlap. To capture the diversity of real-world biological scenarios, we varied the degree of overlap in cell types between the two references across three settings: complete overlap (all 6 cell types are shared), partial overlap (only two cell types are shared), and no overlap (0 shared cell types). Each setting was repeated 10 times. Performance was assessed by calculating the Root Mean Square Error (RMSE) between the estimated and true cell type proportions at each spatial location following Chen et al [29].

**Fig 2:**
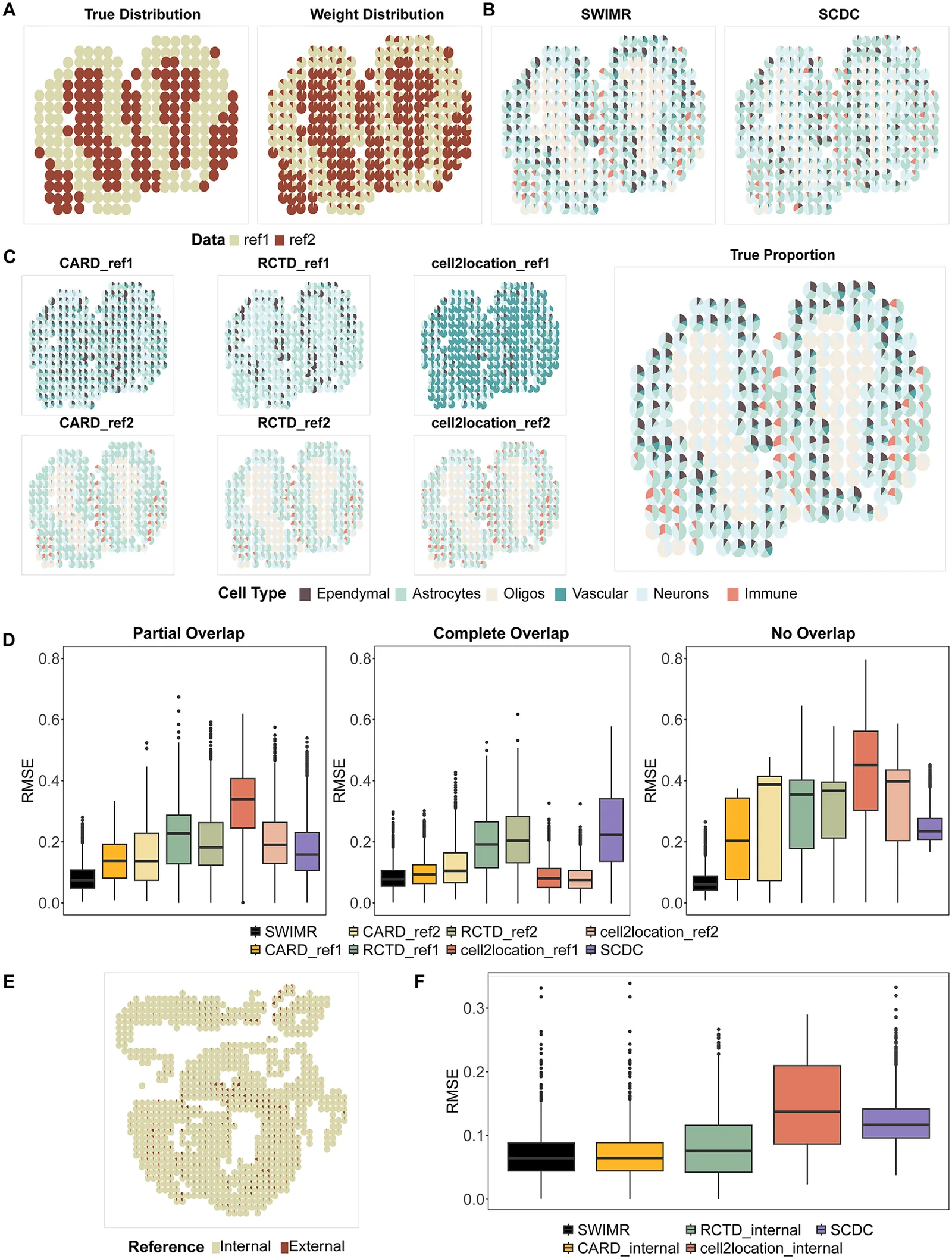
Simulation results (A–D) based on mouse olfactory bulb data and application to developing human heart data (E–F). **A.** True cell type distribution used to set up the simulation scenarios across two scRNA-seq reference datasets. **B.** Estimated cell type proportions by SWIMR, and SCDC, a method capable of incorporating multiple references for bulk RNA-seq deconvolution. **C.** Cell type compositions estimated by single-reference deconvolution methods including CARD, RCTD, and cell2location, alongside the true cell type proportions. **D.** Root mean squared error (RMSE) of cell type proportion estimates for all methods and simulation scenarios. **E.** Reference weight distributions assigned to internal and external scRNA-seq references by SWIMR. **F.** RMSE comparison for all deconvolution methods. Here, for single-reference methods, results using internal reference are shown. Boxplots show the median (center line), interquartile range (box edges), and whiskers extending to 1.5 × the IQR; points denote outliers.

We begin with the first scenario, where cell types are partially overlapped across references, which is the most common case in real word applications. Specifically, the two references share astrocytes and neuron cell types, while one reference uniquely contains vascular and ependymal cells and the other includes immune and oligodendrocytes cells. While SCDC assigns nearly equal weights to each reference regardless of their true contributions—allocating 47–49% (mean = 48.5%) to reference 1 and 51–53% (mean = 51.5%) to reference 2—SWIMR more accurately captures the relative contributions of each scRNA-seq reference. Moreover, the scatter pie plots of the estimated cell type compositions closely mirrored the ground truth layered structure (Fig 2A and 2B). Quantitatively, SWIMR achieved the lowest mean RMSE (0.082), outperforming CARD (ref1: 0.141, ref2: 0.158), RCTD (ref1: 0.214, ref2: 0.198), cell2location (ref1: 0.280, ref2: 0.207), and SCDC (0.177) (Fig 2C and 2D).

We next consider the less common complete overlap scenario, where both references shared all six cell types: neurons, astrocytes, oligodendrocytes, vascular cells, immune cells, and ependymal cells. Consistent with the first scenario, SWIMR accurately inferred the reference contributions across the anatomical layers (Fig B in S1 Appendix) and achieved comparable or better performance (mean RMSE = 0. 085) compared to CARD (ref1: 0.100, ref2: 0.121), RCTD (ref1: 0.195, ref2: 0.213), cell2location (ref1: 0.086, ref2: 0.081), and SCDC (0. 237) (Figs 2D and C in S1 Appendix). Moreover, SCDC allocating 50–52% (mean = 51%) to reference 1 and 48–50% (mean = 49%) to reference 2.

Finally, we evaluated a challenging case, the non-overlap scenario, where each reference contains a completely distinct set of cell types. Even in this setting, SWIMR remained robust. The inferred weight distributions closely matched the true simulation patterns (Fig B in S1 Appendix), and SWIMR achieved the lowest mean RMSE (0.070). In contrast, all other methods showed substantial performance degradation: CARD (ref1: 0.211, ref2: 0.259), RCTD (ref1: 0.295, ref2: 0.309), cell2location (ref1: 0.417, ref2: 0.308), and SCDC (0.247) (Figs 2D and Fig D in S1 Appendix). Moreover, SCDC allocating 49–51% (mean = 49.9%) to reference 1 and 49–51% (mean = 50.1%) to reference 2.

In summary, across all scenarios—partial overlap, complete overlap, and non overlap—SWIMR consistently outperformed existing methods in both accurately estimating cell type proportions and correctly distributing weights across references. This improvement is expected, as relying on one single scRNA-seq reference may fail to capture the full spectrum of cell types present in spatial transcriptomics data, particularly when reference overlap is limited. Additionally, spatial transcriptomics data have unique structural characteristics, and relying solely on one scRNA-seq reference may not align well with the data, resulting in suboptimal deconvolution performance.

### Benchmarking SWIMR performance on developing human heart data

To further evaluate SWIMR’s performance on real-world data, we applied it to a spatial transcriptomics dataset from the developing human heart, which was generated using *in situ* sequencing [30]. This dataset includes both spot-level and single-cell resolution ST data, along with matched scRNA-seq data. As all datasets were derived from biologically comparable samples with consistent cell type annotations, we followed previous studies [29] to treat the observed cell-type proportions in the single-cell level ST data as ground truth and use these to evaluate the estimated cell-type proportions. Specifically, two scRNA-seq datasets were used as references for deconvolution: one internal and one external. The internal reference was obtained directly from the original study^30^ and generated using the same ISS-based platform as the pseudo-spatial data; therefore, it contained the same set of cell types and was closely matched to the spatial transcriptomics dataset. In contrast, the external reference was generated using 10x Genomics scRNA-seq and manually annotated to assign cell-type labels. To assess consistency between the two references, we compared cell type–specific average expression profiles across matched cell types. Most shared cell types showed strong agreement, with correlations ranging from 0.72 to 0.89 for 9 of the 11 matched cell types. The highest correlations were observed for fibroblast-like cells, smooth muscle cells/fibroblast-like cells, subepicardial cells, and ventricular cardiomyocytes. Endothelium/pericytes/adventitia showed moderate agreement, whereas cardiac neural crest–related cells showed low consistency (Methods, Note C in S1 Appendix, and Fig E in S1 Appendix). Both references were used for deconvolution with SWIMR and SCDC. Other methods were evaluated using only the internal reference because it was more closely matched to the spatial transcriptomics data. SWIMR assigned nearly all reference weight to the internal scRNA-seq dataset (Fig 2E), indicating that the model correctly identified it as the more representative reference. This result demonstrates that SWIMR can preferentially select the most relevant reference even when the candidate references are broadly concordant for most major cell types, while remaining sensitive to reference-specific differences in several cell populations. In contrast, SCDC’s model assigns weights to each reference uniformly across all locations based on the inverse sum of squared errors, allocating 67% to the internal reference and 33% to the external reference. Quantitatively, SWIMR achieved the lowest mean RMSE of 0.071, outperforming all other methods (Figs 2F and F in S1 Appendix). Notably, SWIMR’s performance was comparable to CARD (mean RMSE = 0.071) when using only the internal scRNA-seq reference, highlighting its robustness even with additional noise potentially introduced by external reference. In contrast, RCTD (mean RMSE = 0.084), SCDC (mean RMSE = 0.124), and cell2location (mean RMSE = 0.146) all demonstrated reduced accuracy.

### Human Lung Cancer 10x Visium data

We next evaluated SWIMR using a spatial transcriptomics dataset from human lung cancer tissue profiled by the 10x Visium platform. For SWIMR, we use scRNA-seq references [31] from five datasets generated by different platforms, including 10x Genomics [32], BD-Rhapsody [33], inDrop [34], Singleron [35] and Smart-seq2 [36] (Methods and Note C in S1 Appendix). SCDC was excluded from all real data analysis because we failed to generate any results due to its heavy computational burden and large memory requirement. For example, when we applied SCDC to the smallest human breast cancer dataset with 2,518 spatial locations, it could not finish the analysis within 3 days. For single-reference-based methods, deconvolution was performed with each scRNA-seq reference data independently. We first examined the inferred reference weight distribution from SWIMR (Fig 3A), and we found that scRNA-seq references from Smart-seq2 and inDrop contributed most prominently to the deconvolution. This result aligns with the known strengths of these platforms. For example, Smart-seq2 offers high sensitivity and full-length transcript coverage, capturing the transcriptional complexity of the tumor microenvironment, while droplet-based technologies such as inDrop are effective for profiling diverse populations such as endothelial and B cells [37,38]. Because Smart-seq2 data were normalized and thus incompatible with count-based models, we selected the inDrop reference as the baseline for comparisons involving single-reference methods.

**Fig 3:**
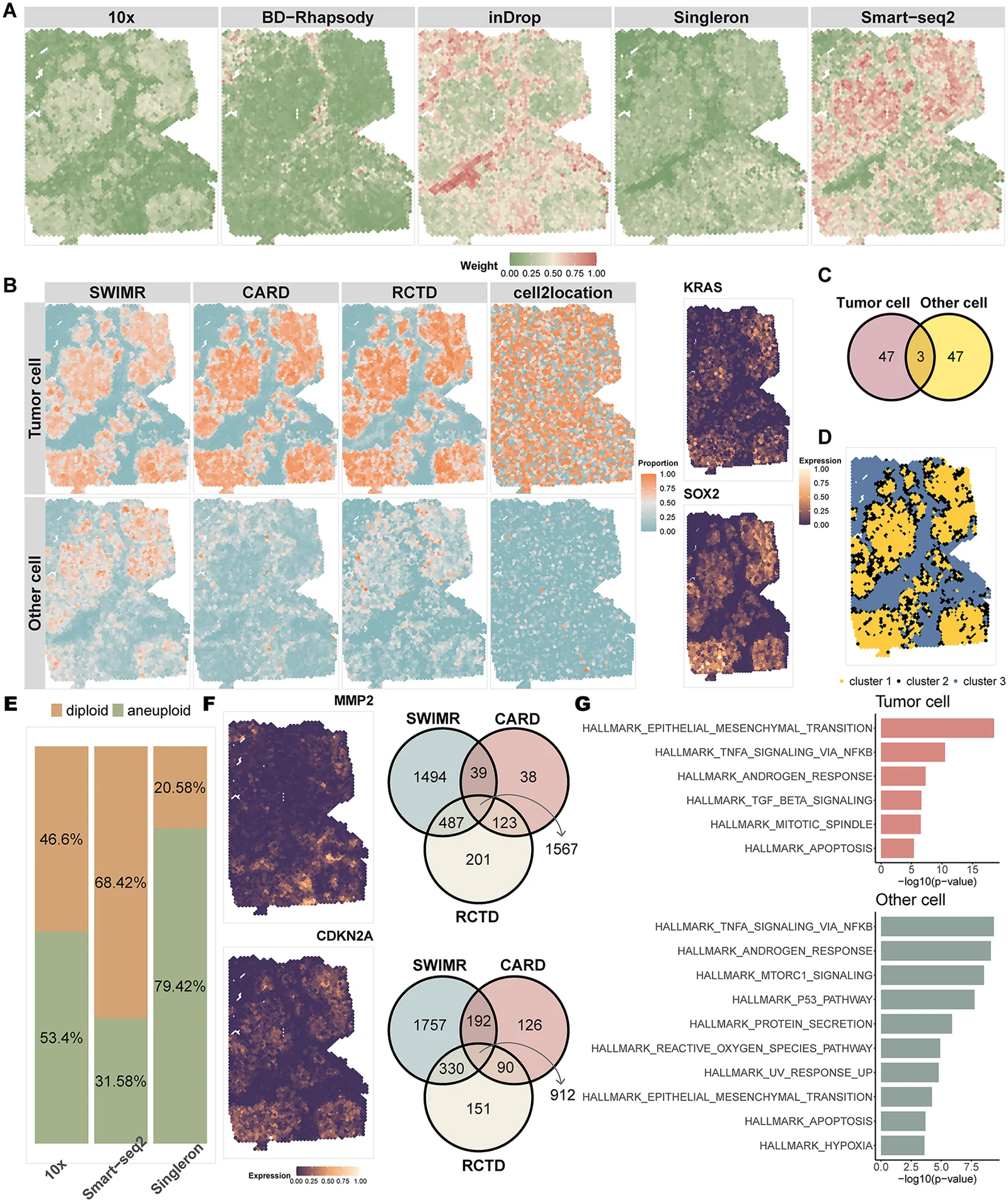
Analysis of human lung cancer 10x Visium data. **A**. Distribution of weights assigned to scRNA-seq reference datasets generated using different technologies. **B.** Cell type compositions inferred by each deconvolution method, shown alongside the corresponding expression patterns of cell type-specific marker genes. **C**. Venn diagram showing the overlap of the top 50 marker genes distinguishing tumor cells from other cell types. **D**. Correlation analysis between tumor cell and other cell type compositions, highlighting that the distribution of cluster 1 closely matches that of tumor cells. **E**. Proportions of aneuploid (malignant) cells identified by copyKAT across different scRNA-seq references. **F**. left: Uniquely detected cell type-specific spatially variable genes (ct-SVGs) for tumor cells (top) and other cells (bottom). Right: Venn diagrams comparing ct-SVGs identified by different methods for tumor cells (top) and other cells (bottom). **G**. Gene set enrichment analysis (GSEA) results based on ct-SVGs uniquely identified by SWIMR.

To further validate the inferred reference-weight pattern, we compared cell type-specific expression profiles across the scRNA-seq references (Fig G in S1 Appendix). For each cell type, we computed the average expression profile within each reference and evaluated the similarity between references using correlation analysis when the cell type was available. This analysis provides an independent way to assess whether references receiving higher weights in SWIMR better capture major cellular populations relevant to the lung cancer dataset. The results showed that reference similarity was cell-type dependent. InDrop showed the strongest representation for several key lung cancer-related populations, including tumor cells (0.781), macrophages (0.655), and monocytes (0.572), whereas Smart-seq2 showed the strongest representation for T cell populations, including CD4 T cells (0.575), CD8 T cells (0.582), and regulatory T cells (0.511). Other references showed stronger representation for specific cell types, such as BD-Rhapsody for alveolar macrophages, NK cells, and neutrophils, and Singleron for stromal, mast, plasma, and club cell populations. These results indicate that no single reference is uniformly optimal across all cell types, supporting the need for a multi-reference framework. They also help explain why inDrop and Smart-seq2 contributed prominently to SWIMR deconvolution, as these references better captured major tumor and immune compartments in the lung cancer dataset.

Next, we evaluated the spatial distribution of cell type proportions across the tissue. We found that SWIMR, CARD, and RCTD all captured similar tumor regions and other major regions, whereas cell2location failed to detect any pattern in the tumor region or any other regions. To assess the tumor regions, we analyzed the expression of *KRAS*, a well-known oncogene that promotes cell proliferation and survival and serves as a recognized marker of tumorigenesis [39]. Its expression pattern aligned well with the tumor region that was enriched with high tumor cell types (Fig 3B). Comparisons of cell type distributions across SWIMR, CARD, and RCTD (Figs 3B and H–K in S1 Appendix) showed general agreement regarding the composition of major cell types. However, notable differences emerged in the detection of minor and ambiguous cell types. For example, RCTD failed to estimate proportions for several minor cell types, including Alveolar cell type 1, ciliated cell, club cell, and cDC1 cells while SWIMR and other methods successfully captured these populations (Figs H-K in S1 Appendix). Importantly, SWIMR uniquely identified a previously undefined population, labeled as the “other” cell type, which was highly concentrated near tumor-dense regions (Fig 3B). To further characterize this population, we re-analyzed the scRNA-seq references using Seurat [40] and identified marker genes unique to the annotated “other” cells and tumor cells. Among the top 15 markers for the “other” cell type population were well-known tumor-associated genes, including *SOX2*, *ALDH3A1*, and *AKR1C1* [41–43]. Notably, *SOX2* (Fig 3B) plays a critical oncogenic role in small-cell lung cancer, influencing stem cell functions and driving tumor proliferation [41]. These genes were strongly enriched in tumor regions, suggesting that SWIMR had uncovered a transcriptionally distinct, tumor-adjacent population with possible functional relevance. To further explore the relationship between “other” and tumor cells, we compared the top 50 marker genes from each group and found only 3 genes in common, indicating significant transcriptional differences between these two cell types (Fig 3C). Additionally, k-means clustering of these two cell type proportions further supported this difference. Specifically, there is a negative correlation of the cell type proportions (r = –0.227) between tumor and “other” cells within tumor-enriched regions (Fig 3D, cluster 1). This further supports that the annotated “other” cells represent a distinct population with tumor-like features. To determine whether there are malignant (aneuploid) tumor cells from normal (diploid) cells within “other” cells, we perform analysis using the CopyKAT [44] tool on each scRNA-seq reference (Methods) and found that three of the five references contained a significant proportion of aneuploid cells categorized as “other” (53.40% for 10x, 31.58% for Smart-seq2, and 79.42% for Singleron), suggesting that these “other” cells may be associated with the malignant tumor compartment (Fig 3E).

We further investigated whether SWIMR improves detection of cell type–specific spatially variable genes (ct-SVGs), focusing on the tumor cell population (Fig 3F). Since cell2location did not yield clear tumor cell compositions, it was excluded from this analysis. Specifically, we applied CELINA [45] to identify ct-SVGs based on cell type proportions inferred by SWIMR, CARD, and RCTD. SWIMR uniquely identified ct-SVGs with known roles in lung cancer progression. Notably, *MMP2*, detected only by SWIMR under tumor cells, is important for tumor invasion and metastasis [46], while *CDKN2A*, identified in the “other” cell population, is frequently altered in lung cancer and contributes to cell cycle dysregulation [47]. We then performed gene set enrichment analysis (GSEA) on the ct-SVGs (Fig 3G) and found that genes uniquely identified by SWIMR were significantly enriched in multiple pathways relevant to lung cancer (Table B in S1 Appendix). Notably, HALLMARK_APOPTOSIS (p-value = 0.005 for tumor, p-value = 0.025 for “other”) and HALLMARK_TNFA_SIGNALING_VIA_NFKB (p-value = 2.754e-05 for tumor, p-value = 8.546e-05 for “other”) emerged from ct-SVGs exclusively detected in both tumor and “other” cells. These pathways are known to play important roles in lung cancer, with changes in apoptosis helping cancer cells survive and TNFα-NFκB signaling linked to tumor growth and inflammation [48]. Furthermore, ct-SVGs identified from “other” cells were enriched in HALLMARK_ANDROGEN_RESPONSE (p-value = 1.090e-04) and HALLMARK_MTORC1_SIGNALING (p-value = 1.933e-04), both of which have been reported in lung cancer–related transcriptional programs [49,50]. By contrast, no enriched pathways were detected using the ct-SVGs identified exclusively by the other methods. In summary, SWIMR not only improves deconvolution accuracy but also reveals subtle, spatially coherent, tumor-relevant cell populations and gene signatures that are missed by existing methods. Its ability to extract nuanced biological signals from multi-reference data makes it particularly powerful for dissecting the complexity of tumor microenvironments.

### Developing Mouse Embryo 10x Visium data

We next applied SWIMR to a spatial transcriptomics dataset from a developing mouse embryo, profiled using the 10x Visium platform. To perform deconvolution, we incorporated three organ-specific scRNA-seq references—brain, liver, and heart tissues—derived from E14.5 mouse embryos (Methods) [51]. The spatial distribution of reference weights inferred by SWIMR aligned strikingly well with anatomical expectations: brain-derived reference weights dominated in the neural region, liver-derived references were localized to the visceral region, and heart-derived references concentrated around the prospective cardiac region (Fig 4A). At this developmental stage, the prospective cardiac region is composed primarily of mesodermal progenitors with strong stromal and muscle-like transcriptional programs; thus, the enrichment of heart-derived references likely reflects shared mesodermal signatures rather than fully differentiated cardiomyocytes. This spatial concordance demonstrates SWIMR’s ability to selectively leverage relevant references in a context-specific and spatially coherent manner. To verify these results, we examined the expression patterns of known organ-specific marker genes (Fig 4B). For example, the liver marker *Alb* (albumin), known for its role in maintaining osmotic pressure and binding various molecules, exhibited strong expression in the visceral region [52]. Similarly, brain markers *Foxg1* and *Six6* were predominantly expressed in the embryonic neural region, consistent with their critical functions in forebrain and retinal development, respectively [53,54]. In the prospective cardiac region, we observed high expression of *Ttn* (titin), a structural protein central to cardiac muscle contraction [55]. Although *Ttn* typically features in later stages of heart development, its presence here indicates mesodermal precursors destined to form the heart. Given that each scRNA-seq reference was derived from a single organ, we also evaluated the performance of single-reference methods (CARD, RCTD, and cell2location) by applying them one reference at a time.

**Fig 4:**
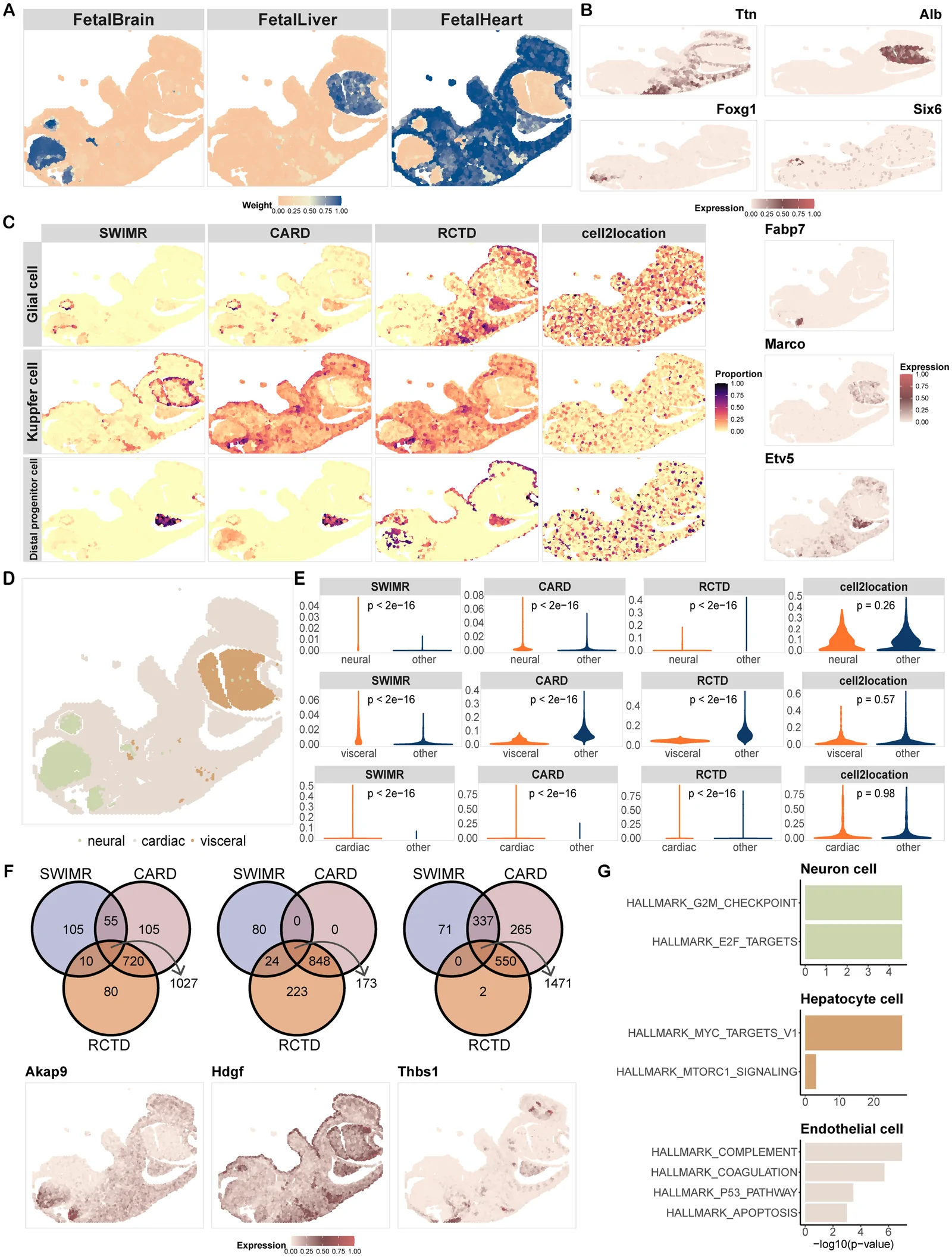
Analysis of developing mouse embryo 10x Visium data. **A**. Weight distributions assigned to scRNA-seq references derived from distinct anatomical regions of the mouse embryo. **B**. Expression patterns of four region-specific marker genes: *Ttn* for the cardiac region, *Alb* for the visceral region, *Foxg1* and *Six6* for neural region. **C**. Cell type compositions inferred by each deconvolution method, shown alongside the expression profiles of corresponding cell type-specific marker genes. **D**. Clustering outcome of the inferred reference weights, which closely aligns with the three anatomical regions: neural, visceral, and cardiac. **E**. Violin plots comparing inferred cell type proportions between the target region and other regions; Wilcoxon rank-sum tests were performed to assess differences in distributions. The top, middle, and bottom rows correspond to comparisons for the neural, visceral, and prospective cardiac regions, respectively. **F**. Upper: Venn diagrams comparing ct-SVGs identified by different methods for neuron cells (left), hepatocyte cells (middle) and endothelial cells (right). Lower: Uniquely detected cell type-specific spatially variable genes (ct-SVGs) for neuron cells (left), hepatocyte cells (middle) and endothelial cells (right). **G**. Gene set enrichment analysis (GSEA) results based on ct-SVGs uniquely identified by SWIMR.

We first compared methods in localizing major organ-specific cell types. Specifically, we focused on glial cells, a neural progenitor population characterized by high expression of *Fabp7* (Fatty acid-binding protein 7), a gene essential for early neurodevelopment (Fig 4C) [56]. SWIMR precisely localized these cells to neural regions, in agreement with their expected spatial identity. In contrast, RCTD and cell2location misassigned these cells to cardiac regions. While CARD showed glial cell enrichment in neural regions, it also erroneously predicted their presence within cardiac territories, suggesting limited spatial specificity. We then examined Kupffer cells, specialized macrophages that reside predominantly in the visceral region, where they regulate immune tolerance and mediate apoptotic cell clearance during development [57]. We assessed their distribution using the marker gene *Marco*, whose expression is restricted to the visceral domain at developing stage [58]. SWIMR accurately detected Kupffer cells as enriched within the visceral region, fully concordant with *Marco* expression patterns. In contrast, RCTD, CARD, and cell2location incorrectly assigned high proportions of these cells to all anatomical structures that are not in the visceral region, failing to capture their organ-specific localization. Finally, we assessed distal progenitor cells, which contribute to cardiac and vascular lineage commitment, thereby supporting heart morphogenesis [59]. The expression of *Etv5*, a known marker of these progenitors that sustains progenitor identity and promotes proliferation during early cardiac development [60], was found to be spatially restricted to the cardiac region. SWIMR, CARD, and RCTD all identified enrichment of distal progenitors in cardiac regions, though CARD and RCTD also showed modest off-target enrichment in neural regions. In contrast, cell2location yielded a diffuse pattern lacking clear spatial specificity. To further evaluate organ-specific localization, we compared cell type proportions across distinct anatomical regions (Fig 4D–4E and Methods). For glial cells, SWIMR and CARD correctly predicted higher abundance in neural regions, whereas RCTD produced an inverse pattern and cell2location failed to detect any spatial signal. For Kupffer cells, only SWIMR recapitulated their visceral localization; all other methods misallocated them to cardiac regions. For distal progenitor cells, SWIMR, CARD, and RCTD recovered their enrichment in cardiac regions, though only SWIMR avoided misassignment to unrelated tissues. Notably, cell2location failed to differentiate their distribution between cardiac and neural regions.

We next examined methods in localizing minor organ-specific cell types, where single-reference methods often struggle. (Figs L-W in S1 Appendix). For instance, microglia, derived from yolk sac progenitors that colonize the brain [61], were misassigned to the liver region by RCTD, while SWIMR localized them correctly to neural regions. (Figs L-O in S1 Appendix). Similarly, SWIMR accurately enriched pre-B cells—originating from the fetal liver during mid-gestation—specifically in the liver [62], while CARD and cell2location incorrectly distributed them across non-hepatic regions of the embryo, and RCTD excluded them due to their low abundance (Figs P-S in S1 Appendix). Together, these findings underscore SWIMR’s superior capacity to reconstruct spatially accurate cell-type maps especially minor cell types in the developing embryo. By leveraging a spatially aware, multi-reference framework, SWIMR minimizes erroneous cross-organ assignments and provides a more biologically coherent view of cellular composition during early organogenesis.

To further evaluate SWIMR’s capacity for biologically meaningful spatial resolution, we assessed its ability to detect ct-SVGs. We focused on three representative cell types, each corresponding to a distinct organ system in the developing mouse embryo: neurons (neural region), hepatocytes (visceral region), and endothelial cells (cardiac region). Using SWIMR-derived cell type proportions, we identified 105, 80, and 71 unique ct-SVGs for neuron, hepatocyte, and endothelial cells, respectively (Fig 4F). Although CARD and RCTD also uniquely detected ct-SVGs, downstream GSEA analysis revealed enrichment of only one single pathway from these sets, suggesting limited biological relevance and a higher likelihood of false positives. In contrast, ct-SVGs detected exclusively by SWIMR demonstrated strong functional coherence (Fig 4F). For example, *Akap9* (A-kinase anchor protein 9), a neuron-specific SWIMR-identified ct-SVG, encodes a scaffold protein that coordinates cAMP-dependent signaling essential for neuronal polarity, synaptic organization, and brain development [63,64]. *Hdgf* (Hepatoma-derived growth factor), detected in hepatocytes, functions as a mitogenic and angiogenic factor that supports hepatic proliferation and organ morphogenesis in the developing visceral region [65]. Similarly, *Thbs1* (Thrombospondin 1), identified in endothelial cells, plays a key role in cardiac tissue remodeling and angiogenesis, critical for heart development [66]. GSEA of SWIMR-specific ct-SVGs revealed enrichment in hallmark pathways that are essential for cell fate determination and organogenesis (Fig 4G). In the neural lineage, ct-SVGs were enriched for HALLMARK_E2F_TARGETS (p-value = 0.01) and HALLMARK_G2M_CHECKPOINT (p-value = 0.01), reflecting active cell proliferation—a fundamental process in embryonic brain development [67,68]. For hepatocytes, enrichment in HALLMARK_MYC_TARGETS_V1 (p-value = 5.34e-13) and HALLMARK_MTORC1_SIGNALING (p-value = 0.04) highlights MYC-driven growth and metabolic activation necessary for liver expansion and function during fetal development [69]. In endothelial cells, pathways such as HALLMARK_COMPLEMENT (p-value = 0.001), HALLMARK_COAGULATION (p-value = 0.003), HALLMARK_P53_PATHWAY (p-value = 0.032), and HALLMARK_APOPTOSIS (p-value = 0.049) were enriched, indicating active vascular remodeling, immune signaling, and cell cycle regulation, which are all crucial for proper cardiac morphogenesis [70–72]. In contrast, RCTD uniquely detected enrichment of only HALLMARK_NOTCH_SIGNALING in neurons (p-value = 0.050), and no other methods yielded ct-SVGs associated with similarly robust developmental pathways. These findings underscore SWIMR’s enhanced sensitivity and specificity in identifying biologically meaningful spatial gene expression patterns. By capturing organ- and cell type–specific transcriptional programs, SWIMR offers a powerful framework for resolving complex cellular architecture and regulatory mechanisms driving mouse embryonic development.

### Human Breast Cancer 10x Visium data

To further evaluate SWIMR’s performance, we applied it to a human breast cancer spatial transcriptomics dataset generated by the 10x Visium platform along with the provided annotation (Fig 5A). For deconvolution, we selected four scRNA-seq references from different studies [73]. The distribution of reference weights closely aligns with the primary focus of each scRNA-seq study (Fig 5B). The reference from Pal B et al. [74] focuses on malignant epithelial cells and tumor-associated immune populations, which is consistent with the stronger weights observed in the invasive carcinoma regions (green in Fig 5A). In contrast, the Liu et al. [75] scRNA-seq reference encompasses a broad repertoire of epithelial, stromal, and immune cell types derived from both tumor and surrounding normal tissues, leading to higher weights in non-malignant regions, particularly in areas annotated as immune cells (purple) and fibrous tissue (red). The Xu et al. [76] reference includes both tumorigenic and non-tumorigenic subpopulations from breast cancer cell lines, as well as cancer-associated fibroblasts (CAFs), resulting in elevated weights within fibrous tissue. Overall, references from Liu et al. and Xu et al. display greater weighting in stromal compartments. Although the reference from Wu et al. [77] provides a comprehensive atlas of malignant, stromal, and immune compartments in breast cancer, its weights in our data are more concentrated within invasive carcinoma regions, potentially reflecting stronger alignment between its malignant profiles and the tumor transcriptomic signature in this sample. According to the inferred reference weight distribution (Fig 5B), the Liu et al. dataset contributed mostly, indicating it as the most contextually relevant reference. Consequently, we benchmarked SWIMR’s performance primarily against methods relying on this single reference.

**Fig 5:**
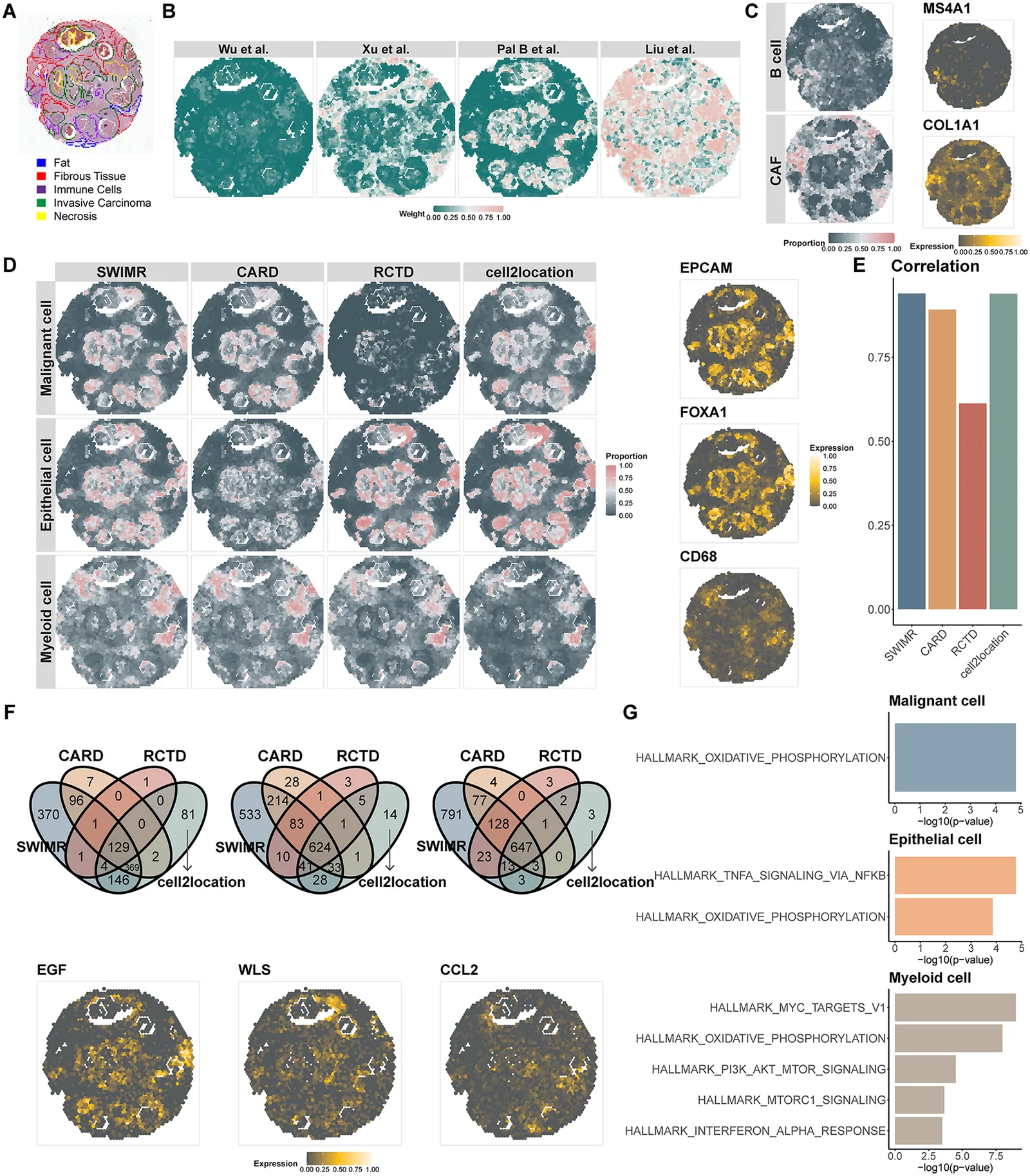
Analysis of human breast cancer 10x Visium data. **A.** Pathology annotation of tissue sections as obtained from 10x Visium. **B**. Weight distributions assigned to scRNA-seq reference datasets derived from different research studies. **C**. Expression patterns of two minor cell type marker genes: *MS4A1* for B cells and *COL1A1* for cancer-associated fibroblast cells. **D**. Cell type compositions inferred by each deconvolution method, shown alongside the expression profiles of corresponding cell type-specific marker genes. **E**. Spearman correlation analysis of cell type proportions between malignant cells and epithelial cells inferred by different methods. **F**. Upper: Venn diagrams comparing ct-SVGs identified by different methods for malignant cells (left), epithelial cells (middle) and myeloid cells (right). Lower: Uniquely detected cell type-specific spatially variable genes (ct-SVGs) for malignant cells (left), epithelial cells (middle) and myeloid cells (right). **G**. Gene set enrichment analysis (GSEA) results based on ct-SVGs uniquely identified by SWIMR.

Notably, only five cell types were shared across all four scRNA-seq references, while the remaining cell types were present in only one or a subset of references (Figs X-AA in S1 Appendix). This diversity presented a valuable test case for assessing SWIMR’s ability to leverage reference-specific information. To explore this, we first focused on two cell types - B cells and cancer-associated fibroblasts (CAFs) - that appeared in only a subset of references (Fig 5C). Despite their limited representation, SWIMR successfully recovered spatial distributions for these rare cell types that aligned well with canonical marker expression. Specifically, *MS4A1*, a well-known B cell marker gene, showed strong spatial concordance with B-cell proportions estimated by SWIMR (Fig 5C) [78]. *MS4A1*-positive B cells are linked to anti-tumor immune responses, with their functional roles varying by tumor subtype and immune contexture. Similarly, *COL1A1*, a hallmark CAF marker (Fig 5C) exhibited strong spatial alignment with SWIMR-inferred CAF distributions [79]. As a key extracellular matrix component, *COL1A1* promotes tumor progression through matrix remodeling, angiogenesis, and immunosuppression, and its elevated expression in breast cancer is associated with poor prognosis. For the single reference-based methods, while CARD and RCTD tend to detect more similar patterns aligned with the marker gene, cell2location, however, tends to detect too wide or narrow region comparing to the marker gene distribution (Figs Y-AA in S1 Appendix). Together, these examples underscore SWIMR’s robustness in localizing biologically meaningful cell types, even when they are only partially represented in the scRNA-seq references.

We next examined two epithelial lineages, which include both malignant and normal epithelial cells. Although all methods yielded broadly similar spatial patterns for these populations, CARD and RCTD tended to artificially separate them into distinct, non-overlapping domains (Fig 5D). To explore their relationships, we assessed the expression of key tumor-associated marker genes. *EPCAM*, an epithelial marker reported to be overexpressed in metastatic breast cancer, was enriched in regions identified by SWIMR as malignant epithelial cell zones [80]. In contrast, *FOXA1*, a transcription factor linked to luminal differentiation and less aggressive breast cancer subtypes [81], showed co-expression in the same areas, highlighting the subtle transcriptional overlap between malignant and epithelial compartments that SWIMR captured more precisely than competing methods. To further quantify this, we calculated the Spearman correlation of estimated cell type proportion between malignant cells and normal epithelial cells (Fig 5E). SWIMR achieved the highest correlation (0.940), followed closely by cell2location (0.939), while CARD and RCTD exhibited lower correlations of 0.892 and 0.613, respectively. Moreover, we examined myeloid cells, which contribute to immunosuppression, chronic inflammation, and tumor progression in the breast cancer microenvironment. SWIMR-inferred myeloid cell distributions closely matched the expression of *CD68*, a marker for tumor-associated macrophages and related myeloid populations (Fig 5D) [82]. While CARD and RCTD also successfully detected the region, cell2location failed to capture this pattern.

We further checked the ct-SVGs detected by SWIMR and other deconvolution methods, focusing on malignant, epithelial, and myeloid cells. SWIMR identified substantially more ct-SVGs for each of these cell populations than any other approach (Fig 5F). For example, in malignant cells, SWIMR detected 370 unique ct-SVGs, including *EGF*, which directly activates a potent growth-promoting signaling cascade [83]. In epithelial cells, SWIMR detected 533 ct-SVGs, including the marker gene *WLS* (Wnt Ligand Secretion Mediator), a key regulator of Wnt signaling frequently dysregulated in breast cancer [84]. SWIMR identified 791 unique ct-SVGs in myeloid cells, including *CCL2*, a chemokine known to recruit monocytes and macrophages into the tumor microenvironment, where they can significantly shape tumor progression in human breast cancer [85]. Gene set enrichment analysis (GSEA) of these ct-SVGs revealed pathways highly relevant to breast cancer biology (Fig 5G and Table C in S1 Appendix). For instance, SWIMR uncovered additional breast cancer-relevant pathways, including HALLMARK_TNFA_SIGNALING_VIA_NFKB (p-value = 0.009) and HALLMARK_OXIDATIVE_PHOSPHORYLATION (p-value = 0.021) in epithelial cells, as well as HALLMARK_INTERFERON_ALPHA_RESPONSE (p-value = 0.030). Notably, interferon-alpha signaling can play dual roles in breast cancer, promoting antitumor immunity or inflammation-driven tumor progression depending on the cellular context [86]. What is more, HALLMARK_OXIDATIVE_PHOSPHORYLATION (p-value = 0.008), uniquely enriched in myeloid cells by SWIMR, underscores the role of metabolic reprogramming in tumor-associated macrophages—a process implicated in breast cancer progression and immune modulation [87]. In contrast, HALLMARK_ADIPOGENESIS (p-value = 0.049) was exclusively detected by cell2location, but no other significant pathways were enriched among ct-SVGs identified uniquely by the remaining methods. Together, these findings illustrate how SWIMR’s broader detection of ct-SVGs can yield deeper insights into key signaling pathways implicated in breast cancer.

### Hepatocellular Carcinoma 10x Visium data

Finally, we applied SWIMR to analyze a 10x Visium spatial transcriptomics dataset of hepatocellular carcinoma (HCC). We curated three scRNA-seq reference datasets from independent studies, providing a heterogenous mix of immune, stromal, and malignant cell profiles (Methods). The inferred reference weight distribution aligns well with the annotation from the H&E image (Figs 6A and 6B). The Ma et al. [88] dataset offers detailed hepatocyte subtyping, including malignant and non-malignant populations, making it well-suited for hepatocyte deconvolution and thus is assigned most weight on tumor region. In contrast, Lu et al. [89] provides comprehensive annotation of stromal components, especially fibroblasts, enabling more accurate estimation of fibroblast proportions and hence is being assigned more weight on immune and CAF region. Sharma et al. [90] emphasizes fetal-like endothelial and macrophage populations, while informative for vascular and immune contexts, offers limited utility for hepatocyte or fibroblast profiling and therefore is assigned less weight. These results underscore the importance of performing deconvolution with different references for better deconvolution accuracy. We again compared SWIMR with the single-reference method based on the scRNA-seq dataset contributing the highest reference weight—specifically, the dataset from Ma et al.

**Fig 6:**
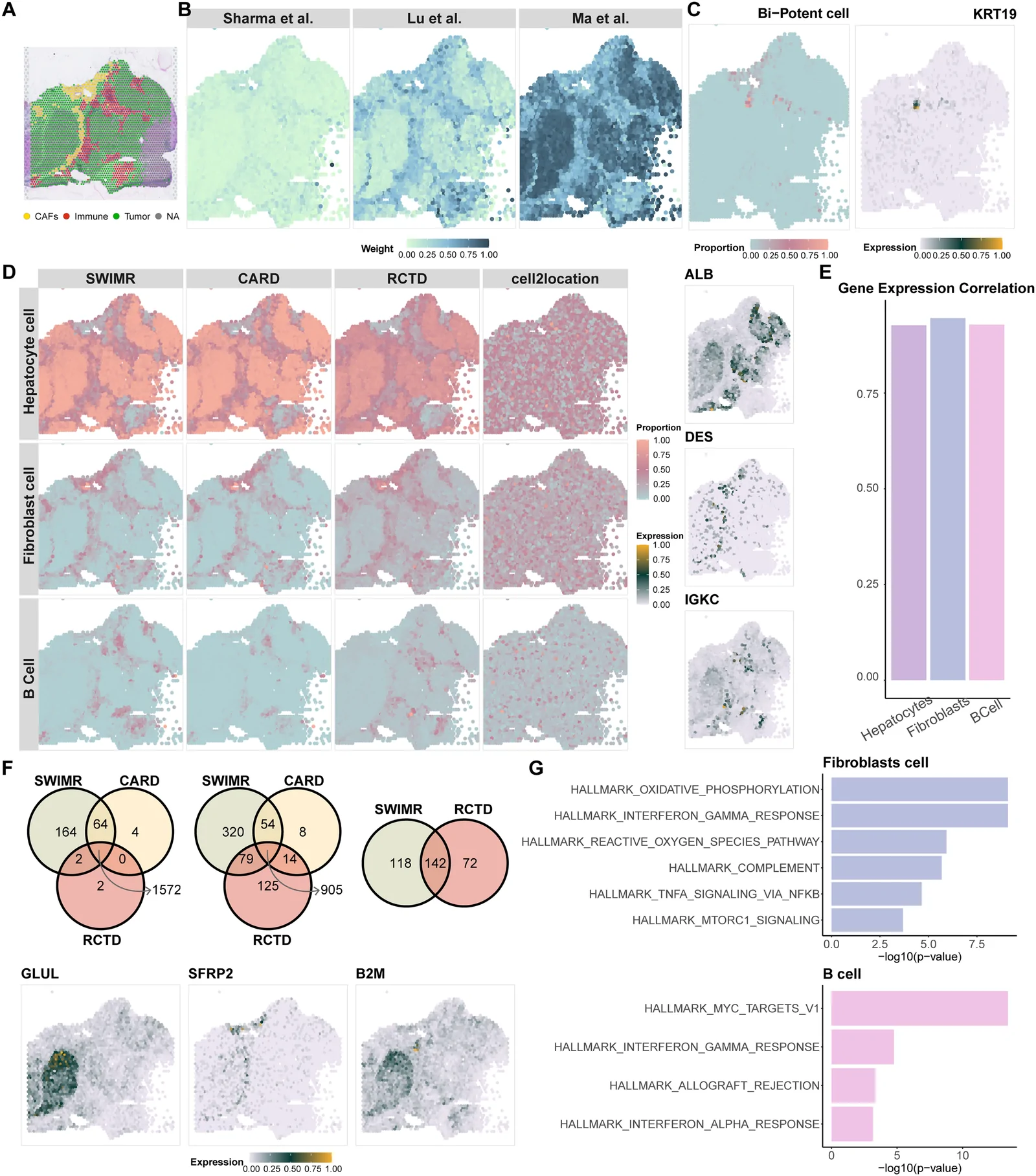
Analysis of hepatocellular carcinoma 10x Visium data. **A**. Hematoxylin and eosin (H&E) stained tissue image with original cell type annotations as provided by the source publication. **B**. Weight distributions assigned to scRNA-seq references derived from different research studies. **C**. Spatial patterns of cell type composition for bi-potent cells, and the spatial distribution of expression patterns of the corresponding marker gene *KRT19*. **D**. Cell type compositions inferred by each method, alongside the expression profiles of corresponding cell type-specific marker genes. **E**. Spearman correlation analysis of average cell type-specific gene expression across two scRNA-seq references: Ma et al. and Lu et al. **F**. Upper: Venn diagrams comparing ct-SVGs identified by different methods for hepatocyte cells (left) and fibroblast cells (right). Lower: Uniquely detected cell type-specific spatially variable genes (ct-SVGs) for hepatocyte cells (left) and fibroblast cells (right). **G**. Gene set enrichment analysis (GSEA) results based on ct-SVGs uniquely identified by SWIMR.

Notably, only one of the three references included minor cell types such as bi-potent cells. Although these minor cell types that were underrepresented in the references, they carry critical biological significance. For example, the expression of keratin genes (*KRT*), which are linked to cellular structural integrity and tumor progression in HCC, was highly colocalized with SWIMR’s estimates for bi-potent cells (Fig 6C) [91]. This correspondence supports SWIMR’s sensitivity to reference-specific signals and underscores the importance of incorporating rare but informative populations when interpreting tumor complexity (Figs AB-AE in S1 Appendix).

We took a deeper look at the cell type proportions and compared them to the single reference-based methods. In terms of the major cell type pattern detection, SWIMR, CARD and RCTD localized the hepatocytes cells into similar tumor regions while cell2location fails to produce meaningful cell type proportion estimates. Specifically, hepatocytes were the dominant population, and their marker *ALB*, which is critical for hepatic transport and often dysregulated in HCC, exhibited the expected tumor-centric pattern (Fig 6D) [92]. Fibroblast and B cells, though less abundant, are highly enriched in the immune/CAF zones: *DES* (desmin) marks activated fibroblasts involved in matrix remodeling, while *IGKC* denotes antibody-secreting B cells [93–95]. These two gene expression markers confirm that the fibroblast- and B-cell-rich spots recovered by SWIMR map to the appropriate stromal and immune regions. To understand why single-reference-methods also perform well in this data analysis, we compared the two scRNA-seq references: Ma et al. and Lu et al. since they contributed on average 94.12% of the deconvolution results. We found that cell-type–mean expression profiles among the two references were highly concordant (Spearman correlation = 0.93, 0.95 and 0.93 for hepatocytes cells, fibroblasts cells and B cells, Fig 6E), and therefore the performance of hepatocyte and fibroblast cells are very similar across methods meaning that despite using multiple references. SWIMR’s final composition most closely matched CARD’s single-reference output meaning that with more biologically similar scRNA-seq references, SWIMR provides as stable of cell type proportion estimates as those obtained from the single-reference method.

To evaluate whether integrating multiple references can yield novel biological insights despite high inter-reference similarity, we applied CELINA for ct-SVG analysis, focusing on hepatocytes, fibroblasts, and B cells (Fig 6F). We exclude the cell2location for comparison due to the lack of informative spatial patterns in its deconvolution results. B cells- analysis from CARD were additionally filtered out by CELINA due to their low abundance. Compared to single-reference approaches, SWIMR identified substantially more ct-SVGs, detecting 164 additional genes in hepatocytes, 320 in fibroblasts, and 118 in B cells. Notably, *GLUL* (glutamine synthetase)—a Wnt/β-catenin target and known driver of hepatocellular carcinoma (HCC) growth—was uniquely identified in hepatocytes [96]. In fibroblasts, SWIMR exclusively detected *SFRP2*, a modulator of the Wnt signaling pathway implicated in cancer-associated fibroblast activation and tumor–stroma interactions [97]. In B cells, SWIMR identified *B2M*, a gene essential for immune surveillance [98], underscoring the immune relevance of B cells in the HCC microenvironment. Gene set enrichment analysis (GSEA) of ct-SVGs revealed no significantly enriched pathways in hepatocytes across all methods. SWIMR-specific ct-SVGs enriched in six hallmark pathways in fibroblasts (Figs 6G and Table D in S1 Appendix), including TNFA_SIGNALING_VIA_NFKB (p-value = 0.010), INTERFERON_GAMMA_RESPONSE (p-value = 1.117e-04), and COMPLEMENT (p-value = 0.003), all linked to liver inflammation and HCC progression [99,100]. In contrast, ct-SVGs detected uniquely by single-reference methods did not enrich for additional pathways. Four hallmark pathways are also enriched only with the uniquely detected ct-SVGs from B cells with the cell type proportion from SWIMR. Pathways such as MYC_TARGETS_V1 (p-value = 1.422e-06) and INTERFERON_ALPHA_RESPONSE (p-value = 0.042), which are related to MYC oncogene activation that is associated with aggressive tumor growth in HCC [101]. What’s more, both HALLMARK_INTERFERON_GAMMA_RESPONSE (p-value = 8.502e-03) and HALLMARK_ALLOGRAFT_REJECTION (p-value = 0.035) reflect immune-related activity, and their enrichment in HCC suggests an inflamed tumor microenvironment with active immune signaling [102]. These results demonstrate that, even when scRNA-seq references are largely overlap, SWIMR extracts additional cell type specific spatially variable genes that uncover more biologically meaningful pathways in hepatocellular carcinoma.

## Discussion

We have presented a new computational framework, SWIMR, for robust cell type deconvolution in spatial transcriptomics by integrating multiple scRNA-seq references with spatially weighted modeling. By introducing a two-level conditional autoregressive assumption, SWIMR simultaneously captures the spatial coherence of cell type distributions and the context-dependent influence of each reference dataset. Across extensive simulations and real spatial transcriptomics datasets, including developing human heart tissue, diverse tumor microenvironments, and mouse embryonic development, SWIMR consistently outperformed existing methods, providing more accurate cell type proportion estimates and uncovering cell type–specific spatially variable genes (ct-SVGs) associated with key biological processes. Moreover, SWIMR remains computationally efficient despite incorporating multiple references and spatial dependence. Across three spatial transcriptomics datasets, SWIMR achieved runtime comparable to CARD while being 2.4–21.4 times faster than RCTD, 8.0–80.4 times faster than SCDC in datasets where SCDC completed, and 37.1–1,015.4 times faster than cell2location (Fig AF and Table E in S1 Appendix). To our knowledge, SWIMR represents the first spatial deconvolution method that adaptively reconciles multiple references, effectively addressing the limitations of single-reference methods and highlighting the value of integrating heterogeneous scRNA-seq datasets to improve spatial transcriptomics analysis.

To further distinguish SWIMR from a simpler multi-reference consensus strategy, we compared SWIMR with a consensus ensemble approach that independently deconvolves each reference and combines the resulting estimates using inverse reconstruction-error weights. Although this approach generally improved upon single-reference methods, it was generally less accurate than SWIMR (Note D and Figs AG-AH in S1 Appendix). These results support explicitly modeling spatially varying reference contributions rather than relying on single references or post hoc aggregation of independently fitted results.

We did not apply a uniform batch-correction procedure across references because some references had already been integrated in their original studies, whereas others underwent standard quality control and normalization, and aggressive alignment may remove meaningful biological variation. SWIMR does not directly remove batch effects but models references separately and allows less compatible references to receive smaller spatially varying weights. We therefore recommend applying batch correction when strong technical effects are suspected and, when feasible, comparing corrected and uncorrected references as a sensitivity analysis. Because SWIMR does not impose a hard exclusion rule for mismatched references, accurate deconvolution still requires high-quality, biologically relevant references that represent the cell types and transcriptional states present in the spatial dataset.

Cancer may particularly benefit from this framework because tumor transcriptomes vary across patients and molecular subtypes. Although the lung and breast cancer spatial datasets analyzed here each represented a single patient, an additional breast cancer analysis using HER2-positive, ER-positive, and TNBC subtype-specific references showed that SWIMR preserved the dominant tumor structure while recovering more spatially coherent secondary signals (Note E and Fig AI in S1 Appendix). Future multi-patient studies will be needed to directly assess patient-level variation in reference utilization.

SWIMR also has several limitations. First, its least-squares reconstruction loss on normalized expression does not explicitly model the discrete, sparse, and over-dispersed nature of spatial transcriptomics count data [18]. Future extensions could use Poisson or negative binomial objectives to better capture the mean–variance relationship, sparsity, and overdispersion of the original counts. Second, SWIMR currently uses a common spatial kernel and autocorrelation parameter for the cell-type proportion and reference-weight layers. Sensitivity analyses using different layer-specific spatial parameters produced only minor changes in performance, suggesting that the shared-parameter formulation is generally robust (Note F and Fig AJ in S1 Appendix). Nevertheless, layer-specific kernels or spatial autocorrelation parameters such as $\phi_{P}$ for the cell-type proportion layer and $\phi_{Q}$ for the reference-weight layer could be considered when the two layers exhibit substantially different spatial patterns.

Several additional extensions could broaden SWIMR's applicability. Alternative spatial structures incorporating histological or morphological information could improve deconvolution in complex tissues [103–105]. Such extensions could further improve the resolution of deconvolution in datasets with complex spatial architecture. Second, although we primarily focused on 10x Visium datasets in this study, SWIMR is readily applicable to other spatial transcriptomics technologies, including high-resolution or targeted panels, as long as sufficient overlap with reference genes is available [3,4,106]. Third, SWIMR could be extended to a hierarchical or multi-sample framework to jointly deconvolve multiple tissue sections, borrowing strength across replicates or patient samples while accounting for inter-sample variability [20,107].

Altogether, SWIMR advances the current landscape of spatial transcriptomics analysis by bridging multi-source data integration with spatially aware modeling. As spatial technologies continue to evolve, methods like SWIMR will be essential for extracting biologically meaningful insights from increasingly complex and heterogeneous data.

## Methods

We present an overview of SWIMR here, with its technical details provided in Note A in S1 Appendix. SWIMR requires two types of input: (1) multiple scRNA-seq datasets that serve as reference profiles and (2) ST data that measure gene expression on a tissue sample. The scRNA-seq reference data consists of R references, with the r-th specific scRNA-seq reference containing a set of $C_{r}$ cell types and G cell-type-informative genes. The set of G cell-type-specific informative genes are selected for each reference and the union of these genes across all references forms the final set. We denote Y as the library-size-normalized G-by-N gene expression matrix of ST data, where G represents the same set of informative genes across scRNA-seq references and N denotes the number of spatial locations. We model the ST gene expression matrix Y as a linear combination of reference-specific estimated spatial transcriptomics gene expressions $Y_{r}$, capturing the contribution of each reference in estimating the ST gene expression. Each $Y_{r}$ can be further modeled through a non-negative matrix factorization that links to the product of three components: (1) the cell type specific gene expression matrix from the r-th reference $S_{r}\in R^{G\times C_{r}}$, (2) the cell type proportion matrix contributed by the r-th reference $P_{r}\in R^{N\times C_{r}}$, and (3) a diagonal location-specific weight matrix for each reference, $Q_{r}\in R^{N\times N}$, where each diagonal element represents the contribution of r-th scRNA-seq reference to each spatial location. Specifically, our model is as follows:


$$\begin{array}{c} Y=\sum_{r}S_{r}P_{r}^{T}Q_{r}+E, \end{array}$$ (1)

where each element in $P_{r}$ and $Q_{r}$ is constrained to be non-negative, and the cell-type proportions in $P_{r}$ are further normalized across cell types at each spatial location. This constraint makes $P_{r}$ interpretable as cell-type composition rather than an arbitrary scaling factor. E is a G-by-N residual error with each element independently and identically following a normal distribution $E_{gn}\sim N(\text{0},\sigma_{e}^{\text{2}})$.

Previous studies [108–110] have demonstrated that cell type compositions derived from the scRNA-seq reference are similar in neighboring locations. Building on this observation, we model this spatial dependence using a conditional autoregressive (CAR) framework. For the composition of cell type k at location i from reference r, denoted $P_{i,r_{k}}$, we assume it can be expressed as weighted summation of the proportion of cell type k from r-th reference on all the other locations.


$$\begin{array}{c} P_{i,r_{k}}=s_{r_{k}}+\phi \sum_{j=\text{1},j\neq i}^{n}K_{ij}(P_{j,r_{k}}-s_{r_{k}})+\epsilon_{ir_{k}}, \end{array}$$ (2)

where $s_{r_{k}}$ denotes the k-th cell-type-specific intercept, which reflects the mean composition of the corresponding cell type across all locations. K is an N-by-N non-negative weight matrix representing the spatial relationship. $K_{ij}$, represents the weight from the j-th location used to infer the cell type composition on the i-th location. The parameter 𝜙 is a spatial autocorrelation parameter that determines the strength of the spatial correlation in cell type composition, and $\epsilon_{ir_{k}}$ is the residual error that follows a normal distribution.


$$\begin{array}{c} \epsilon_{ir_{k}}\sim N\left(\text{0},\sigma_{ir_{k}}^{\text{2}}\right). \end{array}$$

Following previous studies [13], we construct a Gaussian kernel, transform it into a standardized matrix, and then reparametrize this matrix to derive the location weight $K_{ij}$. With the above reparameterization, we can obtain the joint distribution for the N-sized vector $P_{r_{k}}$ based on Brook’s lemma as


$$\begin{array}{c} P_{r_{k}}\;\sim \text{MVN}\left(s_{r_{k}}1_{N},\Sigma_{r_{k}}^{\left(P\right)}\right), \end{array}$$ (3)

where $1_{N}$ is an N-vector of 1s, and $\Sigma_{r_{k}}^{\left(P\right)}$ is the positive-definite covariance matrix derived from the CAR specification discussed in Note A in S1 Appendix.

The similarity of the cell type composition also suggests that the contribution from each scRNA-seq reference is also similar in these neighboring areas. Based on this, our model assumes that both the estimated cell type compositions and the influence of each reference are spatially smooth, i.e., neighboring locations are likely to share similar reference weights. Specifically, we denote $Z_{r}$ as the diagonal element of $Q_{r}$, representing the r-th reference specific weight to the gene expression of each spatial location. We assume that the weight follows a CAR modeling assumption that for the r-th reference specific weight to location i, $Z_{ir}$, can be effectively expressed as a weighted summation of the reference specific weights on all the other locations.


$$\begin{array}{c} Z_{ir}=q_{r}+\phi \sum_{j=\text{1},j\neq i}^{n}K_{ij}(Z_{jr}-q_{r})+\epsilon_{ir}, \end{array}$$ (4)

where $q_{r}\;$ is the r-th reference-specific intercept, representing the average weight for the r-th scRNA-seq reference across locations. K is an N-by-N non-negative weight matrix representing the spatial relationship. To clearly differentiate between the weight assigned to references and the weight across spatial locations, we refer to the former as the reference weight $Q_{r}$ and the latter as the location weight K. Note that the location weight matrix K and the spatial correlation parameter 𝜙 are identical to those specified in equation (2). We use the same spatial kernel, constructed from the spatial coordinates with bandwidth κ, for both the cell-type proportion layer and the reference-weight layer because both are assumed to be shaped by the same underlying tissue architecture. Spatially adjacent spots are expected to have similar cell-type compositions, and they may also have similar reference relevance due to shared local histological context, transcriptional states, and cell-type organization. Accordingly, we use the same location weight matrix K to define neighborhood relationships and the same spatial autocorrelation parameter 𝜙 to control the strength of spatial smoothing in both layers. This shared specification provides a biologically motivated and parsimonious way to borrow information across neighboring locations while reducing model complexity. $\epsilon_{ir}$ is the residual error that follows a normal distribution.


$$\begin{array}{c} \epsilon_{ir}\sim N\left(\text{0},\sigma_{ir}^{\text{2}}\right). \end{array}$$

With the same procedure, we can obtain


$$\begin{array}{c} Z_{r}\sim MVN\left(q_{r}1_{N},\Sigma_{\text{r}}^{\left(Q\right)}\right), \end{array}$$ (5)

where $1_{N}$ is a vector containing N 1s, and $\Sigma_{\text{r}}^{\left(Q\right)}$ is the positive covariance matrix discussed in Note A in S1 Appendix.

Equations (1), (3), and (5) jointly define the multi-reference deconvolution model. The Gaussian assumptions in these equations are used as working models to induce quadratic loss and regularization terms: the Gaussian residual model in Equation (1) leads to a least-squares reconstruction loss, while the joint multivariate normal distributions in Equations (3) and (5) give rise to quadratic regularization terms for the reference-specific cell-type proportions and reference weights. The resulting estimation problem can therefore be formulated as a penalized nonnegative matrix factorization objective, subject to nonnegativity constraints on $P_{r}$ and $Q_{r}$ and an additional simplex constraint on $P_{r}$. The complete objective function, parameter constraints, and optimization procedure are provided in Note A in S1 Appendix. By incorporating the Conditional Autoregressive (CAR) assumptions in Equation (2) and (4), we integrate location information to infer the reference-specific cell type composition $P_{r}$ and reference-specific weights $Q_{r}$. This approach not only captures the contribution of each scRNA-seq reference to cell type composition but also accounts for spatial dependencies, improving the accuracy and biological relevance of deconvolution in spatial transcriptomics data. We developed a deterministic iterative optimization algorithm for a penalized non-negative matrix factorization objective that combines a least-squares reconstruction loss with CAR-based spatial regularization terms. The objective is optimized using multiplicative update rules under nonnegativity constraints on the reference-specific cell type compositions $P_{r_{k}}$ and reference-specific weights $Q_{r}$, or equivalently their diagonal elements $Z_{r}$, with an additional simplex constraint imposed on the cell-type proportions. In the final implementation, SWIMR uses a sequential plug-in strategy. First, the reference-specific cell-type proportion matrix $P_{r}$ is estimated for each reference under the nonnegativity, simplex, and spatial smoothness constraints. Second, the estimated $P_{r}$ matrices are fixed, and the reference weights $Q_{r}$ are estimated under nonnegativity and spatial smoothness constraints. Final cell type proportions are derived by computing a weighted combination of cell type compositions inferred from individual references, where the weights are estimated from the data by our model and normalized across references. This integration yields a more robust and spatially coherent representation of cell-type distributions.

### Compared methods

We compared SWIMR with four deconvolution methods: (1) CARD, (2) RCTD, (3) cell2location and (4) SCDC. For all methods, we followed the online tutorial, using either the default settings or the parameters recommended by the authors. Specifically, for RCTD, we considered doublet mode as suggested for all the analysis. For cell2location, for all the required additional parameters, we tried different settings and picked the one to be close to what we best know of in the real data applications. Specifically, we fixed N_cells_per_location to be 30 across all the analysis. For detection_alpha, we set it to be 200 for simulation and 20 for the real data analysis. In both simulated and real-world datasets, we extended the number of training epochs aiming for convergence. However, in several cases involving real data, the plots of the loss function suggest that convergence was not yet achieved or overfitted. Despite SCDC being designed for bulk RNA-seq, we included it by treating each spot as a sample since it is the only deconvolution method designed to adjust for multiple references. In SCDC, users are required to select a criterion for assigning weights to each reference dataset. In our implementation, we chose to determine the weights based on the inverse of the sum of squared errors. We successfully applied SCDC to the simulation and human heart datasets. However, when attempting to run SCDC on additional real datasets, the method failed to return results even after three days of computation. Due to these time constraints and scalability limitations, we excluded SCDC from the real-data comparisons in our study.

### Simulation and evaluation of the deconvolution methods

We simulated the spatial transcriptomics gene expression data by using three published datasets: two scRNA-seq datasets from the mouse nervous system (referred to as reference 1 [27] and reference 2 [26], described in the Note B in S1 Appendix) and one ST dataset from the mouse olfactory bulb. We used the two scRNA-seq references to simulate the ST data [3]. Specifically, we first categorized the 260 spatial locations obtained from the ST dataset into three anatomical regions: granule cell layer (GCL, 75 locations), mitral cell layer (MCL, 140 locations), and nerve layer (ONL, 45 locations) following [13]. Then, we utilized Reference 1 to simulate gene expression data for locations in the MCL, and Reference 2 for the GCL and ONL, aiming to generate spatial data for a comparable number of locations using both references. For each region, we simulated gene expression as follows. We first selected cell types colocalizing with the dominant cell type from a uniform distribution U (0, # of candidate cells -1), and then we sampled cell type proportions via a Dirichlet distribution [111] with a concentration parameter of 1.0. The total number of cells per location was fixed at 10 to mimic the 10x Visium technology, and gene expression levels were calculated by summing the expression of sampled cells from the six cell types in the scRNA-seq references. The final dataset was created by concatenating gene expression data from both references, assuming equal contributions from each. To evaluate robustness under varying degrees of reference similarity, we designed three simulation scenarios that differed in the number of overlapping cell types between Reference 1 and Reference 2:

1. Scenario I: Reference 1 and Reference 2 share all six cell types: neurons, astrocytes, oligodendrocytes, vascular cells, immune cells, and ependymal cells.
2. Scenario II: Both references share neurons and astrocytes, but Reference 1 uniquely contains vascular and ependymal cells, while Reference 2 has oligodendrocytes and immune cells.
3. Scenario III: Reference 1 and Reference 2 share no cell types. Reference 1 contains neurons, vascular, and ependymal cells, and Reference 2 contains astrocytes, oligodendrocytes, and immune cells.

In each scenario, we replicated the simulation procedure 10 times and for each replicate, we evaluated the performance of each deconvolution method by calculating the Root Mean Squared Error (RMSE). Specifically, we denote $V_{ik}$ and $\hat{V_{ik}}$ as the true and estimated cell type proportion for cell type i at spot k, and the RMSE is calculated as follows: $RMSE_{k}=\sqrt{\frac{\sum_{r}{\left(V_{ik}-\hat{V_{ik}}\right)}^{\text{2}}}{R}}$, where R is the total number of cell types in the i-th location.

### Real data analysis

All real datasets used in the present study are described in the Note C in S1 Appendix.

#### Human developing heart data.

We obtained the human developing heart data collected using *in situ* sequencing (ISS) technology. This dataset has both single-cell and spot-level Spatial Transcriptomics (ST) data from similar biological replicates (i.e., two human embryonic cardiac tissue sample at the same stage, with justification on checking the correlation among expression similarities in the original paper). The cell types match up in both the single-cell ST and scRNA-seq data. Because of this, we can use the cell-type proportions from the single-cell ST data as a baseline to check the accuracy of the spot-level data. We performed deconvolution with both internal and external references, where the internal reference is the one generated from ISS technology and the external reference is generated from 10x Genomics. It's worth mentioning that cell type annotations differ between our internal data and the external reference. To deal with this, we matched the cell types from the external data to our internal data by looking at which cell types had the most similar gene expression patterns in both internal and external scRNA-seq datasets. Since the correlation between Fibroblast-like (related to larger vascular development) and (8) Fibroblast-like (outflow tract & valve related) and the correlation between Fibroblast-like (related to larger vascular development) and (4) Fibroblast-like (coronary & mediastinal vasculature related) are 1, we simple combined the two cell types into one cell type, denoted as (15) Fibroblast-like (outflow tract & valve related & coronary & mediastinal vasculature related).

We compared our results with SCDC using multiple references, and other deconvolution methods that only use an internal reference.

#### Human lung cancer 10x Visium data.

We applied SWIMR to analyze lung cancer data using five references from different technologies (Note C in S1 Appendix) and compared it with other deconvolution methods that rely on a single scRNA-seq reference. SWIMR’s weight distribution heavily favored the Smart-seq2 scRNA-seq reference. However, due to the reference data from Smart-seq2 only provides the normalized gene expression, which is incompatible with most deconvolution methods, we opted for inDrop scRNA-seq data, which received the second-highest weighting. Our analysis revealed a notable similarity between the “other” cell patterns and tumor cells, a pattern absents in single-reference deconvolution outcomes. This prompted a re-examination of the single-cell references for these “other” and tumor cells to explore their interrelationship. We identified marker genes using the Seurat package [40], where we normalized the data with the NormalizeData function, scaled it using ScaleData function, conducted principal component analysis (PCA) with RunPCA function, and identified marker genes using the FindMarkers function for each cell type. Additionally, we employed the CopyKAT package [44] on each reference under default settings to determine if the annotated “other” cells included aneuploid cells, suggesting their potential significance in tumor studies.

#### Developing mouse embryo 10x Visium data.

We applied SWIMR to analyze developing mouse embryo data from 10X Visium, incorporating a stage E14.5 scRNA-seq reference (Note C in S1 Appendix). We benchmarked our results against those obtained from various methods using organ-specific scRNA-seq references, labeled as CARD_Brain, CARD_Heart, CARD_Liver, RCTD_Brain, RCTD_Heart, RCTD_Liver, cell2location_Brain, cell2location_Heart, and cell2location_Liver.

To evaluate performance, we conducted k-means clustering to create three clusters from the weight distribution data generated by SWIMR, designating them as brain organ, heart organ, and liver organ (Fig 4C). We then performed two-sided Wilcoxon rank-sum test to compare the proportion of each cell type within its associated region against the other regions. This allowed us to assess the accuracy of our cell type composition.

#### Human breast cancer 10x Visium data.

We conducted a deconvolution analysis on human breast cancer using 10x Visium data, employing references from various studies that highlight different characteristics (Note C in S1 Appendix). We implemented single-reference deconvolution methods on each reference and benchmarked our results against those from Liu et al., which was prioritized based on its highest allocation in the weight distribution.

#### Hepatocellular carcinoma 10x Visium data.

We applied SWIMR to hepatocellular carcinoma (HCC) data generated using 10x Visium and utilized three scRNA-seq references from distinct studies (Note C in S1 Appendix). To harmonize cell types across references, we mapped the originally annotated subtypes from each dataset to broader, generalized categories. Specifically, for the Sharma et al. dataset, CD8, CD4, and NK cells were grouped under T/NK cells. For the Ma et al. dataset, CAFs were mapped to fibroblasts, malignant cells to hepatocytes, TAMs to myeloid cells, TECs to endothelial cells, and T cells to T/NK cells, with other cell types remaining unchanged.

For benchmarking, we compared SWIMR against single-reference deconvolution approaches using the scRNA-seq reference from Ma et al., which was assigned the highest weight by our method (Note C in S1 Appendix). To compare the similarity between Ma et al. and Lu et al., we extracted the average of each cell type specific gene expression and calculated the spearman correlation between the two references to better understand the similar performance of cell type composition obtained by SWIMR and other single-reference-methods.

### Cell type specific spatially variable genes (ct-SVGs) detection

We employed CELINA [45] to detect cell-type specific spatially variable genes (ct-SVGs) in each spatial transcriptomics dataset. CELINA utilizes a spatially varying coefficient model to accurately capture the spatial expression patterns of genes in relation to the distribution of cell types across tissue locations. We followed the procedure provided and the default setting for filtering steps. Since CELINA filtered out those minor cell types, we also focused on those cell types remaining. When applying CELINA on SWIMR result, we applied single reference with the calculated cell type proportion one at a time and union the final selected ct-SVGs.

### Gene set enrichment analysis (GSEA)

We conducted a cell type specific Gene Set Enrichment Analysis (GSEA) on all datasets. After we performed CELINA to obtain the ct-SVGs, we picked the unique ct-SVGs from each method and performed GSEA analysis performed using the *g:GOSt* function in the *gProfiler2* package [112]. Specifically, this analysis utilized a custom-defined organism, “gp__7ie1_G7XX_XJw,” based on the Hallmark gene set (H) from the Molecular Signatures Database (MSigDB) [113] and we picked the significant hallmark at an FDR threshold of 0.05.

## Supporting information

S1 Appendix Supplementary figures. Fig A. UMAP plot of the two distinct scRNA-seq references from Zeisel et al.[1] (SRP135960) and Mizrak et al [2]. (GSE109447). Fig B. Weight distribution from SWIMR from simulation. Fig C. Scatter pie plot on the cell type compositions for scenarios that two references shared all cell types. Fig D. Scatter pie plot on the cell type compositions for scenario where two references shared no common cell types. Fig E. Cell-type-specific expression correlation between references for human heart data. Fig F. Scatter pie plot on the cell type compositions for human developing heart. Fig G. Similarity (Pearson correlation) between ST cell type proportion and scRNA-seq references in human lung cancer data. Fig H. Scatter pie plot on the cell type compositions for lung cancer inferred by SWIMR. Fig I. Scatter pie plot on the cell type compositions for lung cancer inferred by CARD. Fig J. Scatter pie plot on the cell type compositions for lung cancer inferred by RCTD. Fig K. Scatter pie plot on the cell type compositions for lung cancer inferred by cell2location. Fig L. Scatter pie plot of cell type compositions in the neural region subset of the mouse embryo, inferred by SWIMR. Fig M. Scatter pie plot of cell type compositions in the neural region subset of the mouse embryo inferred by CARD. Fig N. Scatter pie plot of cell type compositions in the neural region subset of the mouse embryo inferred by RCTD. Fig O. Scatter pie plot on the cell type compositions for mouse embryo brain tissue inferred by cell2location. Fig P. Scatter pie plot of cell type compositions in the visceral region of the mouse embryo, inferred by SWIMR. Fig Q. Scatter pie plot of cell type compositions in the visceral region of the mouse embryo inferred by CARD. Fig R. Scatter pie plot of cell type compositions in the visceral region of the mouse embryo inferred by RCTD. Fig S. Scatter pie plot of cell type compositions in the visceral region of the mouse embryo inferred by cell2location. Fig T. Scatter pie plot of cell type compositions in the cardiac region subset of the mouse embryo, inferred by SWIMR. Fig U. Scatter pie plot of cell type compositions in the cardiac region subset of the mouse embryo inferred by CARD. Fig V. Scatter pie plot of cell type compositions in the cardiac region subset of the mouse embryo inferred by RCTD. Fig W. Scatter pie plot of cell type compositions in the cardiac region subset of the mouse embryo inferred by cell2location. Fig X. Scatter pie plot on the cell type compositions for human breast cancer inferred by SWIMR. Fig Y. Scatter pie plot on the cell type compositions for human breast cancer inferred by CARD. Fig Z. Scatter pie plot on the cell type compositions for human breast cancer inferred by RCTD. Fig AA. Scatter pie plot on the cell type compositions for human breast cancer inferred by cell2location. Fig AB. Scatter pie plot on the cell type compositions for hepatocellular carcinoma inferred by SWIMR. Fig AC. Scatter pie plot on the cell type compositions for hepatocellular carcinoma inferred by CARD. Fig AD. Scatter pie plot on the cell type compositions for hepatocellular carcinoma inferred by RCTD. Fig AE. Scatter pie plot on the cell type compositions for hepatocellular carcinoma inferred by cell2location. Fig AF. Computation time and peak memory usage for each deconvolution method across three spatial transcriptomics datasets. Fig AG. Root mean squared error (RMSE) of cell type proportion estimates for all methods and simulation scenarios including the consensus ensemble of single-reference based methods. Fig AH. RMSE comparison for all deconvolution methods including the consensus ensemble of single-reference based methods for developing human heart. Fig AI. Subtype-specific reference analysis in breast cancer. Fig AJ. Sensitivity analysis of spatial parameters. **Supplementary Tables.** Table A. Detailed cell type information in the scRNA-seq datasets. Table B. Enriched pathway and corresponding p-values using ct-SVGs from lung cancer. Table C. Enriched pathway and corresponding p values using ct-SVGs from breast cancer. Table D. Enriched pathway and corresponding p values using ct-SVGs from hepatocellular carcinoma. Table E. Computation time and peak memory usage for each deconvolution method across three spatial transcriptomics datasets. **Supplementary Notes.** Note A. Methodological details of SWIMR. Note B. Simulation setting. Note C. Details on preprocessing Spatial Transcriptomics and Single Cell RNA-seq. Note D. Comparison with a consensus ensemble approach. Note E. Subtype-specific breast cancer reference analysis. Note F. Sensitivity analysis of separate spatial parameters for the cell-type proportion and reference-weight layers.
(DOCX)
