# Supplementary material for: Enhancing cell type deconvolution in spatial transcriptomics through incorporating multiple reference panels
Source: PLoS Genet. 2026 Nov 2;22(11):e1012345. doi: 10.1371/journal.pgen.1012345 (PMC13726986; doi:10.1371/journal.pgen.1012345)
Supplement: Supplementary file 1 [file pgen.1012345.s002.pdf]

This manuscript introduces SWIMR, a multi-reference spatial deconvolution framework that integrates multiple scRNA-seq references while modeling spatial smoothness via a two-level Conditional Autoregressive (CAR) structure. The method extends NMF-style deconvolution by allowing both cell-type proportions and reference weights to vary across tissue locations. Comprehensive simulations and multiple real datasets are presented, with empirical results suggesting improved performance over existing single-reference methods.

Overall, the paper addresses an important challenge in spatial transcriptomics: integrating multiple partially matched references in a spatially coherent and interpretable way. The methodological idea is promising and potentially impactful. However, several aspects of the modeling formulation and, in particular, the reference-weight mechanism require clarification to ensure statistical rigor, identifiability, and interpretability.

Major Comments

1. Concerns on identifiability between reference weights and cell-type proportions

The core model is given as:

$$Y = \sum_r S_r P_r^T Q_r + E, \tag{1}$$

where both the reference-specific proportions P_r and the reference weight matrices Q_r are estimated. Because the model involves products of P_r and Q_r, there is a potential **scaling non-identifiability** issue (e.g., scaling P_r up while scaling Q_r down may yield similar fits), unless additional constraints are imposed. With this concern, please clarify the following points:

- What constraints are imposed to ensure identifiability between P_r and Q_r?
- Are the reference weights normalized across references at each location (e.g., $\sum_r Z_{ir} = 1$ where Z_{ir} is the diagonal element of Q_r)?
- If no explicit normalization is enforced, how should Q_r be interpreted quantitatively and compared across references?

Please explicitly state any simplex/normalization constraints (if used), or provide a short discussion of identifiability and how the optimization avoids degenerate solutions.

2. Concerns about the interpretation and normalization of reference weights

The manuscript refers to Q_r (or its diagonal elements) as reference weights and uses them to interpret which reference contributes most in a region. However, the current exposition does not make fully clear whether the weights are intended to represent: 1) proportions that sum to 1 across references at each location, or 2) unconstrained nonnegative scaling factors. This distinction matters for interpretability and for claims such as "the model places nearly all weight on the internal reference".

With these concerns, please clarify the following points:

- Are weights comparable across references and across locations?
- Do weights sum to 1 per location, or are they rescaled post hoc for reporting?

Please consider adding one sentence that precisely defines the weight scale (and any normalization) as well as how weights are summarized in figures.

3. Concerns on shared spatial autocorrelation parameter for two distinct spatial processes

The method applies CAR modeling to both: 1) cell-type proportions P_r, and 2) reference weights (diagonal elements of Q_r), apparently using the same spatial kernel/adjacency matrix K and the same spatial autocorrelation parameter ϕ. This implies that spatial smoothness of cell composition and spatial smoothness of reference relevance follow the same spatial dependence structure and strength, which may be a strong assumption. Reference relevance could plausibly vary more sharply (or differently) than cell-type proportions.

With these concerns, please clarify the following points:

- Please justify why the same ϕ (and/or the same K) is appropriate for both layers.
- Consider allowing separate spatial parameters (e.g., ϕ_P and ϕ_Q) or discuss this as a limitation/future extension.

4. Concerns on Gaussian noise assumption for count-based ST data

The model assumes a Gaussian error term E (e.g., $E_{gn} \sim \mathcal{N}(0, \sigma^2)$). Since ST platforms often produce count data with overdispersion and sparsity, it would be helpful to justify this assumption. So please clarify the following points:

- Is Y modeled on raw counts, normalized counts, or transformed values (e.g., log-normalized)?
- If counts are used, why is Gaussian noise appropriate versus a count model (Poisson/Negative Binomial)?

A brief discussion acknowledging this modeling choice and its implications would strengthen the method.

5. Concerns on mechanism of reference "competition" vs. additive contribution

The formulation is additive across references, but it is not fully clear whether and how references *compete* such that a better-matched reference is emphasized while others are down-weighted. Empirically, SWIMR appears to prioritize contextually relevant references in simulations and real data, but the mechanism could be described more explicitly. Please clarify the followig points:

- Does the optimization induce competition through normalization, constraints, or regularization?
- How does the model avoid uniform weighting in mismatched scenarios?

Minor Comments

1. The Methods mention maximum likelihood optimization, but the likelihood/objective function is not written explicitly. Including it (even briefly) would improve reproducibility and clarity.

2. Consider adding runtime and memory benchmarks comparing SWIMR to baseline methods, especially since scalability is a practical concern and SCDC is excluded in real data due to computational burden.
