# Supplementary material for: Enhancing cell type deconvolution in spatial transcriptomics through incorporating multiple reference panels
Source: PLoS Genet. 2026 Nov 2;22(11):e1012345. doi: 10.1371/journal.pgen.1012345 (PMC13726986; doi:10.1371/journal.pgen.1012345)
Supplement: Supplementary file 2 [file pgen.1012345.s004.pdf]

The authors have substantially addressed my previous comments, and the manuscript has improved considerably. I have a few minor comments for the authors to consider.

1. Equation (4) appears to contain an error. The CAR model for the reference weights is currently written as

   $$Z_{ir} = q_r + \varphi \sum_{j \neq i} K_{ij} (Z_{ir} - q_r) + \varepsilon_{ir}.$$

   Because the text states that the reference weight at location i is modeled using neighboring locations, the term inside the summation should presumably involve Z_{jr} rather than Z_{ir}. Please verify and correct the equation accordingly.

2. Please correct notation inconsistencies in the spatial sensitivity analysis. For example, the response refers to Φ_P and Φ_Q, whereas the manuscript primarily uses K and φ. In addition, the statement that "Φ_P was increased to 50 times Φ_P" appears to be a typo and likely should refer to $\frac{\Phi_Q}{\Phi_P} = 50.$
3. Minor consistency corrections are needed. In the Methods, the residuals are described as independently and identically distributed, but the variance is written as σ_{gn}^2, which implies gene/location-specific variances. Please either use a common variance or remove "identically." Also, please check the Supplementary Figure numbering for the runtime/memory benchmark, which is referred to as Supplementary Figure 32 in some places and Supplementary Figure 34 in others.

Apart from these minor issues, I consider my previous concerns to have been satisfactorily addressed.
