# Supplementary material for: Enhancing cell type deconvolution in spatial transcriptomics through incorporating multiple reference panels
Source: PLoS Genet. 2026 Nov 2;22(11):e1012345. doi: 10.1371/journal.pgen.1012345 (PMC13726986; doi:10.1371/journal.pgen.1012345)
Supplement: Supplementary file 3 [file pgen.1012345.s001.docx]

**Supplementary Information**

**Robust Cell Type Deconvolution for Spatial Transcriptomics via Spatially Weighted Multi-Reference Modeling**

# Peiyao Zhao^1, 2,^ Justin A. Colacino^3, 4,^ Ying Ma^5, 6, *^ and Xiang Zhou^7, *^

^1^Department of Biostatistics, School of Public Health, University of Michigan, Ann Arbor, MI, USA

^2^Center for Statistical Genetics, University of Michigan, Ann Arbor, MI, USA

^3^Department of Environmental Health Sciences, University of Michigan School of Public Health, Ann Arbor, MI, USA

^4^Department of Nutritional Sciences, University of Michigan School of Public Health,

Ann Arbor, MI, USA

^5^Department of Biostatistics, School of Public Health, Brown University, Providence, RI, USA

^6^Center for Computational Molecular Biology, Brown University, Providence, RI, USA

^7^Department of Statistics and Data Science, Yale University, New Haven, CT, USA

^*^Correspondence to Ying Ma ([ying_ma@brown.edu](mailto:ying_ma@brown.edu)) and Xiang Zhou (xiang.zhou.xz735@yale.edu)

Table of Content

[**Supplementary Figures**	3](#_Toc242993334)

[**Supplementary Tables**	39](#_Toc242993335)

[**Supplementary Notes**	48](#_Toc242993336)

[**Note A. Methodological details of SWIMR**	48](#_Toc242993337)

[**Note B. Simulation setting**	48](#_Toc242993338)

[**Note C. Details on preprocessing Spatial Transcriptomics and Single Cell RNA-seq**	48](#_Toc242993339)

[**Note D. Comparison with a consensus ensemble approach**	48](#_Toc242993340)

[**Note E. Subtype-specific breast cancer reference analysis**	48](#_Toc242993341)

[**Note F. Sensitivity analysis of separate spatial parameters for the cell-type proportion and reference-weight layers**	48](#_Toc242993342)

## **Supplementary Figures**

**Fig A. UMAP plot of the two distinct scRNA-seq reference datasets from Zeisel et al.^1^ (SRP135960) and Mizrak et al.^2^ (GSE109447)**

**Fig B. Weight distribution from SWIMR from simulation.** Here, we present a representative example generated using one random seed (seed = 1), with results from other seeds showing similar patterns.

**Fig C. Scatter pie plot on the cell type compositions for scenarios that two references shared all cell types.** Here, we present a representative example generated using one random seed (seed = 1), with results from other seeds showing similar patterns. Note that in the result of RCTD_ref2, ependymal cells were removed in the deconvolution process due to its minority

**Fig D. Scatter pie plot on the cell type compositions for scenario where two references shared no common cell types.** Here, we present a representative example generated using one random seed (seed = 1), with results from other seeds showing similar patterns. Note that under this scenario, all the results generated from single-reference base deconvolution methods only contain part if the cell types and hence no color overlapping.

**Fig E. Cell-type-specific expression correlation between references for human heart data.**

**Fig F. Scatter pie plot on the cell type compositions for human developing heart**

**Fig G. Similarity (Pearson correlation) between ST cell type proportion and scRNA-seq references in human lung cancer data**.

**Fig H. Scatter pie plot on the cell type compositions for lung cancer inferred by SWIMR.** Here, for each cell type, the cell type proportion was scaled to 0-1 range. Color was shown to represent the 0-1 range of cell type proportions correspondingly.

**Fig I. Scatter pie plot on the cell type compositions for lung cancer inferred by CARD.** Here, for each cell type, the cell type proportion was scaled to 0-1 range. Color was shown to represent the 0-1 range of cell type proportions correspondingly.

**Fig J. Scatter pie plot on the cell type compositions for lung cancer inferred by RCTD.** Here, for each cell type, the cell type proportion was scaled to 0-1 range. Color was shown to represent the 0-1 range of cell type proportions correspondingly. Some cell types were filtered in RCTD result due to minority.

**Fig K. Scatter pie plot on the cell type compositions for lung cancer inferred by cell2location.** Here, for each cell type, the cell type proportion was scaled to 0-1 range. Color was shown to represent the 0-1 range of cell type proportions correspondingly.

**Fig L.** **Scatter pie plot of cell type compositions in the neural region subset of the mouse embryo, inferred by SWIMR.** Here, for each cell type, the cell type proportion was scaled to 0-1 range. Color was shown to represent the 0-1 range of cell type proportions correspondingly. Note that separated results from SWIMR into neural, cardiac and visceral region **only for visualization purposes**, the result itself was calculated together

**Fig M. Scatter pie plot of cell type compositions in the neural region subset of the mouse embryo inferred by CARD.** Here, for each cell type, the cell type proportion was scaled to 0-1 range. Color was shown to represent the 0-1 range of cell type proportions correspondingly.

**Fig N. Scatter pie plot of cell type compositions in the neural region subset of the mouse embryo inferred by RCTD.** Here, for each cell type, the cell type proportion was scaled to 0-1 range. Color was shown to represent the 0-1 range of cell type proportions correspondingly.

**Fig O. Scatter pie plot on the cell type compositions for mouse embryo brain tissue inferred by cell2location.** Here, for each cell type, the cell type proportion was scaled to 0-1 range. Color was shown to represent the 0-1 range of cell type proportions correspondingly.

**Fig P. Scatter pie plot of cell type compositions in the visceral** **region of the mouse embryo, inferred by SWIMR.** Here, for each cell type, the cell type proportion was scaled to 0-1 range. Color was shown to represent the 0-1 range of cell type proportions correspondingly. Note that separated results from SWIMR into neural, cardiac and visceral region **only for visualization purposes**, the result itself was calculated together.

**Fig Q. Scatter pie plot of cell type compositions in the visceral** **region of the mouse embryo inferred by CARD.** Here, for each cell type, the cell type proportion was scaled to 0-1 range. Color was shown to represent the 0-1 range of cell type proportions correspondingly.

**Fig R. Scatter pie plot of cell type compositions in the visceral** **region of the mouse embryo inferred by RCTD.** Here, for each cell type, the cell type proportion was scaled to 0-1 range. Color was shown to represent the 0-1 range of cell type proportions correspondingly.

**Fig S. Scatter pie plot of cell type compositions in the visceral** **region of the mouse embryo inferred by cell2location.** Here, for each cell type, the cell type proportion was scaled to 0-1 range. Color was shown to represent the 0-1 range of cell type proportions correspondingly.

**Fig T. Scatter pie plot of cell type compositions in the cardiac region subset of the mouse embryo, inferred by SWIMR.** Here, for each cell type, the cell type proportion was scaled to 0-1 range. Color was shown to represent the 0-1 range of cell type proportions correspondingly. Note that separated results from SWIMR into neural, cardiac and visceral region **only for visualization purposes**, the result itself was calculated together.

**Fig U. Scatter pie plot of cell type compositions in the cardiac region subset of the mouse embryo inferred by CARD.** Here, for each cell type, the cell type proportion was scaled to 0-1 range. Color was shown to represent the 0-1 range of cell type proportions correspondingly.

**Fig V. Scatter pie plot of cell type compositions in the cardiac region subset of the mouse embryo inferred by RCTD.** Here, for each cell type, the cell type proportion was scaled to 0-1 range. Color was shown to represent the 0-1 range of cell type proportions correspondingly.

**Fig W. Scatter pie plot of cell type compositions in the cardiac region subset of the mouse embryo inferred by cell2location.** Here, for each cell type, the cell type proportion was scaled to 0-1 range. Color was shown to represent the 0-1 range of cell type proportions correspondingly.

**Fig X. Scatter pie plot on the cell type compositions for human breast cancer inferred by SWIMR.** Here, for each cell type, the cell type proportion was scaled to 0-1 range. Color was shown to represent the 0-1 range of cell type proportions correspondingly.

**Fig Y. Scatter pie plot on the cell type compositions for human breast cancer inferred by CARD.** Here, for each cell type, the cell type proportion was scaled to 0-1 range. Color was shown to represent the 0-1 range of cell type proportions correspondingly.

**Fig Z. Scatter pie plot on the cell type compositions for human breast cancer inferred by RCTD.** Here, for each cell type, the cell type proportion was scaled to 0-1 range. Color was shown to represent the 0-1 range of cell type proportions correspondingly.

**Fig AA. Scatter pie plot on the cell type compositions for human breast cancer inferred by cell2location.** Here, for each cell type, the cell type proportion was scaled to 0-1 range. Color was shown to represent the 0-1 range of cell type proportions correspondingly.

**Fig AB. Scatter pie plot on the cell type compositions for hepatocellular carcinoma inferred by SWIMR.** Here, for each cell type, the cell type proportion was scaled to 0-1 range. Color was shown to represent the 0-1 range of cell type proportions correspondingly.

**Fig AC. Scatter pie plot on the cell type compositions for hepatocellular carcinoma inferred by CARD.** Here, for each cell type, the cell type proportion was scaled to 0-1 range. Color was shown to represent the 0-1 range of cell type proportions correspondingly.

**Fig AD. Scatter pie plot on the cell type compositions for hepatocellular carcinoma inferred by RCTD.** Here, for each cell type, the cell type proportion was scaled to 0-1 range. Color was shown to represent the 0-1 range of cell type proportions correspondingly.

**Fig AE. Scatter pie plot on the cell type compositions for hepatocellular carcinoma inferred by cell2location.** Here, for each cell type, the cell type proportion was scaled to 0-1 range. Color was shown to represent the 0-1 range of cell type proportions correspondingly.

**Fig AF. Computation time and peak memory usage for each deconvolution method across three spatial transcriptomics datasets.** Computation time is shown in minutes in the top panel, and peak memory usage is shown in MB in the bottom panel. SWIMR, CARD, RCTD, and SCDC were run in R using a single CPU core on an Intel(R) Xeon(R) Platinum 8268 CPU @ 2.90GHz processor under Ubuntu 22.04.5 LTS. cell2location was run in Python using an NVIDIA H100 NVL GPU. For methods run separately across multiple references, runtimes were summed across reference-specific runs, while peak memory usage was reported as the maximum peak memory observed across runs because the runs were performed sequentially. For cell2location, the memory panel reports CPU peak memory increase to make it comparable with the R-based methods; GPU memory allocation was recorded separately and was not included in the main memory comparison because CPU RAM and GPU memory are distinct hardware resources. SCDC was not completed for the human breast cancer Visium dataset due to its long runtime, exceeding three days.

**Fig AG. Root mean squared error (RMSE) of cell type proportion estimates for all methods and simulation scenarios including the consensus ensemble of single-reference based methods.**

**Fig AH. RMSE comparison for all deconvolution methods including the consensus ensemble of single-reference based methods for developing human heart.**

**Fig AI. Subtype-specific reference analysis in breast cancer. A**. spatial distribution of subtype-specific reference weights. **B**. gene expression pattern of marker gene *ERBB2.* C: marker-gene correlations showing biological support for the Cancer LumB single-cell signal identified by SWIMR.

**Fig AJ. Sensitivity analysis of spatial parameters.** Cell-type proportion estimation accuracy across all simulation scenarios was measured by root mean squared error (RMSE), with the ratio of the reference-weight spatial parameter to the cell-type proportion spatial parameter $\Phi_{Q}/\Phi_{P}$ set to 0.5, 1, 10, 50, and 100. Here, $\Phi_{P}$ and $\Phi_{Q}$ denote the spatial parameters assigned to the cell-type proportion and reference-weight layers, respectively.

## **Supplementary Tables**

**Table A. Detailed cell type information in the scRNA-seq datasets.**

| scRNA-seq data | Cell type | Data acquisition | Paper |
| --- | --- | --- | --- |
| Mouse Olfactory scRNA-seq data | neurons (n=11,702), astrocytes (n=5,039), oligodendrocytes (n=1,397), vascular cells (n=1,162), immune cells (1,078), and ependymal cells (n = 15) | SRP135960 | Zeisel et al.^1^ |
| Mouse Olfactory scRNA-seq data | astrocytes (n = 13,765), endothelial (n = 1,774), ependymal (n=997), microglia (n=5,525), neuron (n = 3,110), oligodendrocytes (n = 7,513) | GSE109447 | Mizrak et al.^2^ |
| Human developing heart (ISS) | (0) Capillary endothelium (n=879), (1) Ventricular cardiomyocytes (n=1,468), (10) Endothelium/ pericytes/ adventia (n=955), (12) Cardiomyocytes (n=3,804), (14) Cells related to cardiac neural crest (n=108), (2) Fibroblast-like (AV mesenchyme related) (n=1,001), (3) Subepicardial cells) (n=467), (4) Fibroblast-like (coronary & mediastinal vasculature related) (n=517), (5) Smooth muscle cells/ fibroblast-like (n=967), (7) Atrial cardiomyocytes (n=5,577), (8) Fibroblast-like (outflow tract & valve related) (n=1,026), (9) Epicardial cells) (n=685) | https://github.com/JiawenChenn/St-review/tree/main/processed_data/heart | Chen et al.^3^ |
| Human developing heart | Atrial cardiomyocytes (n=156), Capillary endothelium (n=596), Cardiac neural crest cells & Schwann progenitor cells (n=75), Epicardial cells (n=106), Epicardium-derived cells (n=390), Fibroblast-like (related to larger vascular development) (n=150), Fibroblast-like (related to smaller vascular development) (n=342), Myoz2-enriched cardiomyocytes (n=97), Smooth muscle cells / fibroblast-like) (n=262), Ventricular cardiomyocytes (n=504) | 10X Genomics EGAS00001003996 | Chen et al.^3^ |
| Lung cancer (10x) | Alveolar cell type (n=1,160), Alveolar cell type (n=2,367), B cell (n=695), Ciliated (n=103), Club (n=25), DC mature (n=144), Endothelial cell (n=10,774), Macrophage (n=2,876), Macrophage alveolar (n=3,566), Mast cell (n=393), Monocyte (n=516), NK cell (n=1,514), Neutrophils (n=8), Plasma cell (n=421), Stromal (n=326), T cell CD4 (n=1,247), T cell CD8 (n=2,006), T cell regulatory (n=354), Tumor cells (n=2,251), cDC1 (n=195), cDC2 (n=1501), other (n=324), pDC (n=198) and transitional club/AT2 (n=799) | https://zenodo.org/records/7227571 | Salcher et al.^4^ |
| Lung cancer (BD-Rhapsody) | Alveolar cell type 1 (n=21), Alveolar cell type 2 (n=69), B cell (n=146), Ciliated (n=61), Club (n=13), DC mature (n=6), Endothelial cell (n=46), Macrophage (n=482), Macrophage alveolar (n=1,068), Mast cell (n=79), Monocyte (n=510), NK cell (n=810), Neutrophils (n=3,592), Plasma cell (n=187), Stromal (n=62), T cell CD4 (n=696), T cell CD8 (n=1,103), T cell regulatory (n=261), Tumor cells (n=2,131), cDC1 (n=16), cDC2 (n=170), other (n=128), pDC (n=37) and transitional club/AT2 (n=41) | https://zenodo.org/records/7227571 | Salcher et al.^4^ |
| Lung cancer (inDrop) | Alveolar cell type 1 (n=1), Alveolar cell type 2 (n=37), B cell (n=235), Ciliated (n=17), Club (n=4), DC mature (n=11), Endothelial cell (n=45), Macrophage (n=658), Macrophage alveolar (n=324), Mast cell (n=123), Monocyte (n=539), NK cell (n=106), Neutrophils (n=277), Plasma cell (n=270), Stromal (n=170), T cell CD4 (n=718), T cell CD8 (n=582), T cell regulatory (n=292), Tumor cells (n=913), cDC1 (n=11), cDC2 (n=198), other (n=109), pDC (n=85) and transitional club/AT2 (n=26) | https://zenodo.org/records/7227571 | Salcher et al.^4^ |
| Lung cancer (Singleron) | Alveolar cell type 2 (n=58), B cell (n=207), Ciliated (n=776), Club (n=239),DC mature (n=57), Endothelial cell (n=584), Macrophage (n=4,252), Macrophage alveolar (n=285), Mast cell (n=245), Monocyte (n=1,042), NK cell (n=134), Neutrophils (n=1,076), Plasma cell (n=1,486), Stromal (n=2,610), T cell CD4 (n=406), T cell CD8 (n=720), T cell regulatory (n=275), Tumor cells (n=25,464), cDC1 (n=19), cDC2 (n=230), other (n=831), pDC (n=53) and transitional club/AT2 (n=71) | https://zenodo.org/records/7227571 | Salcher et al.^4^ |
| Lung cancer (Smart-seq2) | Macrophage (n=1), Macrophage alveolar (n=1), NK cell (n=41), T cell CD4 (n=825), T cell CD8 (n=796), T cell regulatory (n=175) and other (n=76) | https://zenodo.org/records/7227571 | Salcher et al.^4^ |
| Mouse Brain | Ependymal cell (n=71), Erythroid cell_Hbb-bh1 high (n=159), Erythroid cell_Hbb-bt high (n=95), Glial cell_Fabp7 high (n=332), Macrophage (n=66), Microglia (n=79), Neuron_Calb1 high (n=76), Neuron_Gadd45g high (n=325), Neuron_Gap43&Stmn4 high (n=663), Neuron_Lhx9 high (n=207), Neuron_Mapt high (n=556), Neuron_Neurod6 high (n=256), Neuron_Neurod6&Neurod2 high (n=348), Neuron_Tal2 high (n=228), Proliferating cell_Kpna2 high (n=259), Proliferating cell_Ube2c high (n=292), Radial glia_Vim high (n=290) and Stromal cell (n=97) | https://bis.zju.edu.cn/MCA/ | Fei et al. ^5^ |
| Mouse Liver | Erythroid cell (n=83), Erythroid progenitor (n=1763), Hepatocyte (n=52), Kupffer cell (n=76), Macrophage cell (n=154), Mast cell_Mcpt8 high (n=42), Megakaryocyte (n=23), Neutrophil_Elane high (n=249), Neutrophil_Ngp high (n=61), Pre B cell (n=22) and Stem cell_H2afy high (n=174) | https://bis.zju.edu.cn/MCA/ | Fei et al. ^5^ |
| Mouse Heart | Atrial cardiomyocytes (ACM)_Myl7 high (n=448), Capillary endothelial cell_Gpihbp1 high (n=159), Dendritic cell (n=71), Distal progenitor cell (n=332), Endocardial cell (n=490), Endothelial cell_Aqp1 high (n=280), Epithelial cell _Ccl25 high (n=343), Epithelial cell_Pth high (n=29), Erythroid cell_Hba-x high (n=180), Erythroid cell_Hbb-bt high (n=226), Lymphocyte_Ccl4 high (n=62), Lymphocyte_Trbc2 high (n=866), Macrophage (m=648), Macrophage_Lyz2 high (n=281), Mast cell (n=50), Mesothelial cell (n=689), Neutrophil (n=58), Stromal cell_Dlk1 high (n=360), Stromal cell_Eln high (n=302), Stromal cell_Id high (n=1269), Stromal cell_Tcf21 high (n=912), T cell_Ccl5 high (n=172), T cell (n=666), T cell_Gzma high (n=427) and Ventricular cardiomyocytes (VCM)_Myl2 high (n=813) | https://bis.zju.edu.cn/MCA/ | Fei et al. ^5^ |
| Human breast cancer (Wu et al.) | B cells (n=5,705), Endothelial cells Epithelial cells (n=5,929), malignant cells (n=21,521), Myeloid (4630), Stromal (n=8,123) and T cells(n=16,614) | https://doi.org/10.6084/m9.figshare.25019903 | Pang et al.^6^ |
| Human breast cancer (Xu et al.) | CAFs (n=5,373), Endothelial cells (n=928), Epithelial cells (n=5,089), malignant (n=7,846), Smooth muscle cells (n=245) and T cells (n=3,295) | https://doi.org/10.6084/m9.figshare.25019903 | Pang et al.^6^ |
| Human breast cancer (Bhupinder et al.) | B cells (n=5,837), Endothelial cells (n=2,342), Epithelial cells (n= 50,743), malignant (n=28,615), Myeloid (n=17,554), plasmablasts (n=6,039), Stromal (n=12,787) and T cells (n=40,968) | https://doi.org/10.6084/m9.figshare.25019903 | Pang et al.^6^ |
| Human breast cancer (Liu et al.) | B cells (n=2,326), CAFs (n=2,104), Endothelial cells (n=2,687), Epithelial cells (n=10,295), malignant (n=4,429), Myeloid (n=2,006), plasmablasts (n=2,119) and T cells (n=19,753) | https://doi.org/10.6084/m9.figshare.25019903 | Pang et al.^6^ |
| Hepatocellular carcinoma (Sharma et al.) | B Cells (n=1,376), Bi-Potent Cells(n=336) Endothelial Cells(n=11,207), Fibroblasts(n=1,891), Hepatocytes (n=12,646), Mast Cells (n=119), Myeloid (n=6726), T/NK Cells (n=36,422) and Tregs (n=2,866) | GSE156625 | Sharma et al.^7^ |
| Hepatocellular carcinoma (Ma et al.) | B Cells (n=2,291), Endothelial Cells (n=2,646), Fibroblasts (n=1,459), Hepatocytes (n=17,164), Myeloid (n=5,171), T/NK Cells (n=19,587) and unclassified (n=8,403) | GSE151530 | Ma et al.^8^ |
| Hepatocellular carcinoma (Lu et al.) | B Cells (n=3,685), Endothelial Cells (n=3,644), Fibroblasts (n=2,266), Hepatocytes (n=20,782), Myeloid (n=15,947) and T/NK Cells (n=25,591) | GSE149614 | Lu et al.^9^ |

**Table B. Enriched pathway and corresponding p-values using ct-SVGs from lung cancer****.**

GSEA result from ct-SVGs uniquely detected using tumor cells proportion inferred by SWIMR

| Term | P_value |
| --- | --- |
| HALLMARK_EPITHELIAL_MESENCHYMAL_TRANSITION | 9.136569e-09 |
| HALLMARK_TNFA_SIGNALING_VIA_NFKB | 2.754174e-05 |
| HALLMARK_ANDROGEN_RESPONSE | 6.658720e-04 |
| HALLMARK_TGF_BETA_SIGNALING | 1.323081e-03 |
| HALLMARK_MITOTIC_SPINDLE | 1.482943e-03 |
| HALLMARK_APOPTOSIS | 4.552043e-03 |

GSEA result from ct-SVGs uniquely detected using other cells proportion inferred by SWIMR

| Term | P_value |
| --- | --- |
| HALLMARK_TNFA_SIGNALING_VIA_NFKB | 8.545993e-05 |
| HALLMARK_ANDROGEN_RESPONSE | 1.090482e-04 |
| HALLMARK_MTORC1_SIGNALING | 1.933324e-04 |
| HALLMARK_P53_PATHWAY | 4.258662e-04 |
| HALLMARK_PROTEIN_SECRETION | 2.757913e-03 |
| HALLMARK_REACTIVE_OXYGEN_SPECIES_PATHWAY | 7.396063e-03 |
| HALLMARK_UV_RESPONSE_UP | 8.383537e-03 |
| HALLMARK_EPITHELIAL_MESENCHYMAL_TRANSITION | 1.469392e-02 |
| HALLMARK_APOPTOSIS | 2.518161e-02 |
| HALLMARK_HYPOXIA | 2.745557e-02 |

**Table C. Enriched pathway and corresponding p values using ct-SVGs from breast cancer.**

GSEA result from ct-SVGs uniquely detected using myeloid cells proportion inferred by SWIMR

| Term | P_value |
| --- | --- |
| HALLMARK_MYC_TARGETS_V1 | 0.0001276948 |
| HALLMARK_OXIDATIVE_PHOSPHORYLATION | 0.0003410583 |
| HALLMARK_PI3K_AKT_MTOR_SIGNALING | 0.0109526915 |
| HALLMARK_MTORC1_SIGNALING | 0.0256077065 |
| HALLMARK_INTERFERON_ALPHA_RESPONSE | 0.0296750571 |

**Table D. Enriched pathway and corresponding p values using ct-SVGs from hepatocellular carcinoma.**

GSEA result from ct-SVGs uniquely detected using fibroblast cells proportion inferred by SWIMR

| Term | P_value |
| --- | --- |
| HALLMARK_INTERFERON_GAMMA_RESPONSE | 0.0001116536 |
| HALLMARK_OXIDATIVE_PHOSPHORYLATION | 0.0001116536 |
| HALLMARK_REACTIVE_OXYGEN_SPECIES_PATHWAY | 0.0026752507 |
| HALLMARK_COMPLEMENT | 0.0034218576 |
| HALLMARK_TNFA_SIGNALING_VIA_NFKB | 0.0095684470 |
| HALLMARK_MTORC1_SIGNALING | 0.0252180156 |

GSEA result from ct-SVGs uniquely detected using B cells proportion inferred by SWIMR

| Term | P_value |
| --- | --- |
| HALLMARK_MYC_TARGETS_V1 | 1.421966e-06 |
| HALLMARK_INTERFERON_GAMMA_RESPONSE | 8.501776e-03 |
| HALLMARK_ALLOGRAFT_REJECTION | 0.03524391 |
| INTERFERON_ALPHA_RESPONSE | 0.04271164 |

**Table E. Computation time and peak memory usage for each deconvolution method across three spatial transcriptomics datasets.** SWIMR, CARD, RCTD, and SCDC were run in R using a single CPU core on an Intel(R) Xeon(R) Platinum 8268 CPU @ 2.90GHz processor under Ubuntu 22.04.5 LTS. cell2location was run in Python using an NVIDIA H100 NVL GPU. For methods run separately across multiple references, runtimes were summed across reference-specific runs, while peak memory usage was reported as the maximum peak memory observed across runs because the runs were performed sequentially. For cell2location, the memory panel reports CPU peak memory increase to make it comparable with the R-based methods; GPU memory allocation was recorded separately and was not included in the main memory comparison because CPU RAM and GPU memory are distinct hardware resources. SCDC was not completed for the human breast cancer Visium dataset due to its long runtime, exceeding three days.

“+” indicates that the reported memory value for cell2location represents CPU peak memory increase only; GPU memory allocation was recorded separately and was not included in the table.

|  | Method | Human heart (ISS) | Mouse olfactory bulb (ST) | Human breast cancer (Visium) |
| --- | --- | --- | --- | --- |
| Computation Time (Seconds) | SWIMR | 9.995 | 65.091 | 739.672 |
|  | CARD | 5.702 | 32.274 | 498.556 |
|  | RCTD | 214.266 | 395.471 | 1809.571 |
|  | SCDC | 79.425 | 5234.751 | Not completed |
|  | cell2location | 10149.07211 | 17685.14742 | 27471.84357 |
| Peak Memory Usage (MB) | SWIMR | 100.6 | 12514.9 | 37787 |
|  | CARD | 141.7 | 12544.7 | 31944.7 |
|  | RCTD | 720.3 | 12515 | 16436.1 |
|  | SCDC | 130.8 | 17946.2 | Not completed |
|  | cell2location | 1715.820312 + | 11378.11719 + | 7297.597656 + |

## **Supplementary Notes**

## **Note A. Methodological details of SWIMR**

**The Gaussian kernel function**

Following the previous studies^10,11^, we set the Gaussian kernel as our spatial kernel in the CAR model, which is

$$K\left( \boldsymbol{a}_{i},\boldsymbol{a}_{j} \right)=exp(-\frac{\left| \left| a_{i}-a_{j} \right| \right|^{2}}{2\sigma^{2}})$$

Where we utilized the Euclidian distance and we simply fixed the bandwidth to be 0.1

**Statistical inference for SWIMR**

We set our notations as follows:

$G$: Number of genes used for deconvolution, defined as the union of genes appearing in any reference basis matrix $\boldsymbol{S}_{r}$, allowing the model to capture comprehensive gene information across multiple references.

$N$: Number of spatial locations of the spatial transcriptomics dataset

$\boldsymbol{Y}\in R^{G \times N}:$The observed spatial transcriptomics dataset

$\boldsymbol{Y}_{r}\in R^{G \times N}:$expected mean of spatial transcriptomics dataset coming from reference $r$

$\boldsymbol{S}_{r}\in R^{G \times C_{r}}:$reference basis matrix from reference $r$, $C_{r}$ represents number of cell types in reference $r$ obtained following the previous literature^12^

$\boldsymbol{P}_{r}\in R^{G \times C_{r}}:$ cell type proportion estimated based on basis reference matrix $\boldsymbol{S}_{r}$

$\boldsymbol{Q}_{r}\in R^{N \times N}:$ a diagonal matrix representing the weight for each spatial location contributing from reference $\boldsymbol{S}_{r}$

We posit that the observed data matrix is a weighted sum of $\boldsymbol{Y}_{r}$ s, each estimated from different references. Following the CARD framework^12^, $\boldsymbol{Y}_{r}$is estimated using the reference basis $\boldsymbol{S}_{r}$, which is derived from the r-th scRNA-seq reference.

$$\boldsymbol{Y}=\sum_{r} \boldsymbol{Y}_{r}\boldsymbol{Q}_{r}$$

$$\boldsymbol{Y}_{r}=\boldsymbol{S}_{r}\boldsymbol{P}_{r}^{T}+\tilde{\boldsymbol{E}_{r}}$$

In this way we get

$$\boldsymbol{Y}=\sum_{r} (\boldsymbol{S}_{r}\boldsymbol{P}_{r}^{T}\boldsymbol{Q}_{r}+\tilde{\boldsymbol{E}_{r}})$$

We denoted $\boldsymbol{E}=\sum_{r} \tilde{\boldsymbol{E}_{r}}$, then we have the equation (1) in the main text showing that

$$\boldsymbol{Y}=\sum_{r} \boldsymbol{S}_{r}\boldsymbol{P}_{r}^{T}\boldsymbol{Q}_{r}+\boldsymbol{E}$$

$\boldsymbol{P}_{r_{k}}$ and $\boldsymbol{Z}_{r}=diag(\boldsymbol{Q}_{r})$ can be constructed based on CARD framework:

$$\boldsymbol{P}_{r_{k}}\boldsymbol{\sim MVN}\left( \mathbf{s}_{r_{k}}\mathbf{1}_{N}\mathbf{,}\boldsymbol{\Sigma}_{r_{k}}^{\boldsymbol{(P)}} \right)$$

$$\boldsymbol{Z}_{r}\boldsymbol{\sim MVN}\left( \mathbf{q}_{r}\boldsymbol{1}_{N}\mathbf{,}\boldsymbol{\Sigma}_{r}^{\boldsymbol{(Q)}} \right)$$

Where covariance matrix $\boldsymbol{\Sigma}_{r_{k}}^{\boldsymbol{(P)}}$ can be calculated as

$$\boldsymbol{\Sigma}_{r_{k}}^{\boldsymbol{(}\boldsymbol{P}\boldsymbol{)}}\mathbf{=}\left( \boldsymbol{I}_{\boldsymbol{n}}\mathbf{-}\phi\tilde{\boldsymbol{K}} \right)^{\mathbf{-1}}\boldsymbol{M}_{r_{k}}$$

$$\boldsymbol{=}\left( \boldsymbol{I}_{\boldsymbol{n}}\mathbf{-}\phi\tilde{\boldsymbol{K}} \right)^{\mathbf{-1}}\lambda_{r_{k}}\boldsymbol{D}^{\mathbf{-1}}$$

$$\boldsymbol{=}\lambda_{r_{k}}\left( \boldsymbol{D}\mathbf{-}\phi\boldsymbol{D}\tilde{\boldsymbol{K}} \right)^{\mathbf{-1}}$$

$$\boldsymbol{=}\lambda_{r_{k}}\left( \boldsymbol{D}\mathbf{-}\phi\boldsymbol{K} \right)^{\mathbf{-1}}$$

Where $\boldsymbol{K}$ is the Gaussian kernel constructed by spatial coordinates; $\boldsymbol{D}$ is the diagonal column sum of $\boldsymbol{K}$.

And covariance matrix $\boldsymbol{\Sigma}_{r}^{\boldsymbol{(Q)}}$ can be calculated as

$$\boldsymbol{\Sigma}_{r}^{\boldsymbol{(Q)}}\mathbf{=}\left( \boldsymbol{I}_{\boldsymbol{n}}\mathbf{-}\phi\tilde{\boldsymbol{K}} \right)^{\mathbf{-1}}\boldsymbol{M}_{r}$$

$$\boldsymbol{=}\left( \boldsymbol{I}_{\boldsymbol{n}}\mathbf{-}\phi\tilde{\boldsymbol{K}} \right)^{\mathbf{-1}}\gamma_{r}\boldsymbol{D}^{\mathbf{-1}}$$

$$\boldsymbol{=}\gamma_{r}\left( \boldsymbol{D}\mathbf{-}\phi\boldsymbol{D}\tilde{\boldsymbol{K}} \right)^{\mathbf{-1}}$$

$$\boldsymbol{=}\gamma_{r}\left( \boldsymbol{D}\mathbf{-}\phi\boldsymbol{K} \right)^{\mathbf{-1}}$$

We simplify $\boldsymbol{H=D-}\phi\mathbf{K}$ and set the hyperparameter $\phi$ at 0.01. For the cell type-specific scaling factor from the $r$-th reference, $\lambda_{r_{k}}$, we introduce an inverse-gamma–based regularization assumption, $\lambda_{r_{k}}\sim InvG(\alpha_{1},\beta_{1})$. We apply the same regularization structure to the reference-specific scaling factor $\gamma_{r}$, defined as $\gamma_{r}\sim InvG(\alpha_{2},\beta_{2})$. Both$\alpha_{1}$ and $\alpha_{2}$ are set to 1.0, and the scale parameters $\beta_{1}$and $\beta_{2}$ are determined by $\#\frac{spots}{2.0}$, enhancing adaptivity to balance data and prior assumptions effectively^13–15^. For the hyperparameters $s_{r_{k}}$, $q_{r}$ and $\sigma_{e}^{2}$, we employ unconstrained estimation, assuming a constant weight proportional to one across the parameter space.

In this way, we obtain a penalized objective function consisting of the reconstruction loss, CAR-based spatial regularization terms, and additional regularization terms for the scaling parameters.

$$logPr\left( \boldsymbol{P},\lambda_{r_{k}},\sigma_{e}^{2}, \boldsymbol{s}_{r_{k}},\gamma_{r},\boldsymbol{q}_{r}|\boldsymbol{S},\boldsymbol{Y}, \phi, \alpha, \beta\right)$$

$=-\frac{G*n}{2}log\sigma_{e}^{2}-\frac{1}{2\sigma_{e}^{2}}\left| \left| \boldsymbol{Y}-\sum_{r} \boldsymbol{S}_{r}\boldsymbol{P}_{r}^{T}\boldsymbol{Q}_{r} \right| \right|^{2}+\frac{n}{2}\sum_{r} \sum_{k} log\frac{1}{\boldsymbol{\lambda}_{r_{k}}}$ + $\frac{\sum_{r} \boldsymbol{K}_{r}}{2}log\left( det\left( \boldsymbol{H} \right) \right)$

$$-\sum_{r} \sum_{k} \frac{1}{2\lambda_{r_{k}}}\left( \boldsymbol{P}_{r_{k}}-\boldsymbol{s}_{r_{k}}1_{\boldsymbol{n}} \right)^{\boldsymbol{T}}\boldsymbol{H}\left( \boldsymbol{P}_{r_{k}}- \boldsymbol{s}_{r_{k}}1_{\boldsymbol{n}} \right)-\sum_{r} \sum_{k} (\left( \alpha_{1}+1 \right)log\lambda_{r_{k}}+\beta_{1}\lambda_{r_{k}}^{-1})$$

$+\frac{n}{2}\sum_{r} log\frac{1}{\gamma_{r}}$ + $\frac{r}{2}log\left( det\left( \boldsymbol{H} \right) \right)-\sum_{r} \frac{1}{2\gamma_{r}}\left( \boldsymbol{Z}_{r}-\boldsymbol{q}_{r}1_{\boldsymbol{n}} \right)^{\boldsymbol{T}}\boldsymbol{H}\left( \boldsymbol{Z}_{r}-\boldsymbol{q}_{r}1_{\boldsymbol{n}} \right)-\sum_{r} (\left( \alpha_{2}+1 \right)log\gamma_{r}+\beta_{2}\gamma_{r}^{\boldsymbol{-1}})$

We designed an iterative algorithm to carry out constrained penalized optimization, applying nonnegativity constraints to each element of $\boldsymbol{P}_{r}$ and $\boldsymbol{Q}_{r}$, together with a simplex constraint on the rows of $\boldsymbol{P}_{r}$. The optimization objective includes a least-squares reconstruction loss for the normalized ST expression matrix, CAR-based spatial regularization terms for the cell-type proportions and reference weights, and additional regularization terms. In the final implementation, $\boldsymbol{P}_{r}$ is first estimated for each reference and then fixed when estimating $\boldsymbol{Q}_{r}$ and its hyperparameters. Once we obtain the estimated cell-type composition matrix $\boldsymbol{P}_{r}$ and raw reference-weight matrix $\boldsymbol{Q}_{r}$, we normalize the diagonal elements of $\boldsymbol{Q}_{r}$ across references at each spatial location so that the normalized reference weights sum to one. The detailed derivation is as follows.

1. **Update** $\boldsymbol{V}_{\boldsymbol{r}_{\boldsymbol{k}}}$**, the** $\boldsymbol{k}$**-th cell type proportion corresponding to reference** $\boldsymbol{r}$

We extract the negative likelihood related to $\boldsymbol{P}_{r_{k}}$, $\mathbf{L}\left( \boldsymbol{P}_{\boldsymbol{r}_{\boldsymbol{k}}} \right)$, as we want to minimize the negative likelihood

$$\mathbf{L}\left( \boldsymbol{P}_{r_{k}} \right) \propto\frac{1}{2\sigma_{e}^{2}}\left\{ Tr\left( \boldsymbol{Y}-\sum_{r} \sum_{k} \boldsymbol{S}_{r_{k}}\boldsymbol{P}_{r_{k}}^{T}\boldsymbol{Q}_{r} \right)\left( \boldsymbol{Y}-\sum_{r} \sum_{k} \boldsymbol{S}_{r_{k}}\boldsymbol{P}_{r_{k}}^{T}\boldsymbol{Q}_{r} \right)^{T} \right\}+\sum_{r} \sum_{k} \frac{1}{2\lambda_{r_{k}}}\left( \boldsymbol{P}_{r_{k}}- \boldsymbol{s}_{r_{k}}1_{n} \right)^{T}\boldsymbol{H}\left( \boldsymbol{P}_{r_{k}}- \boldsymbol{s}_{r_{k}}1_{n} \right)$$

$$\propto\frac{1}{2\sigma_{e}^{2}}\left\{ Tr(-2\boldsymbol{Q}_{r}\boldsymbol{Y}^{T}\boldsymbol{S}_{r_{k}}\boldsymbol{P}_{r_{k}}^{T}+2\boldsymbol{S}_{r_{k}}\boldsymbol{P}_{r_{k}}^{T}\boldsymbol{Q}_{r}\boldsymbol{Q}_{r}^{T}\boldsymbol{P}_{r_{k}}\boldsymbol{S}_{r_{k}}^{T}+\boldsymbol{S}_{r_{k}}\boldsymbol{P}_{r_{k}}^{T}\boldsymbol{Q}_{r}\left( \sum_{i\neq r} \sum_{h\neq k} \boldsymbol{S}_{i_{h}}\boldsymbol{P}_{i_{h}}^{T}\boldsymbol{Q}_{i} \right)^{T}+ \sum_{i\neq r} \sum_{h\neq k} \boldsymbol{S}_{i_{h}}\boldsymbol{P}_{i_{h}}^{T}\boldsymbol{Q}_{i}\boldsymbol{Q}_{r}^{T}\boldsymbol{P}_{r_{k}}\boldsymbol{S}_{r_{k}}^{T}) \right\}+\sum_{r} \sum_{k} \frac{1}{2\lambda_{r_{k}}}\left( \boldsymbol{P}_{r_{k}}- \boldsymbol{s}_{r_{k}}\boldsymbol{1}_{n} \right)^{T}\boldsymbol{H}\left( \boldsymbol{P}_{r_{k}}- \boldsymbol{s}_{r_{k}}\boldsymbol{1}_{n} \right)$$

We take a derivative of $\mathbf{L}\left( \boldsymbol{P}_{\boldsymbol{r}_{\boldsymbol{k}}} \right)$ respect to $\boldsymbol{P}_{\boldsymbol{r}_{\boldsymbol{k}}}$

$$\frac{\partial\mathbf{L}\left( \boldsymbol{P}_{\boldsymbol{r}_{\boldsymbol{k}}} \right)}{\boldsymbol{P}_{r_{k}}}\propto\frac{1}{2\sigma_{e}^{2}}\left\{ -2\boldsymbol{Q}_{r}\boldsymbol{Y}^{T}\boldsymbol{S}_{r_{k}}+2\boldsymbol{Q}_{r}\boldsymbol{Q}_{r}^{T}\boldsymbol{P}_{r_{k}}\boldsymbol{S}_{r_{k}}^{T}\boldsymbol{S}_{r_{k}}+2\boldsymbol{Q}_{r}\left( \sum_{i\neq r} \sum_{h\neq k} {\boldsymbol{Q}_{i}^{T}\boldsymbol{P}_{i_{h}}\boldsymbol{S}}_{i_{h}}^{T} \right)\boldsymbol{S}_{r_{k}} \right\}+ \frac{1}{2\lambda_{r_{k}}}\left( 2\boldsymbol{H}P_{r_{k}}-2 \boldsymbol{s}_{r_{k}}\boldsymbol{H}\boldsymbol{1}_{n} \right)$$

$$\propto\frac{1}{\sigma_{e}^{2}}\left\{ -\boldsymbol{Q}_{r}\boldsymbol{Y}^{T}\boldsymbol{BS}_{r_{k}}+\boldsymbol{Q}_{r}\boldsymbol{Q}_{r}^{T}\boldsymbol{P}_{r_{k}}\boldsymbol{B}_{r_{k}}^{T}\boldsymbol{S}_{r_{k}}+\boldsymbol{Q}_{r}\left( \sum_{i\neq r} \sum_{h\neq k} {\boldsymbol{Q}_{i}^{T}P_{i_{h}}\boldsymbol{S}}_{i_{h}}^{T} \right)\boldsymbol{S}_{r_{k}} \right\}+ \frac{1}{\lambda_{r_{k}}}\left( \boldsymbol{H}\boldsymbol{P}_{r_{k}}- \boldsymbol{s}_{r_{k}}\boldsymbol{H}\boldsymbol{1}_{n} \right)$$

We plug in $\boldsymbol{H=D-}\phi\boldsymbol{K}$, and based on the multiplicative rule^16–18^, we will update the $k$-th cell type proportion inferered by the $r$-th reference using this formula:

$$\boldsymbol{P}_{r_{k}}\to$$

$$\boldsymbol{P}_{r_{k}} ^{\circ} \frac{\lambda_{r_{k}}\boldsymbol{Q}_{r}\boldsymbol{Y}^{T}\boldsymbol{S}_{r_{k}}+\sigma_{e}^{2}(\phi\boldsymbol{KV}_{r_{k}}+ s_{r_{k}}\boldsymbol{D}\boldsymbol{1}_{n})}{\lambda_{r_{k}}\left( \boldsymbol{Q}_{r}\boldsymbol{Q}_{r}^{T}\boldsymbol{V}_{r_{k}}\boldsymbol{S}_{r_{k}}^{T}\boldsymbol{S}_{r_{k}}+\boldsymbol{Q}_{r}\left( \sum_{i\neq r} \sum_{h\neq k} {\boldsymbol{Q}_{i}^{T}\boldsymbol{P}_{i_{h}}\boldsymbol{B}}_{i_{h}}^{T} \right)\boldsymbol{S}_{r_{k}} \right)+\sigma_{e}^{2}(\boldsymbol{D}\boldsymbol{P}_{r_{k}}+\phi\boldsymbol{s}_{r_{k}}\boldsymbol{K}\boldsymbol{1}_{n})}$$

2. **Update** $\mathbf{Z}_{\mathbf{r}}$**, the location weights for the** $\boldsymbol{r}$**-th reference**

We extract the negative likelihood related to $\boldsymbol{Z}_{r}$, $\mathbf{L}\left( \boldsymbol{Z}_{r} \right)$. Note that $\boldsymbol{Q}_{r}=diag\left( \boldsymbol{Z}_{r} \right)$, where $\boldsymbol{Z}_{r}$ is a $N$-by-1 vector

$$\mathbf{L}\left( \boldsymbol{Z}_{r} \right) \propto\frac{1}{2\sigma_{e}^{2}}\left\{ Tr\left( \boldsymbol{Y}-\sum_{r} \boldsymbol{S}_{r}\boldsymbol{P}_{r}^{T}\boldsymbol{Q}_{r} \right)\left( \boldsymbol{Y}-\sum_{r} \boldsymbol{S}_{r}\boldsymbol{P}_{r}^{T}\boldsymbol{Q}_{r} \right)^{T} \right\}+\sum_{r} \frac{1}{2\gamma_{r}}\left( \boldsymbol{Z}_{r}-\boldsymbol{q}_{r}\boldsymbol{1}_{n} \right)^{T}\boldsymbol{H}\left( \boldsymbol{Z}_{r}-\boldsymbol{q}_{r}\boldsymbol{1}_{n} \right)$$

$$\propto\frac{1}{2\sigma_{e}^{2}}\{Tr(-2\boldsymbol{S}_{r}\boldsymbol{P}_{r}^{T}\boldsymbol{Q}_{r}\boldsymbol{Y}^{T}+\boldsymbol{S}_{r}\boldsymbol{P}_{r}^{T}\boldsymbol{Q}_{r}\boldsymbol{Q}_{r}^{T}\boldsymbol{P}_{r}\boldsymbol{S}_{r}^{T}+2(\sum_{i\neq r} \boldsymbol{S}_{i}\boldsymbol{P}_{i}^{T}\boldsymbol{Q}_{i})\boldsymbol{Q}_{r}^{T}\boldsymbol{P}_{r}\boldsymbol{S}_{r}^{T})\}+\frac{1}{2\gamma_{r}}\left( \boldsymbol{Z}_{r}^{T}\boldsymbol{H}\boldsymbol{Z}_{r}-2\boldsymbol{q}_{r}\boldsymbol{Z}_{r}^{T}\boldsymbol{H}\boldsymbol{1}_{n} \right)$$

$$\propto\frac{1}{2\sigma_{e}^{2}}\{Tr((-2\boldsymbol{S}_{r}\boldsymbol{P}_{r}^{T}\boldsymbol{Q}_{r}\boldsymbol{Y}^{T}{+ 2\boldsymbol{P}_{r}\boldsymbol{S}_{r}^{T}(\sum_{i\neq r} \boldsymbol{S}_{i}\boldsymbol{P}_{i}^{T}\boldsymbol{Q}_{i}))\boldsymbol{Q}}_{r})+{Tr(\boldsymbol{P}_{r}\boldsymbol{S}_{r}^{T}\boldsymbol{S}}_{r}\boldsymbol{P}_{r}^{T}\boldsymbol{Q}_{r}\boldsymbol{Q}_{r}^{T}\}+\frac{1}{2\gamma_{r}}\left( \boldsymbol{Z}_{r}^{T}\boldsymbol{H}\boldsymbol{Z}_{r}-2\boldsymbol{q}_{r}\boldsymbol{Z}_{r}^{T}\boldsymbol{H}\boldsymbol{1}_{n} \right)$$

Let $\boldsymbol{U}_{r}=\boldsymbol{Y}^{T}\boldsymbol{S}_{r}\boldsymbol{P}_{r}^{T}$ and $\boldsymbol{W}_{r}= \boldsymbol{P}_{r}\boldsymbol{S}_{r}^{T}(\sum_{i\neq r} \boldsymbol{S}_{i}\boldsymbol{P}_{i}^{T}\boldsymbol{Q}_{i})$, so both $\boldsymbol{U}_{r}$ and $\boldsymbol{W}_{r}$ are $N$-by-$N$ matrix, and let $\boldsymbol{M}_{r}= {\boldsymbol{P}_{r}\boldsymbol{S}_{r}^{T}\boldsymbol{S}}_{r}\boldsymbol{P}_{r}^{T}$, is also a $N$-by-$N$ matrix, since Trace is the summation of the diagonal matrix, we can get

$$\mathbf{L}\left( \boldsymbol{Z}_{r} \right)\propto\frac{1}{2\sigma_{e}^{2}}\{Tr(-2\boldsymbol{U}_{r}\boldsymbol{Q}_{r}+2\boldsymbol{W}_{r}\boldsymbol{Q}_{r})+Tr(\boldsymbol{M}_{r}\boldsymbol{Q}_{r}\boldsymbol{Q}_{r}^{T})\}+\frac{1}{2\gamma_{r}}\left( \boldsymbol{Z}_{r}^{T}\boldsymbol{H}\boldsymbol{Z}_{r}-2\boldsymbol{q}_{r}\boldsymbol{Z}_{r}^{T}\boldsymbol{H}\boldsymbol{1}_{n} \right)$$

Let $\boldsymbol{w}_{r}$ be the $N$-by-1 vector of all diagonal elements of $\boldsymbol{W}_{r}$, so $Tr\left( \boldsymbol{W}_{r}\boldsymbol{Q}_{r} \right)=$ $\boldsymbol{w}_{r}^{T}\boldsymbol{Z}_{r}$

Similarly, let $\boldsymbol{u}_{r}$ be the $N$-by-1 vector of all diagonal elements of $\boldsymbol{U}_{r}$, so $Tr\left( \boldsymbol{U}_{r}\boldsymbol{Q}_{r} \right)=$ $\boldsymbol{u}_{r}^{T}\boldsymbol{Z}_{r}$

Similarly, let $\boldsymbol{m}_{r}$ be the $N$-by-1 vector of all diagonal elements of $\boldsymbol{M}_{r}$, so

$$Tr\left( \boldsymbol{M}_{r}\boldsymbol{Q}_{r}\boldsymbol{Q}_{r}^{T} \right)= \sum_{i=1}^{N} \boldsymbol{m}_{i}\boldsymbol{Z}_{r_{i}}^{2}$$

Take the derivative of $\mathbf{L}\left( \mathbf{Z}_{r} \right)$ with respect to $\mathbf{Z}_{r}$

$\frac{\partial\mathbf{L}\left( \mathbf{Z}_{r} \right)}{\mathbf{Z}_{r}}\propto\frac{1}{2\sigma_{e}^{2}}${$-2\boldsymbol{u}_{r}+2\boldsymbol{w}_{r}+2\boldsymbol{B}_{r}\boldsymbol{Z}_{r}$} + $\frac{1}{2\boldsymbol{\gamma}_{r}}\left( \boldsymbol{H}\boldsymbol{Z}_{r}-2\boldsymbol{q}_{r}\boldsymbol{H}\boldsymbol{1}_{n} \right)$

$\propto\frac{1}{\sigma_{e}^{2}}${$-\boldsymbol{u}_{r}+\boldsymbol{w}_{r}+\boldsymbol{B}_{r}\boldsymbol{Z}_{r}$} + $\frac{1}{\boldsymbol{\gamma}_{r}}(\boldsymbol{Z}_{r}-\boldsymbol{q}_{r}\boldsymbol{H}\boldsymbol{1}_{n})$

Note that here we use $\boldsymbol{B}_{r}$ to represent the diagonal matrix that the elements from $\boldsymbol{m}_{r}$ on the diagonal line, and for off-diagonal elements, they are all zeros. This is to use matrix format to represent the first derivate of $\sum_{i=1}^{N} m_{i}\boldsymbol{Z}_{r_{i}}^{2}$ with respect to $\boldsymbol{Z}_{r_{i}}$

We plug in $\boldsymbol{H=D-}\phi\boldsymbol{K}$, and again based on the multiplicative rule^16–18^ we will update the $\mathbf{Z}_{r}$ weight vector using this formula:

$$\mathbf{Z}_{r} ->\mathbf{Z}_{r} ^{\circ} \frac{\gamma_{k}\boldsymbol{u}_{r}+\sigma_{e}^{2}(\phi\boldsymbol{K}\boldsymbol{Z}_{r}+\boldsymbol{q}_{r}\boldsymbol{D}\boldsymbol{1}_{n})}{\gamma_{k}\left( \boldsymbol{w}_{r}+\boldsymbol{B}_{r}\boldsymbol{Z}_{r} \right)+\sigma_{e}^{2}(\boldsymbol{D}\boldsymbol{Z}_{r}+{\phi\boldsymbol{q}}_{r}\boldsymbol{K}\boldsymbol{1}_{n})}$$

3. **Update** $\boldsymbol{s}_{rk}$ **and** $\boldsymbol{q}_{r}$

$$\mathbf{L}\left( \boldsymbol{s}_{r_{k}} \right)\propto\frac{1}{2\lambda_{r_{k}}}\left( \boldsymbol{P}_{r_{k}}-\boldsymbol{s}_{r_{k}}1_{\boldsymbol{n}} \right)^{\boldsymbol{T}}\boldsymbol{H}\left( \boldsymbol{P}_{r_{k}}-\boldsymbol{s}_{r_{k}}\boldsymbol{1}_{\boldsymbol{n}} \right)$$

$\frac{\partial\boldsymbol{L}\left( \boldsymbol{s}_{r_{k}} \right)}{\partial\boldsymbol{s}_{r_{k}}}=$ $\frac{1}{\boldsymbol{\lambda}_{r_{k}}}\boldsymbol{P}_{r_{k}}^{T}\boldsymbol{H}\boldsymbol{1}_{n}-\frac{1}{\boldsymbol{\lambda}_{r_{k}}}{\boldsymbol{s}_{r_{k}}\boldsymbol{1}}_{n}^{\boldsymbol{T}}\boldsymbol{H}\boldsymbol{1}_{n}=0$

$\to\boldsymbol{s}_{r_{k}}=\boldsymbol{P}_{r_{k}}^{T}\boldsymbol{H}\boldsymbol{1}_{n}\left( \boldsymbol{1}_{n}^{T}\boldsymbol{H}\boldsymbol{1}_{n} \right)^{\boldsymbol{-1}}$

$$\mathbf{L}\left( \boldsymbol{q}_{r} \right)\propto\frac{1}{2\gamma_{r}}\left( \boldsymbol{Z}_{\boldsymbol{r}}-\boldsymbol{q}_{r}\boldsymbol{1}_{\boldsymbol{n}} \right)^{\boldsymbol{T}}\boldsymbol{H}\left( \boldsymbol{Z}_{\boldsymbol{r}}-\boldsymbol{q}_{r}\boldsymbol{1}_{\boldsymbol{n}} \right)$$

$${\to\boldsymbol{q}}_{r}=\boldsymbol{Z}_{\boldsymbol{r}}^{\boldsymbol{T}}\boldsymbol{H}\boldsymbol{1}_{n}\left( \boldsymbol{1}_{n}^{T}\boldsymbol{H}\boldsymbol{1}_{n} \right)^{\boldsymbol{-1}}$$

4. **Update** $\lambda_{r_{k}}$ **and** $\gamma_{r}$

$\boldsymbol{L}\left( \lambda_{r_{k}} \right)\boldsymbol{\propto}\frac{n}{2}log\frac{1}{\boldsymbol{\lambda}_{r_{k}}}$ $-\frac{1}{2\boldsymbol{\lambda}_{r_{k}}}\left( \boldsymbol{P}_{r_{k}}-\boldsymbol{s}_{r_{k}}1_{\boldsymbol{n}} \right)^{\boldsymbol{T}}\boldsymbol{H}\left( \boldsymbol{P}_{r_{k}}-\boldsymbol{s}_{r_{k}}\boldsymbol{1}_{\boldsymbol{n}} \right)-\left( \alpha_{1}+1 \right)log\lambda_{r_{k}}-\beta_{1}\lambda_{r_{k}}^{-1}$

$$\boldsymbol{\propto-}\left( \frac{n}{2}+\alpha_{1}+1 \right)log\lambda_{r_{k}}-\left( \frac{\left( \boldsymbol{P}_{r_{k}}-\boldsymbol{s}_{r_{k}}\boldsymbol{1}_{\boldsymbol{n}} \right)^{T}\boldsymbol{H}\left( \boldsymbol{P}_{r_{k}}-\boldsymbol{s}_{r_{k}}\boldsymbol{1}_{\boldsymbol{n}} \right)}{2}+\beta_{1} \right)\lambda_{r_{k}}^{\boldsymbol{-1}}$$

$$\frac{\partial L\left( \lambda_{r_{k}} \right)}{\partial\lambda_{r_{k}}}=-\frac{\left( \frac{n}{2}+\alpha_{1}+1 \right)}{\lambda_{r_{k}}}\boldsymbol{+}\frac{1}{\lambda_{r_{k}}^{2}}\left( \frac{\left( \boldsymbol{P}_{r_{k}}-\boldsymbol{s}_{r_{k}}\boldsymbol{1}_{n} \right)^{T}\boldsymbol{H}\left( \boldsymbol{P}_{r_{k}}-\boldsymbol{s}_{r_{k}}\boldsymbol{1}_{n} \right)}{2}+\beta_{1} \right)$$

$$\boldsymbol{\to}\hat{\lambda_{r_{k}}}\boldsymbol{=}\frac{\frac{\left( \boldsymbol{P}_{r_{k}}-\boldsymbol{s}_{r_{k}}\boldsymbol{1}_{n} \right)^{T}\boldsymbol{H}\left( \boldsymbol{P}_{r_{k}}-\boldsymbol{s}_{r_{k}}\boldsymbol{1}_{n} \right)}{2}\boldsymbol{+}\beta_{1}}{\left( \frac{n}{2}+\alpha_{1}+1 \right)}$$

**Similarly,**

$$\boldsymbol{\to}\hat{\gamma_{r}}\boldsymbol{=}\frac{\frac{\left( \boldsymbol{Z}_{r}-\boldsymbol{q}_{r}\boldsymbol{1}_{n} \right)^{T}\boldsymbol{H}\left( \boldsymbol{Z}_{r}-\boldsymbol{q}_{r}\boldsymbol{1}_{n} \right)}{2}\boldsymbol{+}\beta_{2}}{\left( \frac{n}{2}+\alpha_{2}+1 \right)}$$

5. **Update** $\boldsymbol{\sigma}_{\boldsymbol{e}}^{\boldsymbol{2}}$

$$\frac{\partial\boldsymbol{L}\left( \sigma_{e}^{2} \right)}{\partial\sigma_{e}^{2}}=-\frac{G*n}{2\sigma_{e}^{2}}+\frac{\left| \left| \boldsymbol{Y}-\sum_{r} \boldsymbol{S}_{r}\boldsymbol{P}_{r}^{T}\boldsymbol{Q}_{r} \right| \right|^{2}}{2\sigma_{e}^{4}}=0$$

$$\to\hat{\sigma_{e}^{2}}=\frac{\left| \left| \boldsymbol{Y}-\sum_{r} \boldsymbol{S}_{r}\boldsymbol{P}_{r}^{T}\boldsymbol{Q}_{r} \right| \right|^{2}}{G*n}$$

## **Note B. Simulation setting**

To generate the simulation data, we used two mouse olfactory bulb single-cell RNA sequencing (scRNA-seq) data^2,19^ as the basis for our simulations. The two references shared six broad cell types—neurons, astrocytes, oligodendrocytes, vascular cells, immune cells, and ependymal cells—which were used to generate the spatial count data. Although these references contained comparable cell-type labels, UMAP plot (Supplementary Figure 1) of the combined cells showed clear separation by reference identity, indicating substantial reference-specific expression differences. These differences may reflect technical batch effects, platform differences, biological variation, or differences in subtype and cell-state composition.

This reference-level separation motivated the use of spatially varying reference weights. Specifically, one reference was used to generate gene expression in the GCL and ONL regions, whereas the other reference was used to generate gene expression in the MCL region. Thus, different anatomical regions had different reference relevance. We then evaluated performance under three scenarios with different levels of cell-type overlap between references: complete overlap, partial overlap, and no overlap. The inferred reference weight distribution showed that SWIMR assigned higher weight to the reference used to simulate each corresponding region, indicating that SWIMR can recover spatially varying reference relevance in addition to estimating cell-type proportions.

In the first scenario, both references contained the same six cell types: neurons, astrocytes, vascular cells, ependymal cells, immune cells, and oligodendrocytes. In the second scenario, the two references shared some cell types but were not identical. One reference included neurons, astrocytes, ependymal cells, and vascular cells, while the other contained neurons, astrocytes, oligodendrocytes, and immune cells. In the third scenario, there were no overlapping cell types between the two references. Specifically, one reference contained neurons, ependymal cells, and vascular cells, whereas the other included astrocytes, oligodendrocytes, and immune cells.

## **Note C. Details on preprocessing Spatial Transcriptomics and Single Cell RNA-seq**

All cell type information were summarized in Table A

3.1 Human developing human heart

Both spatial data and reference data were obtained from the benchmarking paper^20^. The spatial data contains 66 genes on 1039 locations, and we did not perform any filtering step on it. The internal scRNA-seq data contain 66 genes and 17454 cells and the external scRCA-seq reference was from 10x Genomics data downloaded from EGAS00001003996 containing 65 genes and 3253 cells.

3.2 Lung cancer

We obtained the 10x Visium data from the 10X genomics public datasets (https://www.10xgenomics.com/resources/datasets/human-lung-cancer-ffpe-2-standard). The raw data contains 18,066 genes on 3,858 spatial locations. We filtered out genes that have zero counts on all spatial locations and filtered out spatial locations that have less than 100 total read counts and had 18,066 genes and 3,849 spatial locations remaining.

For the human lung cancer reference data, we obtained it from Salcher et al.^3^ containing 17,797 genes and 91,275 cells in squamous cell lung carcinoma characterized in the original study from different technologies: 10x, BD-Rhapsody, inDrop, Singleron and Smart-seq2, where different technologies emphasis on different aspects: 10x Genomics offers high-throughput single-cell RNA sequencing (scRNA-seq) that captures thousands of cells in parallel, emphasizing speed and efficiency. BD-Rhapsody uses targeted panels to capture and sequence specific genes from single cells, ideal for focused studies. InDrop facilitates the capture of single cells in droplets for RNA sequencing, allowing the analysis of complex cell populations with a high degree of customization. Singleron focuses on single-cell multi-omics technologies that provide detailed data on both gene expression and genetic sequences from individual cells. Smart-seq2 offers highly sensitive full-length transcript coverage for in-depth analysis of gene expression, making it suitable for low-input samples and detailed studies.

3.3 Developing mouse embryo

We also applied deconvolution on human lung cancer data. We obtained mouse embryo data from the 10x genomics website (<https://www.10xgenomics.com/datasets/visium-cytassist-mouse-embryo-11-mm-capture-area-ffpe-2-standard>). This data consists of gene expression for 19,465 genes and 6,434 spatial locations. We filtered out genes that have zero counts on all spatial locations and filtered out spatial locations that have less than 100 total read counts and had 19,404 genes and 6,434 spatial locations remaining.

We obtained the scRNA-seq reference from Fei et al.^5^ where we utilized the data at stage E14.5 containing separate organs including brain, liver, heart and etc. To better map the scRNA-seq to developing mouse embryo, we only picked data with organ brain, liver and heart. Specifically, the scRNA-seq reference from brain tissue contains 19,536 genes and 4,369 cells, among them it includes. The scRNA-seq reference from liver tissue contains 16,488 genes and 2,699 cells including. The scRNA-seq reference from heart tissue contains 18,598 genes and 10,123 cells included.

3.4 Human breast cancer

We obtained the breast cancer data from 10x Visium (https://cf.10xgenomics.com/samples/spatial-exp/1.3.0/Visium_FFPE_Human_Breast_Cancer/Visium_FFPE_Human_Breast_Cancer_web_summary.html). The raw data contains 17,943 genes on 2,518 spatial locations. We filtered out genes that have zero counts on all spatial locations and filtered out spatial locations that have less than 100 total read counts and had 17,651 genes and 2,518 spatial locations remaining.

We downloaded data from Pang et al.^6^ to serve as the scRNA-seq data reference for deconvolution. This dataset collected data from different papers emphasis different aspects: Liu et al: explores the heterogeneity of HER2-positive breast cancer; Wu et al: focus on the microenvironment of triple-negative breast cancer; Xu et al: uncover cellular mechanisms of resistance in breast cancer treatment; Bhupinder et al: characterizes cellular diversity across different stages of breast cancer.

3.5 Hepatocellular carcinoma

We downloaded the hepatocellular carcinoma data from GSE238264^21^ (HCC-patient1-responder). The raw data contains 36,601 genes on 3,006 spatial locations. After filtering out genes with no expression on all spatial locations and those locations with less than 100 total read counts, there were 20,423 genes and 2,736 spatial locations remaining.

We obtained the scRNA-seq reference from GSE156625^7^, GSE149614^9^ and GSE151530^8^ where these studies focused on different parts of the hepatocellular carcinoma: Sharma et al. provide a detailed mapping of the cellular landscape in primary and metastatic HCC, emphasizing immune cell diversity and the identification of prognostic subtypes. Lu et al. focus on the reactivation of fetal programs within the tumor microenvironment, highlighting developmental parallels in HCC. Ma et al. examine the dynamic changes in tumor cell states in response to immunotherapy, providing insights into treatment-induced cellular evolution.

## **Note D. Comparison with a consensus ensemble approach**

To evaluate whether the benefit of SWIMR arises from jointly modeling multiple references rather than simply aggregating results from independently analyzed references, we implemented a consensus ensemble comparator that combines deconvolution results obtained from individual reference panels. For each reference panel, we first applied the single-reference deconvolution method independently to estimate cell-type proportions. We then reconstructed the estimated spatial gene expression matrix $\hat{\boldsymbol{Y}_{r}}$ for reference $r$ as

$$\hat{\boldsymbol{Y}_{r}}=\hat{\boldsymbol{S}_{r}}\hat{\boldsymbol{P}_{r}^{T}}$$

where $\hat{\boldsymbol{S}_{r}}\in R^{G\times K_{r}}$ is the estimated reference expression profile obtained from each method and $\hat{\boldsymbol{P}_{r}}\in R^{N\times K_{r}}$ is the estimated cell-type proportion matrix from reference $r$. We evaluated the reconstruction error for each reference using the sum of squared errors,

$$SSE_{r}=\left| \boldsymbol{|Y}-{\hat{\boldsymbol{Y}}}_{r}| \right|_{F}^{2}$$

where $\boldsymbol{Y}$ is the observed spatial gene expression matrix. Following the rationale used in SCDC, we treated the observed spatial gene expression as the target and assigned each reference a weight proportional to the inverse reconstruction error:

$$w_{r}=\frac{1/SSE_{r}}{\sum_{r^{'}} 1/SSE_{r^{'}}}$$

The final ensemble estimate was obtained as the weighted average of the cell-type proportion estimates across references. For cell types not shared across all references, missing proportions were set to zero before final normalization. This approach therefore provides a post hoc consensus baseline in which references are analyzed independently and subsequently combined according to their reconstruction performance.

Across the simulation settings, SWIMR generally produced more accurate cell-type proportion estimates than the consensus ensemble approach, indicating an advantage of jointly modeling multiple references over post hoc aggregation of single-reference results (Fig AG in S1 Appendix). Under the complete-overlap scenario, the root mean square error (RMSE) were 0.085 for SWIMR, 0.104 for CARD, 0.190 for RCTD, and 0.079 for cell2location. Under partial overlap, the corresponding RMSEs were 0.072, 0.140, 0.167, and 0.185, respectively, while under the non-overlap scenario they were 0.069, 0.216, 0.240, and 0.239. We further evaluated the consensus strategy in the developing human heart dataset (Fig AH in S1 Appendix). SWIMR and the CARD-based ensemble both achieved an RMSE of 0.071, compared with 0.102 for RCTD and 0.171 for cell2location.

Together, these results suggest that simply averaging independently fitted single-reference deconvolution results does not fully capture the benefit of multi-reference integration. By jointly modeling the spatially varying contributions of multiple references within a unified framework, SWIMR can improve deconvolution accuracy while preserving biologically coherent spatial cell-type distributions.

## **Note E. Subtype-specific breast cancer reference analysis**

Cancer studies represent an important setting where SWIMR may be especially useful because tumor transcriptomes can vary substantially across patients and molecular subtypes. In practice, users may wish to combine references from different patients, disease subtypes, or treatment conditions and allow the model to determine which references are most relevant across spatial regions. Here we performed an additional analysis of the HER2-positive breast cancer dataset to illustrate this potential application. The breast cancer spatial sample was confirmed to be HER2-positive based on *ERBB2* expression. We split one breast cancer scRNA-seq reference from Wu et al^22^ into three subtype-specific sub-references representing HER2-positive, ER-positive, and TNBC tumors and compared SWIMR with CARD_single across major cancer cell states, including Cancer Her2 SC, Cancer Cycling, Cancer LumA SC, Cancer LumB SC, and Cancer Basal SC.

The major cancer cell-state maps were broadly consistent between CARD_single and SWIMR, indicating that SWIMR preserved the dominant HER2-like tumor structure while improving the spatial coherence of weaker secondary tumor programs (Fig AI in S1 Appendix). The largest difference was observed for Cancer LumB SC: SWIMR increased its mean proportion from 0.0068 to 0.0111 and its median from 0.0045 to 0.0089, while Moran’s I increased from 0.24 to 0.50, representing the largest increase among the evaluated cancer cell states. Marker-gene analysis further supported the biological relevance of this signal, as SWIMR-derived Cancer LumB SC proportions showed stronger correlations with luminal epithelial markers *FOXA1*, *GATA3*, *KRT8*, and *KRT18*, as well as the proliferation markers *TOP2A* and *MKI67*, whereas *ESR1* and *PGR* remained weakly correlated under both methods. Together, these results suggest that SWIMR can preserve the dominant tumor subtype signal while improving the spatial and biological coherence of secondary tumor programs.

Overall, this analysis suggests that SWIMR can improve the spatial and biological coherence of secondary tumor programs while preserving the dominant HER2-like tumor structure.

## **Note F. Sensitivity analysis of separate spatial parameters for the cell-type proportion and reference-weight layers**

In our current implementation of SWIMR, the cell-type proportion and reference-weight layers use a shared spatial parameter. To evaluate whether this assumption materially affects deconvolution performance, we performed an additional sensitivity analysis in which the two layers were assigned separate spatial parameters, denoted by $\Phi_{P}$ for the cell-type proportion layer and $\Phi_{Q}$ for the reference-weight layer. For each simulation scenario, we used the original simulation setup with all scenarios (complete-overlap, partial-overlap, and non-overlap) but fixed a single simulation instance per scenario (seed = 1) to ensure comparability across experiments. This design holds the simulated dataset and all other model settings constant while varying the relative spatial regularization strength between the two layers. Specifically, we considered $\Phi_{Q}/\Phi_{P}$ ratios of 0.5, 1, 10, 50, and 100. A fixed simulation seed was used within each scenario to ensure that differences in performance reflected changes in the relative spatial parameters rather than variation arising from independently generated simulated datasets.

Across the simulation scenarios, the estimated cell-type proportions and reference weights remained stable over a broad range of layer-specific spatial parameter values (Fig AJ in S1 Appendix). In the complete-overlap scenario, the root mean square error (RMSE) changed only slightly, from 0.082 to 0.084 as $\Phi_{Q}/\Phi_{P}$ increased. Similarly, the RMSE ranged from 0.079 to 0.087 in the partial-overlap scenario and from 0.068 to 0.082 in the non-overlap scenario across the parameter settings examined. These results indicate that SWIMR is relatively robust to differences in the spatial regularization strength applied to the cell-type proportion and reference-weight layers. Thus, using a shared spatial parameter provides a reasonable default while reducing model complexity and the need for additional parameter tuning. Nevertheless, in datasets where the spatial smoothness of cell-type composition and reference relevance differs substantially, allowing layer-specific spatial parameters or bandwidths may provide additional flexibility.

**References**

1.	Zeisel, A. *et al.* Molecular Architecture of the Mouse Nervous System. *Cell* **174**, 999-1014.e22 (2018).

2.	Mizrak, D. *et al.* Single-Cell Analysis of Regional Differences in Adult V-SVZ Neural Stem Cell Lineages. *Cell Rep.* **26**, 394-406.e5 (2019).

3.	Chen, J. *et al.* A comprehensive comparison on cell-type composition inference for spatial transcriptomics data. *Brief. Bioinform.* **23**, bbac245 (2022).

4.	Salcher, S. *et al.* High-resolution single-cell atlas reveals diversity and plasticity of tissue-resident neutrophils in non-small cell lung cancer. *Cancer Cell* **40**, 1503-1520.e8 (2022).

5.	Fei, L. *et al.* Systematic identification of cell-fate regulatory programs using a single-cell atlas of mouse development. *Nat. Genet.* **54**, 1051–1061 (2022).

6.	Pang, L. *et al.* Single-cell integrative analysis reveals consensus cancer cell states and clinical relevance in breast cancer. *Sci. Data* **11**, 289 (2024).

7.	Sharma, A. *et al.* Onco-fetal Reprogramming of Endothelial Cells Drives Immunosuppressive Macrophages in Hepatocellular Carcinoma. *Cell* **183**, 377-394.e21 (2020).

8.	Ma, L. *et al.* Single-cell atlas of tumor cell evolution in response to therapy in hepatocellular carcinoma and intrahepatic cholangiocarcinoma. *J. Hepatol.* **75**, 1397–1408 (2021).

9.	Lu, Y. *et al.* A single-cell atlas of the multicellular ecosystem of primary and metastatic hepatocellular carcinoma. *Nat. Commun.* **13**, 4594 (2022).

10.	Svensson, V., Teichmann, S. A. & Stegle, O. SpatialDE: identification of spatially variable genes. *Nat. Methods* **15**, 343–346 (2018).

11.	Sun, S., Zhu, J. & Zhou, X. Statistical analysis of spatial expression patterns for spatially resolved transcriptomic studies. *Nat. Methods* **17**, 193–200 (2020).

12.	Ma, Y. & Zhou, X. Spatially informed cell-type deconvolution for spatial transcriptomics. *Nat. Biotechnol.* **40**, 1349–1359 (2022).

13.	Vaart, A. W. van der & Zanten, J. H. van. Adaptive Bayesian estimation using a Gaussian random field with inverse Gamma bandwidth. *Ann. Stat.* **37**, 2655–2675 (2009).

14.	Vaart, A. van der & Zanten, H. van. Bayesian inference with rescaled Gaussian process priors. *Electron. J. Stat.* **1**, 433–448 (2007).

15.	Narisetty, N. N. & He, X. Bayesian variable selection with shrinking and diffusing priors. *Ann. Stat.* **42**, 789–817 (2014).

16.	Lee, D. & Seung, H. S. Algorithms for Non-negative Matrix Factorization. in *Advances in Neural Information Processing Systems* vol. 13 (MIT Press, 2000).

17.	Burred, J. J. Detailed derivation of multiplicative update rules for NMF.

18.	Lee, D. D., Seung, H. S. & Saul, L. K. Multiplicative Updates for Unsupervised and Contrastive Learning in Vision.

19.	Zeisel, A. *et al.* Molecular Architecture of the Mouse Nervous System. *Cell* **174**, 999-1014.e22 (2018).

20.	Wang, G., Liu, T., Zhao, J., Cheng, Y. & Zhao, H. Modeling and predicting single-cell multi-gene perturbation responses with scLAMBDA. 2024.12.04.626878 Preprint at https://doi.org/10.1101/2024.12.04.626878 (2024).

21.	Zhang, S. *et al.* Spatial transcriptomics analysis of neoadjuvant cabozantinib and nivolumab in advanced hepatocellular carcinoma identifies independent mechanisms of resistance and recurrence. *Genome Med.* **15**, 72 (2023).

22.	Wu, S. Z. *et al.* A single-cell and spatially resolved atlas of human breast cancers. *Nat. Genet.* **53**, 1334–1347 (2021).
