# Supplementary material for: Enhancing cell type deconvolution in spatial transcriptomics through incorporating multiple reference panels
Source: PLoS Genet. 2026 Nov 2;22(11):e1012345. doi: 10.1371/journal.pgen.1012345 (PMC13726986; doi:10.1371/journal.pgen.1012345)
Supplement: Supplementary file 4 [file pgen.1012345.s005.docx]

**Reviewer1:**

1. **Concerns on identifiability between reference weights and cell-type proportions**

**The core model is given as:**

$$\boldsymbol{Y=}\sum_{\boldsymbol{r}} \boldsymbol{S}_{\boldsymbol{r}}\boldsymbol{P}_{\boldsymbol{r}}^{\boldsymbol{T}}\boldsymbol{Q}_{\boldsymbol{r}}\boldsymbol{+E}\boldsymbol{,}$$

**where both the reference-specific proportions** $\boldsymbol{P}_{\boldsymbol{r}}$ **and the reference weight matrices** $\boldsymbol{Q}_{\boldsymbol{r}}$ **are estimated. Because the model involves products of** $\boldsymbol{P}_{\boldsymbol{r}}$ **and** $\boldsymbol{Q}_{\boldsymbol{r}}$**, there is a potential scaling non-identifiability issue (e.g., scaling** $\boldsymbol{P}_{\boldsymbol{r}}$ **up while scaling** $\boldsymbol{Q}_{\boldsymbol{r}}$ **down may yield similar fits), unless additional constraints are imposed. With this concern, please clarify the following points:**

- **What constraints are imposed to ensure identifiability between** $\boldsymbol{P}_{\boldsymbol{r}}$**and** $\boldsymbol{Q}_{\boldsymbol{r}}$**?**
- **Are the reference weights normalized across references at each location (e.g.,** $\sum_{\boldsymbol{r}} \boldsymbol{Z}_{\boldsymbol{ir}}\boldsymbol{=1}$ **where** $\boldsymbol{Z}_{\boldsymbol{ir}}$ **is the diagonal element of** $\boldsymbol{Q}_{\boldsymbol{r}}$**)?**
- **If no explicit normalization is enforced, how should** $\boldsymbol{Q}_{\boldsymbol{r}}$ **be interpreted quantitatively and compared across references?**

**Please explicitly state any simplex/normalization constraints (if used), or provide a short discussion of identifiability and how the optimization avoids degenerate solutions.**

**Response:** We thank the reviewer for raising this important point regarding identifiability between the reference-specific cell-type proportion matrix $\boldsymbol{P}_{\boldsymbol{r}}$ and the reference-specific scaling matrix $\boldsymbol{Q}_{\boldsymbol{r}}$.

For each reference $r$, the cell-type composition parameters across cell types at each spatial location are constrained such that $\boldsymbol{P}_{ir,k}\geq0$ and $\sum_{k} \boldsymbol{P}_{ir,k}=1$. Thus, $\boldsymbol{P}_{\boldsymbol{r}}$ represents cell-type composition rather than an unrestricted scaling factor. This constraint fixes the scale of $\boldsymbol{P}_{\boldsymbol{r}}$, so that it cannot be arbitrarily rescaled while preserving its interpretation as a cell-type composition.

The diagonal elements of $\boldsymbol{Q}_{\boldsymbol{r}}$, denoted by $\boldsymbol{Z}_{ir}$, are constrained to be nonnegative, location-specific, reference-specific scaling factors. They are not constrained to sum to one across references during model fitting. Therefore, the raw $\boldsymbol{Z}_{ir}$ values should not be interpreted as absolute reference proportions. For interpretation and comparison across references, we normalize the estimated $\boldsymbol{Z}_{ir}$values at each spatial location after model fitting as

$${\tilde{\boldsymbol{Z}}}_{ir}=\frac{\boldsymbol{Z}_{ir}}{\sum_{r^{'}=1}^{R} \boldsymbol{Z}_{ir^{'}}}.$$

so that the resulting normalized reference weights sum to one across references. These normalized quantities are used to describe the relative contribution of each reference at a given spatial location.

The constraints above ensure the identifiability of $\boldsymbol{P}_{\boldsymbol{r}}$ and $\boldsymbol{Q}_{\boldsymbol{r}}$. SWIMR relies on a two-step optimization algorithm to ensure the constraints and estimate the two sets of parameters in an alternative fashion. Specifically, the reference-specific cell-type compositions are first estimated and normalized to satisfy the simplex constraint. These estimated compositions are then treated as fixed when estimating the reference-specific scaling factors $\boldsymbol{Z}_{ir}$. Consequently, during the estimation of $\boldsymbol{Q}_{\boldsymbol{r}}$, the optimization does not permit a compensatory transformation in which $\boldsymbol{P}_{\boldsymbol{r}}$ is arbitrarily increased while $\boldsymbol{Q}_{\boldsymbol{r}}$ is proportionally decreased, or vice versa. Conditional on the fixed $\boldsymbol{P}_{\boldsymbol{r}}$, the magnitude of $\boldsymbol{Z}_{ir}$ is determined by the reconstruction loss relative to the observed spatial expression, while the spatial regularization provides additional stabilization. Thus, the implemented sequential estimation procedure removes the direct $\boldsymbol{P}_{\boldsymbol{r}}$- $\boldsymbol{Q}_{\boldsymbol{r}}$ scaling degeneracy concern raised by the reviewer: the normalization of $\mathbf{P}_{\mathbf{r}}$ ensures the constraints and fixes its compositional scale, while fixing ${\boldsymbol{P}_{\boldsymbol{r}}}_{\mathbf{r}}$ during the subsequent estimation of $\boldsymbol{Q}_{\boldsymbol{r}}$ prevents the two components from compensating for one another within the same optimization. Additional details regarding the normalization and interpretation of $\boldsymbol{Z}_{ir}$ are provided in our response to Comment #2.

We have revised the manuscript to clarify the constraints and normalization applied to these quantities, the interpretation of the estimated reference weights, and how the estimation procedure avoids the direct scaling degeneracy between $\boldsymbol{P}_{\boldsymbol{r}}$ and $\boldsymbol{Q}_{\boldsymbol{r}}$ (page 23, lines 805–814).

2. **Concerns about the interpretation and normalization of reference weights**

**The manuscript refers to** $\boldsymbol{Q}_{\boldsymbol{r}}$ **(or its diagonal elements) as reference weights and uses them to interpret which reference contributes most in a region. However, the current exposition does not make fully clear whether the weights are intended to represent: 1) proportions that sum to 1 across references at each location, or 2) unconstrained nonnegative scaling factors. This distinction matters for interpretability and for claims such as “the model places nearly all weight on the internal reference”.**

**With these concerns, please clarify the following points:**

- **Are weights comparable across references and across locations?**
- **Do weights sum to 1 per location, or are they rescaled post hoc for reporting?**

**Please consider adding one sentence that precisely defines the weight scale (and any normalization) as well as how weights are summarized in figures.**

**Response:** We thank the reviewer for pointing out the need to clarify the interpretation and normalization of the reference weights. In the current implementation of SWIMR, the diagonal elements of $\boldsymbol{Q}_{r}$, denoted by $\boldsymbol{Z}_{ir}$, are estimated as nonnegative reference-specific scaling factors. They are not constrained to sum to one across references during model fitting. Therefore, the raw $\boldsymbol{Z}_{ir}$ values should not be interpreted as absolute reference proportions.

For interpretation, visualization, and regional summaries, we post hoc normalize the estimated weights across references at each spatial location:

$${\tilde{\boldsymbol{Z}}}_{ir}=\frac{\boldsymbol{Z}_{ir}}{\sum_{r^{'}=1}^{R} \boldsymbol{Z}_{ir^{'}}}.$$

By construction, $\sum_{r} {\tilde{\boldsymbol{Z}}}_{ir}=1$ for each location $i$. Thus, ${\tilde{\boldsymbol{Z}}}_{ir}$ represents the relative contribution of reference $r$ at location $i$. The normalized weights can be compared across references at the same location and across locations to characterize the spatial variation in relative reference relevance. This comparison is biologically informative because different tissue regions may contain distinct cell types, cellular states, or microenvironmental programs, and a reference derived from a particular tissue context, disease subtype, or experimental platform may therefore represent some regions more accurately than others. Mapping $\tilde{Z}_{ir}$across the tissue allows us to identify where each reference is most compatible with the local transcriptional composition and to assess whether these spatial patterns correspond to known anatomical structures, pathological regions, or tissue boundaries.

Claims such as “the model places nearly all weight on the internal reference” refer to these normalized relative weights, not the raw scaling factors. We have revised the manuscript to explicitly define this normalization and to clarify that all reference-weight figures and summaries are based on $\tilde{Z}_{ir}$. (page 21 line 731-735 and page 23 line 811-815)

3. **Concerns on shared spatial autocorrelation parameter for two distinct spatial processes**

**The method applies CAR modeling to both: 1) cell-type proportions** $\boldsymbol{P}_{\boldsymbol{r}}$**, and 2) reference weights (diagonal elements of** $\boldsymbol{Q}_{\boldsymbol{r}}$**), apparently using the same spatial kernel/adjacency matrix K and the same spatial autocorrelation parameter** $\boldsymbol{\Phi}$**. This implies that spatial smoothness of cell composition and spatial smoothness of reference relevance follow the same spatial dependence structure and strength, which may be a strong assumption. Reference relevance could plausibly vary more sharply (or differently) than cell-type proportions.**

**With these concerns, please clarify the following points:**

**Please justify why the same** $\boldsymbol{\Phi}$ **(and/or the same** $\boldsymbol{\kappa}$**) is appropriate for both layers.**

**Consider allowing separate spatial parameters (e.g.,** $\boldsymbol{\Phi}_{\boldsymbol{P}}$ **and** $\boldsymbol{\Phi}_{\boldsymbol{Q}}$**) or discuss this as a limitation/future extension.**

**Response:** We thank the reviewer for this helpful comment. In current SWIMR implementation, we use the same spatial kernel $\Phi$, constructed from the spatial coordinates with bandwidth $\kappa$, for both the cell-type proportion layer and the reference-weight layer. This choice is motivated by the fact that both the cell-type proportions and reference relevance are defined over the same tissue locations and are influenced by the same underlying tissue architecture. Spatially adjacent locations are likely to share similar cell-type compositions, local histological context, transcriptional states, and microenvironmental features. These shared local characteristics may also cause neighboring locations to favor similar scRNA-seq references. Thus, using a common spatial neighborhood structure provides a biologically motivated and parsimonious mechanism for borrowing information across nearby locations while avoiding the introduction of additional weakly identifiable spatial parameters.

We acknowledge, however, that the spatial smoothness of reference relevance may differ between the two layers. To evaluate whether the shared-parameter assumption materially affects the results, we performed an additional sensitivity analysis in which the cell-type proportion and reference-weight layers were assigned separate spatial parameters, denoted by $\Phi_{P}$ and $\Phi_{Q}$, respectively (Supplementary Note 6 and Supplementary Figure 36). For each simulation scenario, we held the simulated dataset and all other model settings fixed and varied $\Phi_{Q}$ relative to $\Phi_{P}$, considering $\Phi_{Q}/\Phi_{P}$ ratios of 0.5, 1, 10, 50, and 100. A fixed simulation seed was used (seed=1) for each scenario so that the analysis isolated the effect of changing the relative spatial regularization strength rather than variation arising from generating different simulated datasets.

Across the simulation settings, the estimated cell-type proportions and reference weights remained stable over a broad range of layer-specific spatial parameter values (Supplementary Figure 36). In the complete-overlap scenario, the mean RMSE increased only slightly, from 0.082 to 0.084, when $\Phi_{P}$ was increased to 50 times $\Phi_{P}$. Similarly, in the partial-overlap and non-overlap scenarios, the RMSE ranged from 0.079 to 0.087 and from 0.068 to 0.082, respectively. These analyses indicate that SWIMR is robust to moderate differences in spatial smoothness between the two layers and that the shared specification provides a reasonable default with lower model complexity. Nevertheless, datasets with abrupt changes in reference relevance may benefit from separately estimated or tuned spatial parameters. We have therefore clarified this assumption in the Methods (page 22 line 774-784) and added layer-specific kernels and bandwidths as a potential extension in the Discussion (page 20 line 687-694).

**Supplementary Figure 36. Sensitivity analysis of cell-type proportion estimation accuracy across all simulation scenarios, measured by root mean squared error (RMSE), with the ratio of the reference-weight spatial parameter to the cell-type proportion spatial parameter** $\boldsymbol{\Phi}_{\boldsymbol{Q}}\boldsymbol{/}\boldsymbol{\Phi}_{\boldsymbol{P}}$ **set to 0.5, 1, 10, 50, and 100.**

4. **Concerns on Gaussian noise assumption for count-based ST data**

**The model assumes a Gaussian error term** $\boldsymbol{E}$ **(e.g.,** $\boldsymbol{E}_{\boldsymbol{gn}} \boldsymbol{\sim}\boldsymbol{N (0,}\boldsymbol{\sigma}^{\boldsymbol{2}}\boldsymbol{)}$**). Since ST platforms often produce count data with overdispersion and sparsity, it would be helpful to justify this assumption.**

**So please clarify the following points:**

**Is** $\boldsymbol{Y}$ **modeled on raw counts, normalized counts, or transformed values (e.g., log-normalized)?**

**If counts are used, why is Gaussian noise appropriate versus a count model (Poisson/Negative Binomial)?**

**A brief discussion acknowledging this modeling choice and its implications would strengthen the method.**

**Response:** We thank the reviewer for raising this important point. We have revised the manuscript to clarify the preprocessing of the spatial transcriptomics expression matrix more clearly. Following CARD, we perform library-size normalization before fitting SWIMR. Specifically, the expression profile at each spatial location is divided by the total counts at that location. Therefore, $Y$ denotes a library-size-normalized, nonnegative expression matrix rather than the raw count matrix.

The Gaussian error term is used as a working likelihood that gives rise to the least-squares reconstruction objective

$$Y\approx\sum_{r} S_{r}P_{r}^{T}Q_{r},$$

rather than as a complete generative model for the sequencing count process. This formulation is consistent with existing NMF-based deconvolution frameworks, including CARD, in which a Frobenius-norm reconstruction loss is used to decompose a normalized nonnegative expression matrix into biologically interpretable components. The primary goal of SWIMR is therefore to accurately reconstruct the normalized spatial expression matrix and estimate cell-type compositions and relative reference contributions, rather than to model the original count-generation process explicitly.

We have revised the Methods (page 21 line 708) to describe the normalization of $Y$ and the reference matrices explicitly and to clarify the role of the Gaussian working model in the penalized NMF objective. We have also noted in the Discussion that future extensions could replace the current least-squares loss with a Poisson or negative binomial objective to model sparsity and overdispersion more directly. (page 20 line 683-687)

5. **Concerns on mechanism of reference “competition” vs. additive contribution**

**The formulation is additive across references, but it is not fully clear whether and how references compete such that a better-matched reference is emphasized while others are down-weighted. Empirically, SWIMR appears to prioritize contextually relevant references in simulations and real data, but the mechanism could be described more explicitly. Please clarify the following points:**

- **Does the optimization induce competition through normalization, constraints, or regularization?**
- **How does the model avoid uniform weighting in mismatched scenarios?**

**Response:** We thank the reviewer for raising this important point. Although the SWIMR reconstruction is additive across references, the reference weights are estimated jointly within a shared reconstruction objective and therefore are not assigned independently. A reference receives a larger weight when its reference-based reconstruction better explains the observed spatial expression profile after accounting for the other references. Conversely, if a reference is poorly matched to a spatial region, increasing its weight does not substantially reduce the reconstruction error, and the estimated weight tends to be small. Therefore, reference “competition” in SWIMR arises primarily from the shared reconstruction loss together with the nonnegativity constraints on the reference weights. Because all references contribute to the same reconstruction, assigning more weight to a well-matched reference reduces the remaining expression signal that can be explained by less compatible references. The nonnegativity constraints also prevent references from offsetting one another through positive and negative contributions. This mechanism is data adaptive rather than a hard winner-take-all rule. The spatial regularization further encourages neighboring spots with similar tissue context to have similar reference-weight patterns, but it does not itself force the references to contribute equally. After optimization, the raw weights are normalized across references at each location so that reference contributions can be interpreted on a relative scale. The post hoc normalization of the estimated weights is used only for interpretation and visualization; it does not induce the competition during model fitting. Under this normalization, if one reference better matches the local expression structure, its normalized weight increases while the relative weights of less-matched references decrease.

We agree that SWIMR does not explicitly impose a hard exclusion rule for mismatched references. Uniform weighting is not ruled out by the constraints alone; instead, the reference weights are learned from the data. Poorly matched references are expected to receive smaller weights because they contribute less to reducing the reconstruction loss and explaining the observed spatial expression patterns. For example, in our developing human heart analysis, the internal reference alone was sufficient for deconvolution, whereas incorporating the external reference introduced additional noise. Accordingly, SWIMR assigned a relatively small weight to the external reference. Therefore, SWIMR can down-weight less compatible references rather than assuming equal contributions from all references.

As with other reference-based deconvolution approaches, the candidate references should collectively contain biologically relevant cell types and transcriptional states. We have added this point to the discussion section. (page 19 line 671-674)

**Minor Comments**

1. **The Methods mention maximum likelihood optimization, but the likelihood/objective function is not written explicitly. Including it (even briefly) would improve reproducibility and clarity.**

**Response:** We thank the reviewer for this helpful suggestion. In the original manuscript, the full objective was in the supplementary text (Supplementary Note 1): “In this way, we obtain a penalized objective function consisting of the reconstruction loss, CAR-based spatial regularization terms, and additional regularization terms for the scaling parameters.

$$logPr\left( \boldsymbol{P},\lambda_{r_{k}},\sigma_{e}^{2}, \boldsymbol{s}_{r_{k}},\gamma_{r},\boldsymbol{q}_{r}|\boldsymbol{S},\boldsymbol{Y}, \phi, \alpha, \beta\right)$$

$=-\frac{G*n}{2}log\sigma_{e}^{2}-\frac{1}{2\sigma_{e}^{2}}\left| \left| \boldsymbol{Y}-\sum_{r} \boldsymbol{S}_{r}\boldsymbol{P}_{r}^{T}\boldsymbol{Q}_{r} \right| \right|^{2}+\frac{n}{2}\sum_{r} \sum_{k} log\frac{1}{\boldsymbol{\lambda}_{r_{k}}}$ + $\frac{\sum_{r} \boldsymbol{K}_{r}}{2}log\left( det\left( \boldsymbol{H} \right) \right)$

$$-\sum_{r} \sum_{k} \frac{1}{2\lambda_{r_{k}}}\left( \boldsymbol{P}_{r_{k}}-\boldsymbol{s}_{r_{k}}1_{\boldsymbol{n}} \right)^{\boldsymbol{T}}\boldsymbol{H}\left( \boldsymbol{P}_{r_{k}}- \boldsymbol{s}_{r_{k}}1_{\boldsymbol{n}} \right)-\sum_{r} \sum_{k} (\left( \alpha_{1}+1 \right)log\lambda_{r_{k}}+\beta_{1}\lambda_{r_{k}}^{-1})$$

$+\frac{n}{2}\sum_{r} log\frac{1}{\gamma_{r}}$+$\frac{r}{2}log\left( det\left( \boldsymbol{H} \right) \right)-\sum_{r} \frac{1}{2\gamma_{r}}\left( \boldsymbol{Z}_{r}-\boldsymbol{q}_{r}1_{\boldsymbol{n}} \right)^{\boldsymbol{T}}\boldsymbol{H}\left( \boldsymbol{Z}_{r}-\boldsymbol{q}_{r}1_{\boldsymbol{n}} \right)-\sum_{r} (\left( \alpha_{2}+1 \right)log\gamma_{r}+\beta_{2}\gamma_{r}^{\boldsymbol{-1}})$

We designed an iterative algorithm to carry out constrained penalized optimization, applying nonnegativity constraints to each element of $\boldsymbol{P}_{r}$ and $\boldsymbol{Q}_{r}$, together with a simplex constraint on the rows of $\boldsymbol{P}_{r}$. The optimization objective includes a least-squares reconstruction loss for the observed ST expression matrix, CAR-based spatial regularization terms for the cell-type proportions and reference weights, and additional regularization terms. In the final implementation, $\boldsymbol{P}_{r}$ is first estimated for each reference and then fixed when estimating $\boldsymbol{Q}_{r}$ and its hyperparameters. Once we obtain the estimated cell-type composition matrix $\boldsymbol{P}_{r}$ and raw reference-weight matrix $\boldsymbol{Q}_{r}$, we normalize the diagonal elements of $\boldsymbol{Q}_{r}$across references at each spatial location so that the normalized reference weights sum to one. The detailed derivation is as follows.”

In the revised manuscript, we now make it clear that detailed objective function is provided in Supplementary Note 1. (page 23 line 798-799)

2. **Consider adding runtime and memory benchmarks comparing SWIMR to baseline methods, especially since scalability is a practical concern and SCDC is excluded in real data due to computational burden.**

**Response:** We thank the reviewer for this helpful suggestion. We agree that runtime and memory usage are important practical considerations, especially for multi-reference spatial deconvolution methods and large spatial transcriptomics datasets. In the revised manuscript, we added runtime and peak memory benchmarks comparing SWIMR with CARD, RCTD, SCDC, and cell2location across three spatial transcriptomics datasets: human heart ISS, mouse olfactory bulb ST, and human breast cancer Visium data.

The new results are included in Supplementary Figure 34 (and below) and Supplementary Table 5. SWIMR remained computationally efficient, achieving runtime comparable to CARD while being substantially faster than RCTD, SCDC (where completed), and cell2location across datasets. We also added to our discussion (page 19 line 646-651).

**Supplementary Figure 34. Computation time and peak memory usage for each deconvolution method across three spatial transcriptomics datasets.** Computation time is shown in minutes in the top panel, and peak memory usage is shown in MB in the bottom panel. SWIMR, CARD, RCTD, and SCDC were run in R using a single CPU core on an Intel(R) Xeon(R) Platinum 8268 CPU @ 2.90GHz processor under Ubuntu 22.04.5 LTS. cell2location was run in Python using an NVIDIA H100 NVL GPU. For methods run separately across multiple references, runtimes were summed across reference-specific runs, while peak memory usage was reported as the maximum peak memory observed across runs because the runs were performed sequentially. For cell2location, the memory panel reports CPU peak memory increase to make it comparable with the R-based methods; GPU memory allocation was recorded separately and was not included in the main memory comparison because CPU RAM and GPU memory are distinct hardware resources. SCDC was not completed for the human breast cancer Visium dataset due to its long runtime, exceeding three days.

**Reviewer #2: The choice of reference in spatial transcriptomic deconvolution is often a headache in practice. The proposed SWIMR method is timely, and it has been evaluated by comprehensive studies. I expect it will be widely used. My comments are mostly for clarifications.**

**1. Clarification of simulation setup.**

**The authors "assigned one scRNA-seq reference to simulate gene expression in the GCL and ONL regions, and a second reference for the MCL region". It is not clear how similar are the two references? They both cover the same 6 cell types? In real data, the cells classified as the same cell types from two studies could have different expression, though there can be many reasons. For example, it could be batch effect, or it could be biology, e.g., the difference in the proportion of cell subtypes or cell states.**

**Response:** We thank the reviewer for this helpful comment. The two scRNA-seq references used in the simulation contain comparable broad cell-type annotations, including the six cell types used in the complete-overlap setting. However, they are not identical at the expression level. As the reviewer pointed out, cells assigned to the same broad cell-type label across different studies may still differ due to technical batch effects, platform differences, biological variation, or differences in subtype and cell-state composition. To characterize these differences more clearly, we have modified the manuscript to include a UMAP visualization (main text page 6 line 163, Supplementary Note 2 line 443-460, Supplementary Figure 1) of the combined cells from the two references. The UMAP shows that cells from the two references are largely separated by reference identity, indicating clear reference-specific expression differences. This separation suggests that even when the two references contain comparable broad cell-type labels, they may represent different transcriptional profiles.

This difference is an important part of our simulation design. We used one reference to simulate gene expression in the GCL and ONL regions and the other reference to simulate gene expression in the MCL region, creating a setting in which different spatial regions are better represented by different scRNA-seq references. Therefore, the simulation evaluates not only cell-type proportion accuracy, but also whether SWIMR can recover spatially varying reference relevance. The inferred reference weight distribution shows that SWIMR assigns higher weight to the reference used to generate each corresponding spatial region, indicating that SWIMR can capture reference-specific expression differences across tissue regions. We have modified the supplementary text accordingly to clarify the differences between the two references and to better explain the rationale and significance of this simulation design.

**Supplementary Figure 1. UMAP plot of the two distinct scRNA-seq references from Zeisel et al.^1^ (SRP135960) and Mizrak et al.^2^ (GSE109447)**

**2. the author claim that the most challenging case is "the no-overlap scenario, where each reference contains a completely distinct set of cell types."
It might be helpful to elaborate on this. Why is this the most challenging cases? I am thinking the criteria to decide which reference is used. If two references both have one cell types, it might be harder to decide which reference to use? or the criteria is the deconvolution accuracy.**

**Response:** We thank the reviewer for this helpful comment. In response to your comment, we have removed the phrase “most challenging” from the revised manuscript and now refer to it directly as a no-overlap scenario to avoid potential confusion.

Additionally, we have revised the text to clarify the purpose of the no-overlap scenario (page 7, line 197). In this scenario, each reference contains a distinct set of cell types, so no single reference provides complete cell-type coverage for the spatial transcriptomics data. For example, one reference may contain only immune cell types such as T cells, whereas another reference may contain epithelial, stromal, or myeloid populations. In such settings, the model must borrow complementary information across references to reconstruct the full spatial expression profile while avoiding incorrect assignment of expression to cell types absent from a given reference. From this aspect, this may represent a more difficult setting than scenarios where multiple references contain overlapping cell types, because shared cell types provide anchors for comparing and aligning references.

We also agree that, if the objective were solely to determine which of two highly similar references is more relevant, a setting in which both references contain the same cell type could also be challenging. On the other hand, the non-overlap simulation does address a different practical question: whether accurate deconvolution can be achieved when no single reference adequately represents the full cellular composition. SWIMR avoids the need to select one reference a priori by incorporating all candidate references and learning their spatially varying contributions from the data. It can therefore combine complementary information across incomplete references and assign greater weight to the references that better explain each spatial region. To balance the view, we have removed the phrase “most challenging” and refer to it directly as a no-overlap scenario.

**3. For the 1st real data analysis. Two references are used. "The internal reference, directly obtained from the original study, shares the same set of cell types as the ST data. The external reference, generated using 10x Genomics technology, was manually annotated to assign cell types".**

**It would be helpful to clarify in the main text here, rather than in methods, what platform or technology of the internal reference. It would be also helpful to evaluate the consistency of cell type-specific gene expression between the references.**

**Response:** We thank the reviewer for this helpful suggestion. We have revised the main text to clarify the platform of the internal reference in the developing human heart analysis. Specifically, we now state that the internal reference was obtained from the original study and generated from the matched *in situ* sequencing-based dataset, whereas the external reference was generated using 10x Genomics technology and manually annotated to assign cell types.

In addition, we added a new analysis to evaluate the consistency of cell type-specific gene expression between the two references (Supplementary Figure 5 and below). We compared cell type-specific average expression profiles for matched cell types across the internal and external references. Most matched cell types showed strong agreement (correlations 0.72–0.89 for 9 of 11 shared types), with highest values for fibroblast-like, smooth muscle/fibroblast-like, subepicardial, and ventricular cardiomyocytes; endothelium/pericytes/adventitia showed moderate agreement, and cardiac neural crest-related cells showed low consistency.

These results indicate that the two references are broadly concordant for most major cell types, while still retaining reference-specific differences for several populations. This comparison provides additional context for the inferred reference weights and helps explain why SWIMR assigns nearly all normalized weight to the internal reference, which is more closely matched to the spatial dataset in both platform and biological context. We have added these clarifications and results to Result section in the main text (page 8 line 223-235).

**Supplementary Figure 5. Cell-type-specific expression correlation between references for human heart data.**

**4. for the Human Lung Cancer 10x Visium data. It is interesting that "Smart-seq2 and inDrop contributed most prominently to the deconvolution" To further validate these findings, it would helpful if you chose some spots that are dominated by one cell type, and assume they represent the cell type-specific gene expression from Visium. Then compare them with reference from 10x platform, Smart-seq2 or inDrop?**

**Response:** We thank the reviewer for this helpful suggestion. To further validate the inferred reference-weight pattern in the human lung cancer analysis, we added an analysis comparing cell type-specific expression profiles with the corresponding profiles from each scRNA-seq reference. Specifically, for each cell type, we selected spatial spots dominated by that cell type based on the SWIMR-estimated cell-type proportions and treated the average expression profile of these spots as an approximate cell type-specific expression profile. We then computed correlations between gene expression from spatial data and the corresponding cell type-specific average expression from each scRNA-seq reference, including 10x Genomics, BD-Rhapsody, inDrop, Singleron, and Smart-seq2, when the cell type was available in the reference. This analysis showed that reference similarity was cell-type dependent. including tumor cells (0.781), macrophages (0.655), and monocytes (0.572), whereas Smart-seq2 showed the strongest representation for T cell populations, including CD4 T cells (0.575), CD8 T cells (0.582), and regulatory T cells (0.511). Other references also showed strong agreement for specific cell types, such as BD-Rhapsody for alveolar macrophages, NK cells, and neutrophils, and Singleron for stromal, mast, plasma, and club cell populations. These results indicate that no single reference is uniformly optimal across all cell types. Instead, different references better represent different cellular compartments (Supplementary Figure 7 and below)

This additional analysis supports the inferred SWIMR reference-weight distribution, in which inDrop and Smart-seq2 contributed prominently to the deconvolution. In particular, these two references matched major tumor and immune compartments in the lung cancer tissue, providing additional evidence that SWIMR assigns reference weights in a biologically meaningful and data-adaptive manner. We have added this analysis and the corresponding result to the manuscript (page 9 line 272-289).

**Supplementary Figure 7. Similarity (pearson correlation) between ST cell type proportion and scRNA-seq references in human lung cancer data**.

**5. For cancer studies, a challenge is that the cancer transcriptome from different patients is often very different. This may be a situation, for example, beneficial to combine different references that represent different tumor subtypes, and thus a situation that SWIMR could be very helpful. For the lung cancer and breast cancer data, how many patients are there and are the results (in terms of utilization of references) differ across patients?**

**Response:** We thank the reviewer for raising this important point. We agree that cancer applications represent a setting where multi-reference integration can be especially useful, because tumor transcriptomic profiles often vary substantially across patients and molecular subtypes.

In our current lung and breast cancer analyses, each spatial transcriptomics dataset was obtained from only one patient. Because there are no additional patients within the same cancer type for comparison, we cannot distinguish patient-specific variation in reference utilization from variation across spatial locations within a single tumor. We have revised the manuscript to state the number of patients explicitly and to acknowledge that evaluating patient-to-patient variation in reference weights will require spatial transcriptomics datasets from multiple patients analyzed within the same cancer type.

Although the current datasets do not permit a direct patient-level comparison, we added analysis focusing on evaluating whether SWIMR can distinguish references representing different tumor subtypes within a single spatial sample. Specifically, for the breast cancer analysis, the spatial dataset was identified as HER2-positive based on *ERBB2* expression. To further evaluate the utility of subtype-specific references, we performed an additional analysis in which a single breast cancer scRNA-seq reference was split into three subtype-specific sub-references: HER2-positive, ER-positive, and TNBC. This setup mimics a practical scenario where users combine references representing different breast cancer subtypes. SWIMR assigned strong contribution to the HER2-related reference in tumor regions, consistent with the ERBB2-positive status of the spatial sample. At the same time, SWIMR also captured secondary luminal-like tumor structure. Specifically, compared with CARD_single, SWIMR preserved the dominant HER2-like tumor structure but produced a more spatially coherent Cancer LumB SC signal. The Moran’s I for Cancer LumB SC increased from 0.24 under CARD_single to 0.50 under SWIMR, while the dominant HER2-like and cycling cancer states showed smaller changes. This LumB-like signal was also better supported by marker-gene correlations: SWIMR Cancer LumB SC showed stronger correlations with luminal epithelial markers including *FOXA1*, *GATA3*, *KRT8*, and *KRT18*, as well as proliferation markers *TOP2A* and *MKI67*. These results suggest that SWIMR can preserve the dominant subtype signal while improving the spatial and biological coherence of weaker secondary tumor programs.

We have added this analysis to the Supplementary Note 5 (line 595-622) and included a discussion (page 20 line 675-682) noting that, while the current spatial datasets do not allow evaluation across multiple spatial patients, these results support the potential utility of SWIMR for cancer studies where multiple references represent distinct patient- or subtype-specific tumor states.

**Supplementary Figure 35. Subtype-specific reference analysis in breast cancer.** **A**. spatial distribution of subtype-specific reference weights. **B**. gene expression pattern of marker gene ERBB2. **C**. marker-gene correlations showing biological support for the Cancer LumB single-cell signal identified by SWIMR.

**6. In general, using data from different references means the batch effect may play a role. Any comments or suggestions on how to handle such batch effect? Should users try to evaluate batch effect, such as align the reference with each other before running SWIMR?**
**Response:** Thank you for the insightful comment. We agree with the reviewer that batch effects may arise when integrating references generated from different studies, platforms, or sample-processing protocols. In cancer applications, this issue is further complicated by the fact that technical variation can be difficult to distinguish from true biological variation, such as differences across patients, tumor subtypes, or disease states.

In our real-data analyses, we did not apply an additional batch-correction procedure to align all references before running SWIMR. Some of the scRNA-seq datasets had already been batch corrected or integrated in their original studies (e.g., lung cancer data and breast cancer data), whereas others were used after standard quality control and normalization without further correction (e.g., mouse embryo data and hepatocellular carcinoma data). We chose not to impose a uniform cross-reference alignment because batch correction itself can be difficult to calibrate. In particular, aggressive alignment may remove biologically meaningful reference-specific signals or force genuinely distinct cell states into an artificially shared expression space. This concern has also motivated recent methods, such as CellANOVA^3^, that explicitly aim to distinguish unwanted batch variation from biological heterogeneity and reduce overcorrection.

SWIMR is not intended to eliminate batch effects directly. Rather, it reduces the need to force all references into a single corrected expression space by modeling each reference separately and estimating its spatially varying contribution to the observed ST data. A reference whose expression profile is less compatible with a particular tissue region—whether because of technical or biological differences—will generally contribute less to reducing the reconstruction error and may therefore receive a smaller weight. This adaptive weighting can provide some robustness to reference heterogeneity, but it should not be interpreted as a substitute for appropriate quality control or batch-effect assessment.

In practice, we recommend that users perform standard quality control and normalization for each reference and carefully examine whether the major differences across references are likely to be technical or biological. If strong technical batch effects are suspected within a reference dataset, users may apply commonly used batch-correction or integration procedures^4–6^ before running SWIMR. However, we do not recommend overly aggressive alignment across biologically distinct references, as this may remove meaningful patient- or subtype-specific signals. When feasible, users may also compare results obtained with uncorrected and batch-corrected references as a sensitivity analysis.

Overall, as long as the major cell types are accurately annotated and relevant biological heterogeneity is preserved, SWIMR can accommodate reference differences through its adaptive reference-weighting framework. We included this in our discussion. (page 19 line 664-671)

**A minor concern.**

**1. the authors claim that EPCAM is an onco-gene. The relatively higher expression of EPCAM may indicate more aggressive breast cancer metastasis (based on the reference cited in this paper), but in general, it is highly expressed in both tumor and normal epithelial. It might be better to rephrase it to make it more specific based on the evidence from the reference, such as "EPCAM, which is known to be over-expressed in metastatic breast cancer"**

**Response:** We thank the reviewer for pointing out this concern. We have revised the wording in the Results section (page 14 line 492-494) to more precisely describe *EPCAM* as an epithelial marker reported to be overexpressed in metastatic breast cancer, rather than referring to it broadly as an oncogene.

**Reviewer3**

1. **Several aspects of the model in (1) and (2) do not make sense.
   - The authors constrain P and Q to be nonnegative, but then use a Gaussian model for P. This does not make sense conceptually, and I was wondering why the initial nonnegativity constraints were need.**

**- The authors specify a conditional model (2) for the P’s but then use it to imply a joint distribution. How that works is unclear to me.**

**Response:** We thank the reviewer for raising this important point. We agree that the original presentation did not clearly explain how the nonnegativity constraints and Gaussian spatial assumption are imposed simultaneously. SWIMR is formulated as a constrained nonnegative matrix factorization model with an additional conditional autoregressive (CAR) prior on the cell type composition matrix $\boldsymbol{P}_{r}$. The nonnegativity and normalization constraints define the biologically meaningful parameter space: $\boldsymbol{P}_{r}$ represents reference-specific cell-type proportions and is constrained to be nonnegative and to sum to one at each spatial location, whereas $\boldsymbol{Q}_{r}$, or equivalently its diagonal elements $Z_{ir}$, is constrained to be nonnegative. These constraints are necessary because negative cell-type proportions or negative reference contributions are not biologically interpretable, and the simplex constraint ensures that $\boldsymbol{P}_{r}$ has a fixed and interpretable scale.

The Gaussian CAR specification serves a different purpose. It is used to characterize spatial dependence among neighboring locations and induces a quadratic regularization term that favors similar values of $\boldsymbol{P}_{r}$ at neighboring spatial locations. Importantly, $\boldsymbol{P}_{r}$ is not estimated as an unrestricted Gaussian variable. Rather, SWIMR minimizes the resulting penalized reconstruction objective subject to the nonnegativity and simplex constraints on $\boldsymbol{P}_{r}$. Thus, there is no conceptual conflict: the Gaussian CAR assumption determines the spatial regularization structure, while the additional nonnegativity and simplex constraints define the feasible parameter space during optimization. This is equivalent to an MAP-type estimator with a CAR smoothness penalty under constrained optimization. The same principle is applied to the spatially varying reference weights. (page 23, lines 790–799).

We have also revised the description of model (2) to avoid implying that the conditional specification alone automatically defines a joint distribution. In the revised manuscript, the CAR component is presented through its corresponding Gaussian spatial prior and penalization term in the objective function. This formulation clarifies that the role of the CAR assumption is to borrow information across neighboring spatial locations and stabilize estimation, while the biological constraints are imposed directly on the parameter space. (page 23 line 805-815)

2. **The Supplementary note was not clear to the nature of the computation algorithm. Is it MCMC or something else? I would like to get a sense of the runtime of the algorithm and its comparison with other methods.**

**Response:** We thank the reviewer for this helpful comment. We have revised the Supplementary Note to clarify the nature of the computational algorithm (Supplementary 363-372). SWIMR does not use MCMC. Instead, it uses a deterministic optimization procedure based on multiplicative update rules to estimate the model parameters. Specifically, SWIMR alternates between updating the reference-specific cell-type proportions and the reference weights while enforcing nonnegativity and normalization constraints, as described in the revised Supplementary Note (line 363-372).

In the revised manuscript, we added runtime and peak memory benchmarks comparing SWIMR with CARD, RCTD, SCDC, and cell2location across three spatial transcriptomics datasets, including human heart ISS, mouse olfactory bulb ST, and human breast cancer Visium data. The results (Supplementary Figure 32 and Supplementary Table 5) show that SWIMR remains computationally efficient, with runtime comparable to CARD despite integrating multiple scRNA-seq references, whereas CARD uses only a single reference. SWIMR was also substantially faster than RCTD, SCDC, and cell2location across the evaluated datasets. Notably, SCDC did not complete on the largest dataset, further supporting the scalability of SWIMR for large spatial transcriptomics analyses. We also included this result in the discussion (page 19 line 646-651, Supplementary Figure 32 and Supplementary Table 5).

**Supplementary Figure 32. Computation time and peak memory usage for each deconvolution method across three spatial transcriptomics datasets.** Computation time is shown in minutes in the top panel, and peak memory usage is shown in MB in the bottom panel. SWIMR, CARD, RCTD, and SCDC were run in R using a single CPU core on an Intel(R) Xeon(R) Platinum 8268 CPU @ 2.90GHz processor under Ubuntu 22.04.5 LTS. cell2location was run in Python using an NVIDIA H100 NVL GPU. For methods run separately across multiple references, runtimes were summed across reference-specific runs, while peak memory usage was reported as the maximum peak memory observed across runs because the runs were performed sequentially. For cell2location, the memory panel reports CPU peak memory increase to make it comparable with the R-based methods; GPU memory allocation was recorded separately and was not included in the main memory comparison because CPU RAM and GPU memory are distinct hardware resources. SCDC was not completed for the human breast cancer Visium dataset due to its long runtime, exceeding three days.

3. **One class of comparator methods I would like to see in the simulations and examples would be a `consensus approach’ in which each of the reference panels is used for deconvolution, and then some ensembling over results is done.**

**Response:** We thank the reviewer for this helpful suggestion. Following the reviewer’s comment, we implemented a consensus/ensemble comparator in which each reference panel is first used separately for deconvolution, and the resulting estimates are then combined across references.

Following the rationale used in SCDC, we evaluated each reference according to how well each reference reconstructs the spatial data. Specifically, for each reference, we calculated the sum of squared errors (SSE) between the observed spatial gene expression and the reconstructed expression based on the estimated cell-type proportions. We then assigned each reference a weight proportional to the inverse SSE, so that references with better reconstruction accuracy contributed more to the final ensemble estimate. We have added this ensemble comparator to discussion and supplementary material (Supplementary Note 4 and Supplementary Figures 33 and 34) and included comparisons with SWIMR in both the simulation studies and the human heart data analysis.

In the simulation studies, under the complete-overlap scenario, the RMSEs were 0.085 for SWIMR, 0.104 for the CARD-based ensemble, 0.190 for the RCTD-based ensemble, and 0.079 for the cell2location-based ensemble. Under the partial-overlap scenario, the corresponding RMSEs were 0.072, 0.140, 0.167, and 0.185, respectively, while under the non-overlap scenario they were 0.069, 0.216, 0.240, and 0.239, respectively. We also evaluated the consensus strategy in the developing human heart dataset. Here, SWIMR and the CARD-based ensemble showed comparable overall accuracy, with both achieving an RMSE of 0.071, compared with 0.102 for the RCTD-based ensemble and 0.171 for the cell2location-based ensemble. Overall, the ensemble approaches generally improved upon the corresponding single-reference methods, indicating that combining information across references can enhance deconvolution performance. However, SWIMR achieved lower RMSE than the ensemble approaches in most settings, particularly in the partial-overlap and non-overlap scenarios, supporting the benefit of jointly modeling multiple references rather than combining independently derived estimates using a post hoc consensus rule.

An important distinction between the ensemble strategy and SWIMR is that the consensus approach assigns a single global weight to each reference based on overall reconstruction error and therefore assumes that the relative usefulness of each reference is constant across the tissue. In contrast, SWIMR estimates location-specific reference weights, allowing the contribution of each reference to vary with local tissue context. This flexibility can provide additional biological insight into where particular references are most compatible with the observed spatial transcriptomic profile. For example, in the developing human heart analysis, SWIMR assigned substantially greater weight to the internal reference, which was more closely matched to the spatial dataset, while assigning a smaller contribution to the external reference. More generally, these spatially varying weights can reveal tissue regions in which different references better represent local cell states or microenvironments, information that cannot be obtained from a global consensus weight.

We also added this to the discussion (page 19 line 656-663).

**Supplementary Figure 33. Root mean squared error (RMSE) of cell type proportion estimates for all methods and simulation scenarios including the consensus ensemble of single-reference based methods.**

**Supplementary Figure 34. RMSE comparison for all deconvolution methods including the consensus ensemble of single-reference based methods for developing human heart.**

**References**

1.	Zeisel A, Hochgerner H, Lönnerberg P, et al. Molecular Architecture of the Mouse Nervous System. *Cell*. 2018;174(4):999-1014.e22. doi:10.1016/j.cell.2018.06.021

2.	Mizrak D, Levitin HM, Delgado AC, et al. Single-Cell Analysis of Regional Differences in Adult V-SVZ Neural Stem Cell Lineages. *Cell Reports*. 2019;26(2):394-406.e5. doi:10.1016/j.celrep.2018.12.044

3.	Zhang Z, Mathew D, Lim TL, et al. Recovery of biological signals lost in single-cell batch integration with CellANOVA. *Nature Biotechnology*. 2025;43(11):1861-1877. doi:10.1038/s41587-024-02463-1

4.	Welch JD, Kozareva V, Ferreira A, Vanderburg C, Martin C, Macosko EZ. Single-Cell Multi-omic Integration Compares and Contrasts Features of Brain Cell Identity. *Cell*. 2019;177(7):1873-1887.e17. doi:10.1016/j.cell.2019.05.006

5.	Korsunsky I, Millard N, Fan J, et al. Fast, sensitive and accurate integration of single-cell data with Harmony. *Nature Methods*. 2019;16(12):1289-1296. doi:10.1038/s41592-019-0619-0

6.	Stuart T, Butler A, Hoffman P, et al. Comprehensive Integration of Single-Cell Data. *Cell*. 2019;177(7):1888-1902.e21. doi:10.1016/j.cell.2019.05.031
