# Supplementary material for: Enhancing cell type deconvolution in spatial transcriptomics through incorporating multiple reference panels
Source: PLoS Genet. 2026 Nov 2;22(11):e1012345. doi: 10.1371/journal.pgen.1012345 (PMC13726986; doi:10.1371/journal.pgen.1012345)
Supplement: Peer review history

# Peer review: Decision Letter 0

**DOI:** 10.1371/journal.pgen.1012345.r001

**Authors:** Michael Epstein, Kai Wang

28 Apr 2026

PGENETICS-D-26-00086

Enhancing cell type deconvolution in spatial transcriptomics through incorporating multiple reference panels

PLOS Genetics

Dear Dr. Zhou,

Thank you for submitting your manuscript to PLOS Genetics. After careful consideration, we feel that it has merit but does not fully meet PLOS Genetics's publication criteria as it currently stands. Therefore, we invite you to submit a revised version of the manuscript that addresses the points raised during the review process.

Please submit your revised manuscript by Jun 27 2026 11:59PM. If you will need more time than this to complete your revisions, please reply to this message or contact the journal office at plosgenetics@plos.org. When you're ready to submit your revision, log on to https://www.editorialmanager.com/pgenetics/ and select the 'Submissions Needing Revision' folder to locate your manuscript file.

Please include the following items when submitting your revised manuscript:

* A letter that responds to each point raised by the editor and reviewer(s). You should upload this letter as a separate file labeled 'Response to Reviewers'. This file does not need to include responses to any formatting updates and technical items listed in the 'Journal Requirements' section below.

* A marked-up copy of your manuscript that highlights changes made to the original version. You should upload this as a separate file labeled 'Revised Manuscript with Track Changes'.

* An unmarked version of your revised paper without tracked changes. You should upload this as a separate file labeled 'Manuscript'.

If you would like to make changes to your financial disclosure, competing interests statement, or data availability statement, please make these updates within the submission form at the time of resubmission. Guidelines for resubmitting your figure files are available below the reviewer comments at the end of this letter.

We look forward to receiving your revised manuscript.

Kind regards,

Kai Wang

Academic Editor

PLOS Genetics

Michael P. Epstein

Section Editor

PLOS Genetics

Aimée Dudley

Editor-in-Chief

PLOS Genetics

Anne Goriely

Editor-in-Chief

PLOS Genetics

**Journal Requirements:**

1) Please provide an Author Summary. This should appear in your manuscript between the Abstract (if applicable) and the Introduction, and should be 150-200 words long. The aim should be to make your findings accessible to a wide audience that includes both scientists and non-scientists. Sample summaries can be found on our website under Submission Guidelines:

https://journals.plos.org/plosgenetics/s/submission-guidelines#loc-parts-of-a-submission

2) We have noticed that you have uploaded Supporting Information files, but you have not included a list of legends. Please add a full list of legends for your Supporting Information files after the references list.

3) Please amend your detailed Financial Disclosure statement. This is published with the article. It must therefore be completed in full sentences and contain the exact wording you wish to be published.

State what role the funders took in the study. If the funders had no role in your study, please state: "The funders had no role in study design, data collection and analysis, decision to publish, or preparation of the manuscript.".

If you did not receive any funding for this study, please simply state: u201cThe authors received no specific funding for this work.u201d

**Reviewers' comments:**

Reviewer's Responses to Questions

**Comments to the Authors:**

**Please note here if the review is uploaded as an attachment.**

Reviewer #1: Please see the attached report.

Reviewer #2: The choice of reference in spatial transcriptomic deconvolution is often a headache in practice. The proposed SWIMR method is timely, and it has been evaluated by comprehensive studies. I expect it will be widely used. My comments are mostly for clarifications.

1. Clarification of simulation setup.

The authors "assigned one scRNA-seq reference to simulate gene expression in the GCL and ONL regions, and a second reference for the MCL region". It is not clear how similar are the two references? They both cover the same 6 cell types? In real data, the cells classified as the same cell types from two studies could have different expression, though there can be many reasons. For example, it could be batch effect, or it could be biology, e.g., the difference in the proportion of cell subtypes or cell states.

2. the author claim that the most challenging case is "the no-overlap scenario, where each reference contains a completely distinct set of cell types."

It might be helpful to elaborate on this. Why is this the most challenging cases? I am thinking the criteria to decide which reference is used. If two references both have one cell types, it might be harder to decide which reference to use? or the criteria is the deconvolution accuracy.

3. For the 1st real data analysis. Two references are used. "The internal reference, directly obtained from the original study, shares the same set of cell types as the ST data. The external reference, generated using 10x Genomics technology, was manually annotated to assign cell types".

It would be helpful to clarify in the main text here, rather than in methods, what platform or technology of the internal reference. It would be also helpful to evaluate the consistency of cell type-specific gene expression between the references.

4. for the Human Lung Cancer 10x Visium data. It is interesting that "Smart-seq2 and inDrop contributed most prominently to the deconvolution" To further validate these findings, it would helpful if you chose some spots that are dominated by one cell type, and assume they represent the cell type-specific gene expression from Visium. Then compare them with reference from 10x platform, Smart-seq2 or inDrop?

5. For cancer studies, a challenge is that the cancer transcriptome from different patients is often very different. This may be a situation, for example, beneficial to combine different references that represent different tumor subtypes, and thus a situation that SWIMR could be very helpful. For the lung cancer and breast cancer data, how many patients are there and are the results (in terms of utilization of references) differ across patients?

6. In general, using data from different references means the batch effect may play a role. Any comments or suggestions on how to handle such batch effect? Should users try to evaluate batch effect, such as align the reference with each other before running SWIMR?

A minor concern.

1. the authors claim that EPCAM is an onco-gene. The relatively higher expression of EPCAM may indicate more aggressive breast cancer metastasis (based on the reference cited in this paper), but in general, it is highly expressed in both tumor and normal epithelial. It might be better to rephrase it to make it more specific based on the evidence from the reference, such as "EPCAM, which is known to be over-expressed in metastatic breast cancer"

Reviewer #3: Review of “Enhancing cell type deconvolution in spatial transcriptomics through incorporating multiple reference panels”

In this article, the authors tackle the problem of performing deconvolution of spatial transcriptomics gene expression data using multiple reference panels. This involves formulating a deconvolution model (represented in equations (1) and (2)) and evaluating it compared to the SCDC method and some existing single-reference methods. This is done very comprehensively using simulated and real datasets. The paper tackles a very important problem, and I have the following questions.

1. Several aspects of the model in (1) and (2) do not make sense.

- The authors constrain P and Q to be nonnegative, but then use a Gaussian model for P. This does not make sense conceptually, and I was wondering why the initial nonnegativity constraints were need.

- The authors specify a conditional model (2) for the P’s but then use it to imply a joint distribution. How that works is unclear to me.

2. The Supplementary note was not clear to the nature of the computation algorithm. Is it MCMC or something else? I would like to get a sense of the runtime of the algorithm and its comparison with other methods.

3. One class of comparator methods I would like to see in the simulations and examples would be a `consensus approach’ in which each of the reference panels is used for deconvolution, and then some ensembling over results is done.

**********

**Have all data underlying the figures and results presented in the manuscript been provided?**

Large-scale datasets should be made available via a public repository as described in the *PLOS Genetics*  data availability policy, and numerical data that underlies graphs or summary statistics should be provided in spreadsheet form as supporting information.

Reviewer #1: Yes

Reviewer #2: Yes

Reviewer #3: Yes

**********

PLOS authors have the option to publish the peer review history of their article (what does this mean?). If published, this will include your full peer review and any attached files.

If you choose “no”, your identity will remain anonymous but your review may still be made public.

**Do you want your identity to be public for this peer review?**  For information about this choice, including consent withdrawal, please see our Privacy Policy.

Reviewer #1: No

Reviewer #2: **Yes:**  Wei Sun

Reviewer #3: No

[NOTE: If reviewer comments were submitted as an attachment file, they will be attached to this email and accessible via the submission site. Please log into your account, locate the manuscript record, and check for the action link "View Attachments". If this link does not appear, there are no attachment files.]

**Figure resubmission:**

**Reproducibility:**

To enhance the reproducibility of your results, we recommend that authors of applicable studies deposit laboratory protocols in protocols.io, where a protocol can be assigned its own identifier (DOI) such that it can be cited independently in the future. Additionally, PLOS ONE offers an option to publish peer-reviewed clinical study protocols. Read more information on sharing protocols at https://plos.org/protocols?utm_medium=editorial-email&utm_source=authorletters&utm_campaign=protocols

Attachment Submitted filename: Review Comments.pdf

# Author comment: Author response to Decision Letter 1

**DOI:** 10.1371/journal.pgen.1012345.r002

21 Sep 2026

Attachment Submitted filename: response_letter.docx

# Peer review: Decision Letter 1

**DOI:** 10.1371/journal.pgen.1012345.r003

**Authors:** Michael Epstein, Kai Wang

11 Oct 2026

Dear Dr Zhou,

Conditional on addressing a few minor comments from R1, we are pleased to inform you that your manuscript entitled "Enhancing cell type deconvolution in spatial transcriptomics through incorporating multiple reference panels" has been editorially accepted for publication in PLOS Genetics. Congratulations!

Before your submission can be formally accepted and sent to production you will need to complete our formatting changes, which you will receive in a follow up email. Please be aware that it may take several days for you to receive this email; during this time no action is required by you. Please note: the accept date on your published article will reflect the date of this provisional acceptance, but your manuscript will not be scheduled for publication until the required changes have been made.

Once your paper is formally accepted, an uncorrected proof of your manuscript will be published online ahead of the final version, unless you’ve already opted out via the online submission form. If, for any reason, you do not want an earlier version of your manuscript published online or are unsure if you have already indicated as such, please let the journal staff know immediately at plosgenetics@plos.org.

In the meantime, please log into Editorial Manager at https://www.editorialmanager.com/pgenetics/, click the "Update My Information" link at the top of the page, and update your user information to ensure an efficient production and billing process. Note that PLOS requires an ORCID iD for all corresponding authors. Therefore, please ensure that you have an ORCID iD and that it is validated in Editorial Manager. To do this, go to ‘Update my Information’ (in the upper left-hand corner of the main menu), and click on the Fetch/Validate link next to the ORCID field.  This will take you to the ORCID site and allow you to create a new iD or authenticate a pre-existing iD in Editorial Manager.

If you have a press-related query, or would like to know about making your underlying data available (as you will be aware, this is required for publication), please see the end of this email. If your institution or institutions have a press office, please notify them about your upcoming article at this point, to enable them to help maximise its impact. Inform journal staff as soon as possible if you are preparing a press release for your article and need a publication date.

Thank you again for supporting open-access publishing; we are looking forward to publishing your work in PLOS Genetics!

Yours sincerely,

Kai Wang

Academic Editor

PLOS Genetics

Michael Epstein

Section Editor

PLOS Genetics

Aimée Dudley

Editor-in-Chief

PLOS Genetics

Anne Goriely

Editor-in-Chief

PLOS Genetics

www.plosgenetics.org

BlueSky: @plos.bsky.social

----------------------------------------------------

Comments from the reviewers (if applicable):

Please proofread for inconsistencies and typos to satisfy one reviewer's comments.

Reviewer's Responses to Questions

**Comments to the Authors:**

**Please note here if the review is uploaded as an attachment.**

Reviewer #1: see attached file

Reviewer #2: the authors have addressed all my comments.

Reviewer #3: The authors addressed my comments.

**********

**Have all data underlying the figures and results presented in the manuscript been provided?**

Large-scale datasets should be made available via a public repository as described in the *PLOS Genetics*  data availability policy, and numerical data that underlies graphs or summary statistics should be provided in spreadsheet form as supporting information.

Reviewer #1: None

Reviewer #2: Yes

Reviewer #3: Yes

**********

PLOS authors have the option to publish the peer review history of their article (what does this mean?). If published, this will include your full peer review and any attached files.

If you choose “no”, your identity will remain anonymous but your review may still be made public.

**Do you want your identity to be public for this peer review?**  For information about this choice, including consent withdrawal, please see our Privacy Policy.

Reviewer #1: No

Reviewer #2: No

Reviewer #3: **Yes:**  Debashis Ghosh

----------------------------------------------------

**Data Deposition**

If you have submitted a Research Article or Front Matter that has associated data that are not suitable for deposition in a subject-specific public repository (such as GenBank or ArrayExpress), one way to make that data available is to deposit it in the Dryad Digital Repository. As you may recall, we ask all authors to agree to make data available; this is one way to achieve that. A full list of recommended repositories can be found on our website.

The following link will take you to the Dryad record for your article, so you won't have to re‐enter its bibliographic information, and can upload your files directly:

http://datadryad.org/submit?journalID=pgenetics&manu=PGENETICS-D-26-00086R1

More information about depositing data in Dryad is available at http://www.datadryad.org/depositing. If you experience any difficulties in submitting your data, please contact help@datadryad.org for support.

Additionally, please be aware that our data availability policy requires that all numerical data underlying display items are included with the submission, and you will need to provide this before we can formally accept your manuscript, if not already present.

----------------------------------------------------

**Press Queries**

If you or your institution will be preparing press materials for this manuscript, or if you need to know your paper's publication date for media purposes, please inform the journal staff as soon as possible so that your submission can be scheduled accordingly. Your manuscript will remain under a strict press embargo until the publication date and time. This means an early version of your manuscript will not be published ahead of your final version. PLOS Genetics may also choose to issue a press release for your article. If there's anything the journal should know or you'd like more information, please get in touch via plosgenetics@plos.org.

Attachment Submitted filename: PLOS_Genetics_2nd_Review.pdf

# Editor report: Acceptance letter

**DOI:** 10.1371/journal.pgen.1012345.r004

**Authors:** Michael Epstein, Kai Wang

PGENETICS-D-26-00086R1

Enhancing cell type deconvolution in spatial transcriptomics through incorporating multiple reference panels

Dear Dr Zhou,

We are pleased to inform you that your manuscript entitled "Enhancing cell type deconvolution in spatial transcriptomics through incorporating multiple reference panels" has been formally accepted for publication in PLOS Genetics! Your manuscript is now with our production department and you will be notified of the publication date in due course.

The corresponding author will soon be receiving a typeset proof for review, to ensure errors have not been introduced during production. Please review the PDF proof of your manuscript carefully, as this is the last chance to correct any errors. Please note that major changes, or those which affect the scientific understanding of the work, will likely cause delays to the publication date of your manuscript.

Soon after your final files are uploaded, unless you have opted out or your manuscript is a front-matter piece, the early version of your manuscript will be published online. The date of the early version will be your article's publication date. The final article will be published to the same URL, and all versions of the paper will be accessible to readers.

For Research Articles, you will receive an invoice from PLOS for your publication fee after your manuscript has reached the completed accept phase. If you receive an email requesting payment before acceptance or for any other service, this may be a phishing scheme. Learn how to identify phishing emails and protect your accounts at https://explore.plos.org/phishing.

Thank you again for supporting PLOS Genetics and open-access publishing. We are looking forward to publishing your work!

With kind regards,

Sharmila Kamatchi

PLOS Genetics

On behalf of:

The PLOS Genetics Team

Carlyle House, Carlyle Road, Cambridge CB4 3DN | United Kingdom

plosgenetics@plos.org | +44 (0) 1223-442823

plosgenetics.org | Twitter: @PLOSGenetics
